# The Alteration of Emotion Regulation Precedes the Deficits in Interval Timing in the BACHD Rat Model for Huntington Disease

**DOI:** 10.3389/fnint.2018.00014

**Published:** 2018-05-09

**Authors:** Daniel Garces, Nicole El Massioui, Charlotte Lamirault, Olaf Riess, Huu P. Nguyen, Bruce L. Brown, Valérie Doyère

**Affiliations:** ^1^The Graduate Center, City University of New York, New York, NY, United States; ^2^Institut des Neurosciences Paris-Saclay (Neuro-PSI), Université Paris Sud, CNRS, Université Paris-Saclay, Orsay, France; ^3^Institute of Medical Genetics and Applied Genomics, University of Tübingen, Tübingen, Germany; ^4^Center for Rare Diseases, University of Tübingen, Tübingen, Germany; ^5^Department of Human Genetics, Ruhr University Bochum, Bochum, Germany; ^6^Queens College, City University of New York, New York, NY, United States

**Keywords:** interval timing, Huntington disease, temporal bisection, peak interval, stress

## Abstract

Huntington disease (HD) is an autosomal dominantly inherited, progressive neurodegenerative disorder which is accompanied by executive dysfunctions and emotional alteration. The aim of the present study was to assess the impact of emotion/stress on on-going highly demanding cognitive tasks, i.e., temporal processing, as a function of age in BACHD rats (a “full length” model of HD). Middle-aged (4–6 months) and old (10–12 months) rats were first trained on a 2 vs. 8-s temporal discrimination task, and then exposed to a series of bisection tests under normal and stressful (10 mild unpredictable foot-shocks) conditions. The animals were then trained on a peak interval task, in which reinforced fixed-interval (FI) 30-s trials were randomly intermixed with non-reinforced probe trials. After training, the effect of stress upon time perception was again assessed. Sensitivity to foot-shocks was also assessed independently. The results show effects of both age and genotype, with largely greater effects in old BACHD animals. The older BACHD animals had impaired learning in both tasks, but reached equivalent levels of performance as WT animals at the end of training in the temporal discrimination task, while remaining impaired in the peak interval task. Whereas sensitivity to foot-shock did not differ between BACHD and WT rats, delivery of foot-shocks during the test sessions had a disruptive impact on temporal behavior in WT animals, an effect which increased with age. In contrast, BACHD rats, independent of age, did not show any significant disruption under stress. In conclusion, BACHD rats showed a disruption in temporal learning in late symptomatic animals. Age-related modification in stress-induced impairment of temporal control of behavior was also observed, an effect which was greatly reduced in BACHD animals, thus confirming previous results suggesting reduced emotional reactivity in HD animals. The results suggest a staggered onset in cognitive and emotional alterations in HD, with emotional alteration being the earliest, possibly related to different time courses of degeneration in cortico-striatal and amygdala circuits.

## Introduction

Huntington disease (HD) is an autosomal dominantly inherited and progressive neurodegenerative disorder caused by an expanded CAG repeat of variable length in exon 1 of the gene encoding the protein huntingtin. HD causes degeneration of the medium spiny neurons of the striatum (Graveland et al., 1985), but also rapid neuronal death in the cerebral cortex and limbic structures (Vonsattel, 2008). Clinically, HD is characterized by a triad of motor, cognitive and psychiatric problems, with non-motor deficits being generally dominated by executive function disorders (Watkins et al., 2000; Minati et al., 2011; Holl et al., 2013). Moreover, emotional disorders, psychosis, and personality changes with behavioral and emotional dyscontrol have been commonly described in HD. Recognition of emotions (mainly negative but also positive) is altered in premanifest as well as manifest HD (Sprengelmeyer et al., 1996; Paradiso et al., 2008; Snowden et al., 2008; Eddy et al., 2011; Robotham et al., 2011; Henley et al., 2012; Novak et al., 2012; Rees et al., 2014). Recently, alterations in subjective emotional experiences have been evidenced in addition to emotion recognition deficits, even though conceptual understanding of emotions remains relatively intact (Kordsachia et al., 2017) and emotion representations on the level of internal experience might be spared (Trinkler et al., 2017).

Several rodent models of HD show an early progressive HD-like phenotype with neuronal degeneration in the striatum and cortices and early development of cognitive and emotional symptoms. Anxiety and fear reactivity changes, depending on the polyglutamine length in mutant huntingtin and stages of the disease have been described (File et al., 1998; Hickey et al., 2005, 2008; Ciamei and Morton, 2008; Rudenko et al., 2009). Emotional blunting was observed in pre-symptomatic tgHD rats which exhibit a late adult HD phenotype (51 CAG repeats under control of the native rat huntingtin promoter; Von Hörsten et al., 2003; Faure et al., 2011, 2013) that may be related to dysfunction of the central nucleus of amygdala (Faure et al., 2011). Similarly, BACHD rats (97 mixed CAG-CAA repeats), expressing full length mutant human huntingtin (Yu-Taeger et al., 2012) showed decreased impact of an initial stress on later decision making performance during the late stages of the disease (Adjeroud et al., 2015), suggesting altered susceptibility to emotion that may be related to dysfunction of the central nucleus of amygdala (Lamirault et al., 2017). The effects were weak, however, possibly due to the procedural separation of decision making and stress phases.

To further assess the impact of emotion on executive functions in HD, we aimed at investigating emotion regulation during highly demanding cognitive tasks in BACHD rats. Interval timing, i.e., processing of durations in the second-to-minute range, is a complex cognitive task which is thought to require cortico-striatal network integrity (Buhusi and Meck, 2005). Timing functions are altered in HD patients, with decreased timing precision, with or without decreased accuracy (Paulsen et al., 2004; Beste et al., 2007; Hinton et al., 2007; Cope et al., 2014; Rao et al., 2014; Righi et al., 2016; Agostino et al., 2017). While these reports converge in showing increased deficits with the proximity to onset of disease’s symptoms, Beste et al. (2007) suggested that the time onset of the deficits may depend on motor involvement in the task, i.e., estimation vs. production. However, timing deficits may appear at a pre-symptomatic stage. In the tgHD rat model of HD, temporal estimation was altered from pre-symptomatic stages with poorer temporal sensitivity as early as 4 months of age, well before detection of overt motor deficits (Brown et al., 2011; Höhn et al., 2011). Similarly, disrupted temporal control (variability) in a peak interval production procedure with intact temporal accuracy was described in transgenic R6/2 mouse model of HD at 4–7 weeks of age (Balci et al., 2009). There is a paucity of data on the relation of timing deficits to disease progression in animals.

The aim of the present study was to assess the impact of emotion/stress on on-going temporal processing as a function of age. BACHD and WT rats were trained at two different ages, 4 and 10 months, in order to compare interval timing and its disruptability by stress at both early and later symptomatic stages. Two classical timing tasks were employed: a temporal estimation task involving time discrimination (bisection procedure) and a temporal production task using the peak-interval procedure. Mild stress was produced by introducing unpredictable unescapable foot-shocks during the tasks.

## Materials and Methods

### Animals

The study was performed on two cohorts of 24 wild type (WT) and 24 BACHD male rats. These were acquired from in-house breeding, using hemizygous BACHD males from the TG5 line (Yu-Taeger et al., 2012) paired with WT females (Charles River, Germany). All animals were of Sprague-Dawley background. Animals were genotyped according to previously published protocols (Yu-Taeger et al., 2012). They were either 4 (“Middle-Aged rats”) or 10 months old (“Old” rats), equally distributed among genotypes (*n* = 12 per group) at the beginning of the experiment. Animals were housed two or three per cage in a room with controlled temperature and humidity with a 12 h–12 h day–night cycle (lights on at 8 am). The experiments were performed during the “light” cycle, 5–6 days per week. The animals had free access to water, and were food-restricted and maintained at 85% of their *ad libitum* weight. Before being enrolled in the present study, all the animals had experienced several tasks, either exploratory, food motivated instrumental or Pavlovian aversive conditioning (with foot shocks) and extinction, followed by a 2–3 weeks resting while progressively adjusted to their food-restriction regime. All experiments were conducted in accordance with the guidelines established by the European Communities Council Directive (2010/63/EU Council Directive Decree) for compliance and use of laboratory animals. The protocol was approved by the ethical committee Paris-Sud and Centre (CEEA N°59).

### Apparatus

Eight operant boxes (31 cm × 25 cm × 31 cm; Coulbourn Instruments, United States) in soundproofed ventilated chambers (background noise 65 dB) were controlled with a Graphic State program (Coulbourn Instruments, United States). The operant boxes were equipped with two retractable levers (4-cm from the floor) positioned 8 cm on centers to the left and right side of the magazine where 45-mg grain-based food pellets (BioServ) were delivered, a speaker above the magazine, and a red light (4 lux) as a house light. A tone (1 kHz, 80 dB) served as a timing cue.

### Temporal Discrimination

In the temporal discrimination task, the rats were trained to press the left or right lever after either 2-s or 8-s tone durations. The protocol was identical to the one in previous studies (Callu et al., 2009; Höhn et al., 2011).

#### Pretraining

Twenty-four hours after a 30-min session of magazine training (variable interval 30 s), rats were trained to press each lever under a continuous reinforcement schedule until 50 reinforcers were earned in a 30-min session (one session for each lever, 24 h apart). If the criterion was not met on the first session, a second session with the same lever was run at the end of the day.

#### Temporal Discrimination Training

Animals were then trained on a 2 vs. 8 s temporal discrimination, starting with 3 days of 100% forced-choice trials, 3 days of 50% forced-choice/50% free-choice trials, and followed by 6 days of 100 free-choice trials. For the forced-choice trials, only the correct lever (left or right) paired with the corresponding tone duration (2 or 8 s) was presented, whereas both levers were presented on free-choice trials. Each session was composed of 80 trials, with two blocks of 40 trials (20 forced-choice/20 free-choice, or 40 free-choice) in random order for each duration with equal probability, with the constraint that no more than three trials of a given duration could occur successively. The same random order was used for all the animals for a given session. The lever/duration assignment was counterbalanced within each group of genotype and age. The intertrial interval (ITI) ranged from 20 to 40 s, with a mean of 30 s. The lever was retracted immediately after a response, or after 10 s in case of no response.

#### Bisection Tests

A psychophysical choice procedure was then conducted in which five intermediate durations (2.5, 3.2, 4, 5, and 6.3 s, 12 trials each) were added to the two anchor trained durations (2 and 8 s, 60 trials of each). Correct responses to the anchor durations were reinforced, whereas responses to intermediate durations were not. The order of trials was randomized within two blocks of 90 trials (60 training trials and six test trials for each of the five durations). The mean ITI was 20 s (range 10 to 30 s).

#### Bisection Tests Under Stress

A bisection test was run during which 10 unpredictable mild scrambled foot-shocks (0.25 mA, 0.5 s) were interspersed between the trials during the ITI. The session started with one trial of each of the anchor durations, followed 10 s later by the first foot-shock, and after an ITI elapsed, the remaining trials and foot-shocks were pseudo-randomly distributed (10–20 trials range between shocks, with a minimum of 10 s between a shock and a cue). A normal bisection test without shocks was run 24 h later. This cycle was repeated 3 days later.

#### Analysis

Response location and latency were recorded for each trial. Discrimination data were analyzed as percentage of correct responses across all free-choice trials. For bisection tests, the proportion of responses on the lever assigned as correct for the long-duration stimulus on all trials with response was calculated. The bisection functions relating proportion of “long” responses to stimulus duration were averaged across sessions within a condition for all trials with a response, within each age for each rat, and analyzed with the pseudologistic model fit (Killeen et al., 1997) using Prism software (GraphPad Software) and assuming negligible contribution of constant and Poisson sources of variability (Allan, 2002). The proportion of variance accounted for by the fit, the point of subjective equality [PSE, stimulus value corresponding to p(long) = 0.5], and the temporal sensitivity parameter (gamma) were estimated for each rat. Gamma, which is proportional to the Weber fraction, is inversely related to temporal sensitivity.

### Peak Interval

After a 3-day break, animals were then trained in the same operant boxes on a peak interval (PI) task in which a reinforcer was available on some trials after a fixed time (30 s) from the start of the tone timing cue. The tone frequency in the PI task was changed to 7 kHz to help the animal differentiate the two tasks.

#### Training

Animals were first exposed to a 50-pellet continuous reinforcement session, in which a pellet was delivered contingent upon a response on the lever that corresponded to the short duration in the preceding temporal discrimination task. That session was then followed by peak interval sessions in which fixed-interval (FI) and probe trials were intermixed, with a mean ITI of 20 s on average (range 10–30 s). In the FI trials, the tone lasted for a maximum of 60 s and the first lever press after 30 s from tone onset was reinforced and the tone terminated. In probe trials, the tone lasted for 90 s and lever presses were not reinforced. After five sessions with 49 FI and 8 Probe trials, rats were trained for 14 sessions with 37 FI and 18 Probe trials.

#### Peak Interval Under Stress

Twenty-four hours after the last training session, a PI session (with 37 FI and 18 Probe trials) was run during which 10 unpredictable mild foot-shocks (0.25 mA, 0.5 s) were delivered randomly during the ITI, with a range from 1 to12 trials between shocks, and a minimum of 10 s between a foot-shock and a cue onset. The session started with an FI trial followed, 10 s after the delivery of reinforcement, by the first foot-shock. A final PI test session (no shock) was run 24 h later for assessing long-term effects of the stress session.

#### Analysis

Data from only probe trials were analyzed. To assess the acquisition of temporal behavior, two analyses were performed: (1) For molar analyses, lever presses per 2-s bins were averaged across trials in each two-session block (i.e., 36 probe trials) from Session 6 to 19. For the last block, parameters of peak time, peak rate, width, and coefficient of variation (width/peak time) were estimated by a Gaussian + ramp fit using a non-linear regression analysis (PeakFit, see Tallot et al., 2016); and (2) For characterization of temporal behavior acquisition, individual trial analyses were performed from Session 6 to 19 in 2-session (i.e., 36 probe trials) blocks. Using a custom Power Basic program (described in Aum et al., 2004), lever presses per 1-s bin for each probe trial were subjected to regression analyses leading to the best fit of a three-state model. Trials were only included in the analysis for which the rate of responding in the second state (*r*_2_) exceeded the rate in the initial state (*r*_1_) and the rate in the final state (*r*_3_). The minimum duration of high-rate state was 4 s, and the minimum duration of the two low-rate states were 1 s each. The first and last bins of the high rate state were taken as the start and stop times, respectively. The difference of these measures was taken as the spread time. Median measures of individual trial performance, as well as interquartile ranges for start and stop times, were obtained for each subject during each session block. Raster plots depicting performance on each Probe trial for individual rats have been included as Supplementary Material.

### Assessment of Sensitivity to Foot-Shock

This test aimed at determining the sensitivity to foot-shocks of WT and BACHD rats. Rats were placed individually in an operant chamber in which electrical foot-shocks could be delivered through a grid floor. On the 1st day, shocks were delivered in an increasing stepwise manner (from 0.06 to 0.26 mA, in 0.02 mA steps), and, on a 2nd day, in a decreasing stepwise manner (from 0.26 to 0.06 mA, in 0.02 mA steps). Each step was repeated three times with a random ITI of 30, 40, or 60 s. The reactivity threshold to shocks, scored by an observer blind to the animal’s genotype, was defined for each rat as the mean (for increasing and decreasing directions) of the lowest shock intensities that elicited a jump response (at least 2 jump responses for 3 same shock intensity). This test was performed on 6-month-old (9 WT and 8 BACHD) and 9-month-old rats (8 WT and 8 BACHD).

### Statistical Analyses

Statistical analyses were run using JASP (JASP Team, 2017) and R (R Core Team, 2017), using an alpha level of 0.05. For mixed ANOVAs involving repeated-measures factors with more than three levels, Mauchly’s sphericity test was conducted; the Huynh-Feldt correction was adopted when the assumption of sphericity was violated. In all figures with error bars, data are presented as mean ± SEM.

## Results

### Temporal Discrimination

One Old BACHD rat did not learn to respond under CRF and was thus eliminated from the entire study. One Middle-Aged WT rat did not learn the 2 vs. 8 s temporal discrimination task (performance at chance level), and was thus discarded from the analysis of the temporal discrimination study.

#### Acquisition of Temporal Discrimination

**FIGURE 1:**
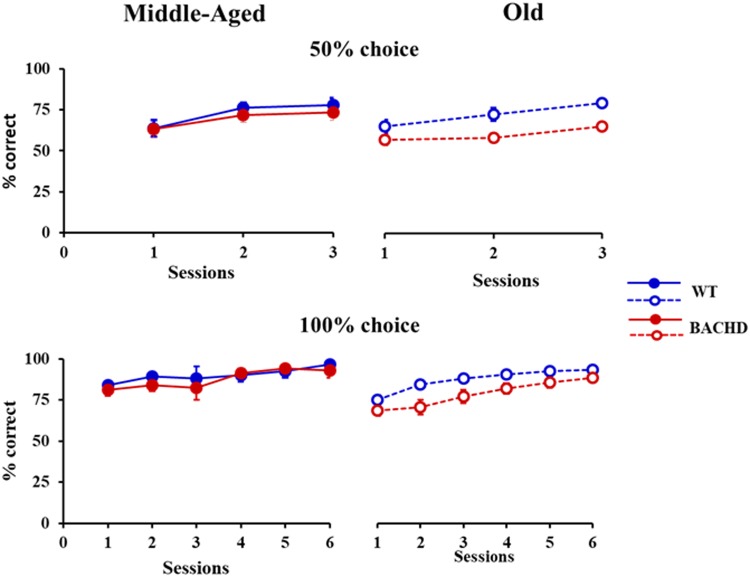
Temporal discrimination learning. Mean percentage (±SEM) of correct responses during sessions with 50% **(top)** and 100% **(bottom)** free-choice for middle-aged (left, filled symbols) and old (right, empty symbols) wild-type (blue) and BACHD (red) rats.

All animals showed learning of the temporal discrimination, reaching high performance on the last 100% Choice training session (>80% of correct responses for all animals except one, which nevertheless had performed at 90% correct responses during the preceding session). However, the speed of learning for both training phases (50 and 100% choice) differed depending on the genotype (**Figure 1**). Mixed ANOVAs with genotype (WT vs. BACHD) and age (Middle-Aged vs. Old) as group factors and session (3 or 6 depending on the training phase) as repeated measure demonstrated a significant effect of genotype [*F*(1,42) = 5.028, *p* = 0.030, ${\text{η}}_{\text{p}}^{2}$ = 0.107 and *F*(1,42) = 4.104, *p* = 0.049, ${\text{η}}_{\text{p}}^{2}$ = 0.089, for 50 and 100% choice, respectively]. A trend for an effect of age was found during training under the 100% choice condition, but the effect did not reach significance [*F*(1,42) = 3.323, *p* = 0.075, ${\text{η}}_{\text{p}}^{2}$ = 0.073]. None of the other effects implicating the factor age or genotype were found to be significant (all *p*s > 0.150). Based on the *a priori* hypothesis that impairments in BACHD rats are expected to worsen with age, in parallel to the evolution of neurodegeneration, we also tested whether the effect of genotype was significant at both ages or only at a specific age. In both training phases, a significant effect of genotype was found in the Old rats [*F*(1,21) = 10.68, *p* = 0.004, ${\text{η}}_{\text{p}}^{2}$ = 0.337 and *F*(1,21) = 7.524, *p* = 0.012, ${\text{η}}_{\text{p}}^{2}$ = 0.264, for 50 and 100% choice training, respectively], but not in the Middle-Aged rats (both *F*s < 1).

**FIGURE 2:**
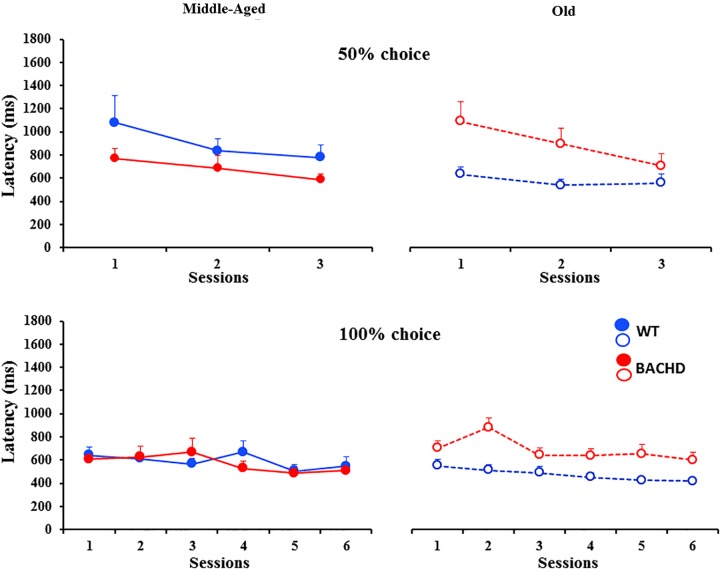
Response latencies (ms) for correct responses during temporal discrimination learning. Legends are identical to the ones in **Figure 1**.

The analysis of response latencies indicated longer latencies for incorrect responses than for correct responses during both training phases (Grand mean; Correct: 745.9 ms and Incorrect: 792.2 ms for 50% choice; Correct: 575.6 ms and Incorrect: 746.1 ms for 100% choice). This difference was significant for 50% choice [*F*(1,43) = 6.213, *p* = 0.017, ${\text{η}}_{\text{p}}^{2}$ = 0.126], with no significant interaction with any of the other factors, session, genotype or age (all *p*s > 0.16). No statistical analysis could be performed for 100%, because of a lack of incorrect responses in some sessions for some animals. When restricted to correct responses (**Figure 2**), the analysis revealed a decrease in latency with session repetition [*F*(1.791,75.224) = 7.823, *p* = 0.001, ${\text{η}}_{\text{p}}^{2}$ = 0.157 and *F*(4.297,180.472) = 7.855, *p* < 0.001, ${\text{η}}_{\text{p}}^{2}$ = 0.158, for 50 and 100%, respectively] and a significant age × genotype interaction [*F*(1,42) = 7.496, *p* = 0.009, ${\text{η}}_{\text{p}}^{2}$ = 0.151 and *F*(1,42) = 4.284, *p* = 0.045, ${\text{η}}_{\text{p}}^{2}$ = 0.093, for 50 and 100%, respectively], no other effects reaching the level of significance. Parsing the interaction, a significant effect of genotype was found for the Old [*F*(1,21) = 6.008, *p* = 0.023, ${\text{η}}_{\text{p}}^{2}$ = 0.222 and *F*(1,21) = 8.625, *p* = 0.008, ${\text{η}}_{\text{p}}^{2}$ = 0.291, for 50 and 100%, respectively], but not the Middle-Aged ones (*p*s > 0.150).

Thus, BACHD rats were slower in learning the temporal discrimination task, and their deficit worsened with age in parallel with the progression of degeneration evidenced by the increase in the animals’ response latency.

#### Bisection Test

**FIGURE 3:**
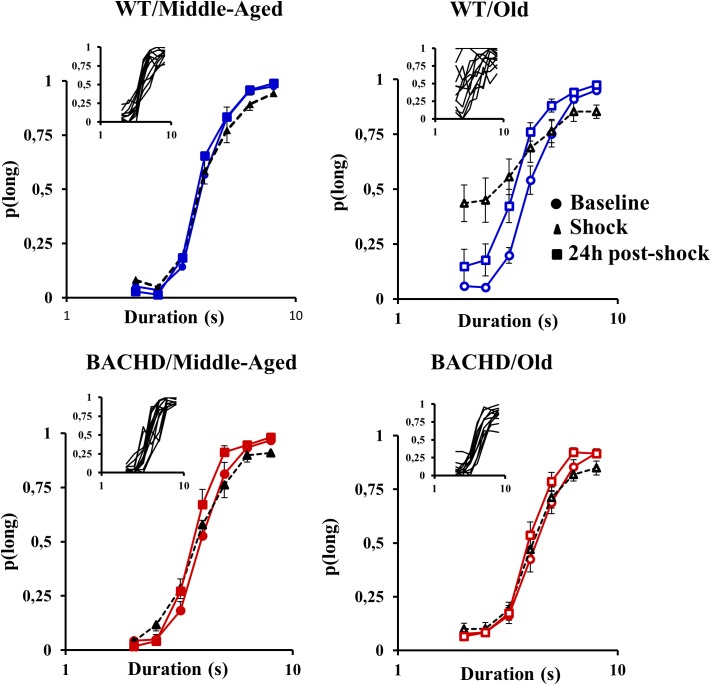
Psychometric functions for the temporal bisection tests: Mean (±SEM) proportion of long responses as a function of the stimulus duration (s) in logarithmic scale in middle-aged and old wild-type **(top)** and in middle-aged and old BACHD rats **(bottom)** during the baseline bisection session (round symbols, colored curves), the bisection session with unpredictable foot-shocks (triangles, black curves) and 24 h post-shock session (squares, colored curves). Inserts represent individual curves for the session with electric foot-shocks.

During the initial (non-shock) bisection tests, all groups of animals responded on close to 100% of trials (mean ± SEM, 99.84 ± 0.11 and 99.20 ± 0.54 for Middle-Aged and Old WT, respectively, and 99.97 ± 0.02 and 98.82 ± 0.84, for Middle-Aged and Old BACHD rats, respectively; with a minimum of 90.63% of responding). **Figure 3** shows the mean proportion of response ‘long’ plotted as a function of stimulus duration averaged for each of the four groups on each of the three bisection tests. A mixed ANOVA of p(long) on the initial bisection test with genotype and age as between group factors and stimulus duration as a repeated measure showed only a trend for a duration x age interaction [*F*(2.505, 105.224) = 2.363, *p* = 0.086, ${\text{η}}_{\text{p}}^{2}$ = 0.053]. No other comparison involving age or genotype was found to be significant or close to significance.

**FIGURE 4:**
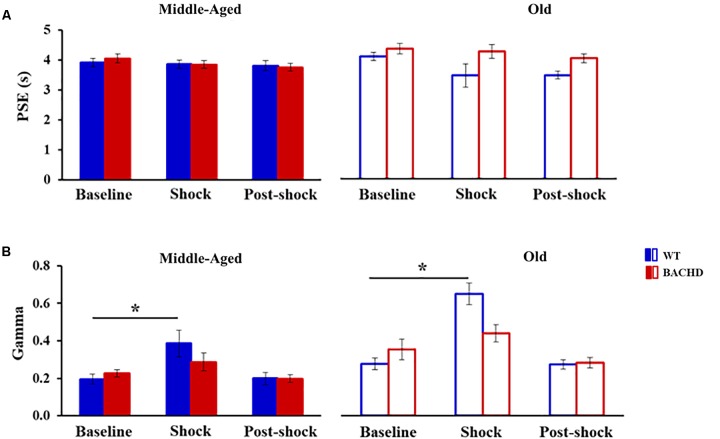
Temporal parameters extracted from the fitted different bisection functions (baseline, session with shocks, 24 h post-shock) for the middle-aged and old wild-type (Blue) and the middle-aged and old BACHD (red) rats: PSE **(A)** and gamma **(B)**. Error bars depict the SEM. Asterisks denote significant differences between sessions within a group (*p* < 0.05).

**Figure 4** presents the group means for the two parameters extracted from the fits of the psychometric functions during all the phases. During the baseline tests, the proportion of variance accounted for by the fit for each rat (*R*^2^) varied from 0.947 to 0.999 and from 0.816 to 0.997 for Middle-Aged and Old WT groups, respectively, and from 0.944 to 0.999 and from 0.900 to 0.993 for Middle-Aged and Old BACHD groups, respectively. The point of subjective equality (PSE, **Figure 4A**) did not differ among groups, indicating no effect of age or genotype on bisection performance (all *p*s > 0.188). With regard to temporal precision, or parameter of discriminability between durations (gamma, **Figure 4B**), only a significant effect of age was found [*F*(1,42) = 8.181, *p* = 0.007, ${\text{η}}_{\text{p}}^{2}$ = 0.163], older rats showing poorer precision (i.e., higher gamma).

#### Effect of Stress

##### Acute effect

Unpredictable mild foot-shocks disrupted the ongoing temporal discrimination behavior differentially depending on both age and genotype. Old WT animals responded less frequently to all durations (mean across durations: 57.79% ± 9.01%, range 13.14–99.29% with 8 animals out of 12 responding less than 60%) than Middle-Aged ones (99.85% ± 0.07%, minimum 99.41%). In contrast, only two Middle-Aged BACHD animals were disrupted with a percent of response of 62.01 and 80.72% (more pronounced for intermediate non-reinforced durations), while all the other BACHD rats, whether Middle-Aged or Old, kept responding (minimum 95.60 and 90.88%, respectively). When responding, the proportion of response ‘long’ was also differentially affected depending on age and genotype (**Figure 3**, black curves). A mixed ANOVA with age (2) and genotype (2) as group factors and duration (7) and phase (baseline vs. stress) as repeated measures revealed a significant duration × phase × age × genotype interaction [*F*(4.046,169.943) = 2.689, *p* = 0.032, ${\text{η}}_{\text{p}}^{2}$ = 0.060]. Parsing by age and genotype, WT animals showed a disrupted bisection function under stress at both ages with a significant effect of phase (baseline vs. stress) for Old rats [*F*(1,66) = 8.311, *p* = 0.015, ${\text{η}}_{\text{p}}^{2}$ = 0.430 for Old WT; *F* < 1 for Middle-Aged WT], and a significant phase × duration interaction for both groups of age [*F*(6,60) = 3.513, *p* = 0.005, ${\text{η}}_{\text{p}}^{2}$ = 0.260 and *F*(3.824,42.061) = 17.629, *p* < 0.001, ${\text{η}}_{\text{p}}^{2}$ = 0.616, for Middle-Aged and Old WT, respectively]. Further analysis restricted to the WT animals showed that Old animals were more disrupted than Middle-Aged ones [phase × duration × age, *F*(3.944,82.828) = 5.369, *p* < 0.001, ${\text{η}}_{\text{p}}^{2}$ = 0.204]. In contrast, BACHD animals did not show significant disruption at either age (all *F*s < 1.216, *p*s > 0.318).

When fitting the individual psychometric functions, *R*^2^ ranged from 0.795 to 0.999 for all rats, except for 4 Old WT which could not be fitted (*R*^2^ ≤ 0.5) and were thus excluded from the analysis of bisection parameters (see insets in **Figure 3** for individual bisection curves under stress). A mixed genotype × age × session ANOVA showed a significant decrease in PSE under stress compared to baseline [**Figure 4A**, 3.85 ± 0.11 vs. 4.06 ± 0.07; *F*(1,38) = 5.433, *p* = 0.025, ${\text{η}}_{\text{p}}^{2}$ = 0.125], with only a trend for the WT rats to have a smaller PSE than BACHD rats [*F*(1,38) = 3.440, *p* = 0.071, ${\text{η}}_{\text{p}}^{2}$ = 0.083]. No other effects were significant [*F*s(1,38) < 2.846, *p*s > 0.10]. With regard to gamma (**Figure 4B**), the analysis showed a significant effect of phase [*F*(1,38) = 27.163, *p* < 0.001, ${\text{η}}_{\text{p}}^{2}$ = 0.417], of age [*F*(1,38) = 19.286, *p* < 0.001, ${\text{η}}_{\text{p}}^{2}$ = 0.337], and a significant phase × genotype interaction [*F*(1,38) = 9.417, *p* = 0.004, ${\text{η}}_{\text{p}}^{2}$ = 0.199], all the other comparisons being not significant [*F*s(1,38) < 2.265, *p*s > 0.141]. Parsing by age and genotype, only WT animals of both ages showed a significant increase in gamma [Middle-Aged WT: *F*(1,10) = 8.726, *p* = 0.014, ${\text{η}}_{\text{p}}^{2}$ = 0.466; Old WT: *F*(1,7) = 24.27, *p* = 0.002, ${\text{η}}_{\text{p}}^{2}$ = 0.776; Middle-Aged BACHD: *F*(1,11) = 1.441, *p* = 0.255; Old BACHD: *F*(1,10) = 1.166, *p* = 0.306]. Further analysis restricted to the WT animals suggested that disruption of temporal precision tended to be worse in Old than Middle-Aged animals [*F*(1,17) = 3.264, *p* = 0.089]. In all, unpredictable mild foot-shocks disrupted ongoing behavior and precision in temporal discrimination in WT animals, with a stronger impact in older animals. In contrast, BACHD animals were undisrupted whatever their age.

##### Long-term effect

The long-term effect of stress due to unpredictable mild foot-shocks was assessed during bisection tests 24 h later. All animals responded in more than 90% of trials, except five Middle-Aged BACHD rats who responded less to non-reinforced intermediate durations than to the anchor durations (close to 50% of responding to the 4-s stimulus duration compared to ∼90% at the anchors). The long-term effect of stress on the proportion of ‘long’ responses (**Figure 3**) was analyzed with a mixed ANOVA with age and genotype as group factors and duration and phase (baseline vs. 24 h post) as repeated measures. Compared to baseline curves, performance at 24 h remained slightly disrupted as reflected by a significant effect of phase [*F*(1,42) = 15.131, *p* < 0.001, ${\text{η}}_{\text{p}}^{2}$ = 0.265] and duration × phase interaction [*F*(3.759,157.883) = 5.980, *p* < 0.001, ${\text{η}}_{\text{p}}^{2}$ = 0.125], as well as a trend for a phase × age × genotype interaction [*F*(1,42) = 3.593, *p* = 0.065]. In addition, there was a significant effect of genotype [mean p(long): 0.497 and 0.539 for BACHD and WT, respectively]; *F*(1,42) = 4.200, *p* = 0.047, ${\text{η}}_{\text{p}}^{2}$ = 0.091], and a significant duration × age interaction [*F*(2.806,117.856) = 4.360, *p* = 0.007, ${\text{η}}_{\text{p}}^{2}$ = 0.094] indicating steeper functions in Middle-Aged animals, as compared to Old ones. Parsing by groups, old animals of both genotypes were disrupted 24 h after the stress session, showing significant effect of phase [*F*(1,11) = 10.236, *p* = 0.008, ${\text{η}}_{\text{p}}^{2}$ = 0.482 and *F*(1,10) = 7.903, *p* = 0.018, ${\text{η}}_{\text{p}}^{2}$ = 0.441 for WT and BACHD, respectively] and phase × duration interaction [*F*(3.234,35.578) = 3.047, *p* = 0.038, ${\text{η}}_{\text{p}}^{2}$ = 0.217 and *F*(6,60) = 2.699, *p* = 0.032, ${\text{η}}_{\text{p}}^{2}$ = 0.213 for WT and BACHD, respectively]. For Middle-Aged animals, WT showed no significant long-term disruption (WT: *p*s > 0.337), while BACHD showed a slight disruption that did not reach significance [phase: *F*(1,11) = 3.248, *p* = 0.099; phase × duration: *F*(3.481,38.293) = 2.331, *p* = 0.081].

When fitting 24 h post-stress individual bisection curves, *R*^2^ were all greater than 0.900, except for one middle-aged BACHD rat for which *R*^2^ was lower (0.817) but with an acceptable fit. Mixed ANOVA with age and genotype as group factors and phase (baseline vs. 24 h post) as repeated measure showed a significant decrease in PSE [**Figure 4A**, 3.73 ± 0.07 vs. 4.06 ± 0.07; *F*(1,41) = 19.583, *p* < 0.001, ${\text{η}}_{\text{p}}^{2}$ = 0.323], with only a trend for phase × age interaction [*F*(1,41) = 3.134, *p* = 0.084]. As for gamma (**Figure 4B**), no significant long-term effect was observed (no significant phase or interaction involving phase, all *p*s > 0.147).

In sum, the long-term effects of the mild stress were mainly visible on p(long) functions, and when significant, were related to age rather than genotype. However, we cannot preclude the possibility that the effects, when observed, may have not reflected a long-term effect of stress, but rather session repetition, in absence of non-shock control groups. Nevertheless, it is clear that the strong acute effects of foot-shocks observed in wild-type animals, with a disruption of behavior as well as temporal discrimination, were no longer seen at long-term.

### Peak Interval

One old BACHD rat died during the task and was thus discarded from the analysis of this task.

#### Acquisition

**FIGURE 5:**
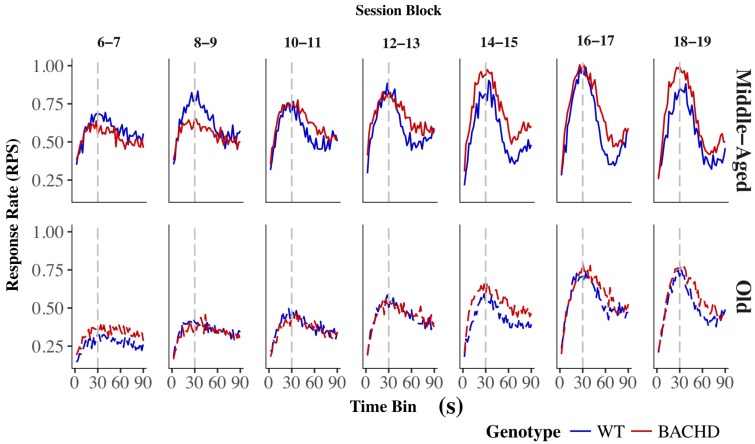
Group mean lever presses per second as a function of elapsed trial time on PI trials for wild-type (blue line) and BACHD (red line) rats. The top and bottom rows depict data from middle-aged and old rats, respectively, on each of the 7 two-session blocks (in columns). Response rates are plotted in 2-s bins. The trained FI criterion duration is denoted by a vertical gray dashed line at 30 s.

**FIGURE 6:**
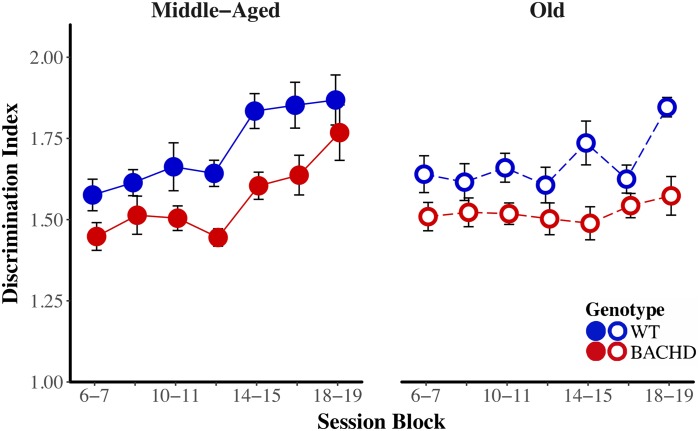
Temporal discrimination acquisition during the PI training phase. Group mean (±SEM) temporal discrimination index across session blocks for middle-aged **(left)** and old **(right)** rats for each genotype.

The average response rates on probe trials as a function of elapsed time for both genotypes and ages are plotted in **Figure 5**. Over blocks of training sessions, the maximum response rate appeared to increase across genotypes and ages. While older rats appeared to have lower response rates than Middle-Aged rats, there did not appear to be systematic differences in rates between genotypes. To assess the development of temporal control over training, we calculated for each rat and session block an index of temporal discrimination by dividing the maximum response rate by the mean response rate. As shown in **Figure 6**, temporal discrimination appeared to increase over blocks of training, with wild-type animals showing consistently better discrimination than BACHD animals. A mixed ANOVA with genotype, age, and block as factors confirmed that BACHD animals had poorer temporal discrimination than wild-type animals [*F*(1,42) = 23.10, *p* < 0.001, ${\text{η}}_{\text{p}}^{2}$ = 0.355]. The analysis also revealed a significant effect of block [*F*(4.49,188.65) = 12.37, *p* < 0.001, ${\text{η}}_{\text{p}}^{2}$ = 0.228] and age × block interaction [*F*(4.49,188.65) = 3.19, *p* = 0.011, ${\text{η}}_{\text{p}}^{2}$ = 0.071]. No other effects or interactions reached significance for the discrimination index (all *p*s > 0.05). Thus, although all groups showed a progressive acquisition of peaked response rates, Middle-Aged rats showed a faster increase in maximum response rate as compared to the global increase in the average rate of lever pressing, indicating a steeper acquisition of temporal discrimination than old rats.

To quantify differences in response rates across genotypes, ages, and blocks of training, we examined rates of responding from the individual-trial analysis. The proportion of trials with a low–high–low pattern was analyzed to assess differences across ages, genotypes, and blocks of training. A mixed ANOVA with these factors revealed only a significant effect of session block on the proportion of these trials [*F*(6,252) = 2.87, *p* = 0.010, ${\text{η}}_{\text{p}}^{2}$ = 0.064], with a mean proportion increasing from 0.695 (±0.016) on Session Block 6–7 to 0.758 (±0.018) on Session Block 18–19. No other effects reached significance (all *p*s > 0.05).

**Table 1** presents grand means for all parameters extracted from the individual-trial analysis. Rates of responding in the initial low-rate state (*r*_1_) decreased over blocks of training, *F*(4.46,187.37) = 3.36, *p* = 0.003, ${\text{η}}_{\text{p}}^{2}$ = 0.074, while rates of responding in the high-rate state (*r*_2_) increased with training, *F*(2.92,404) = 5.97, *p* < 0.001, ${\text{η}}_{\text{p}}^{2}$ = 0.124. Old rats showed lower *r*_1_and *r*_2_than Middle-Aged rats, *F*(1,42) = 6.35, *p* = 0.016, ${\text{η}}_{\text{p}}^{2}$ = 0.131, and *F*(1,42) = 8.12, *p* = 0.007, ${\text{η}}_{\text{p}}^{2}$ = 0.162, respectively. Furthermore, there was a significant interaction between age and block on *r*_3_, *F*(2.85,119.70) = 11.42, *p* < 0.001, ${\text{η}}_{\text{p}}^{2}$ = 0.214, such that Middle-Aged rats had higher response rates than Old rats on the first three blocks of training (all *p* < 0.05); these differences in *r*_3_ between ages diminished for the remaining blocks of training (all *p*s > 0.05). None of the other main effects or interactions reached significance for the measures of *r*_1_, *r*_2_, and *r*_3_.

**Table 1:** Grand means for individual trial statistics during the PI training phase.

*r*_1_	*r*_2_	*r*_3_	*r*_1/_*r*_2_	*r*_3/_*r*_2_	Start	Stop	Spread	IQR_start_	IQR_stop_	IQR_spread_
14.05	71.74	21.30	0.19	0.28	25.56	47.18	13.78	26.15	29.91	16.95

**FIGURE 7:**
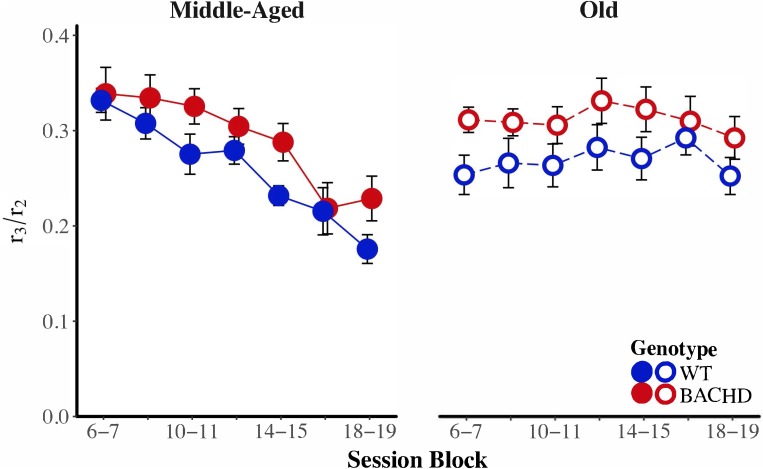
Group mean ratios of response rate in the second low-rate state (*r*_3_) over response rate in the high-rate state (*r*_2_) for middle-aged **(left)** and old **(right)** rats across session blocks for each genotype. Error bars depict the SEM.

BACHD subjects appeared to be poorer than wild-type subjects at transitioning from a high rate of responding to a low rate of responding. We calculated a response initiation ratio (*r*_1/_*r*_2_) and a response suppression ratio (*r*_3/_*r*_2_) to quantify changes in response rates across states. A ratio of 1 indicates a lack of response differentiation between the low-rate and high-rate states, while a ratio of 0 indicates perfect differentiation of responding between the two states. These ratios were subject to a mixed ANOVA with genotype, age, and block as factors. The response initiation ratio decreased as a function of training, *F*(6,252) = 13.26, *p* < 0.001, ${\text{η}}_{\text{p}}^{2}$ = 0.240, but there were no effects or interactions with the factors of genotype and age for this measure. **Figure 7** depicts the response suppression ratio as a function of session block for both genotypes and ages. A mixed ANOVA revealed a significant interaction between age and block on the response suppression ratio, *F*(3.58,150.29) = 9.82, *p* < 0.001, ${\text{η}}_{\text{p}}^{2}$ = 0.190, such that during the first block of training, Middle-Aged animals had higher ratios than Old animals, *F*(1,44) = 7.29, *p* = 0.010, ${\text{η}}_{\text{p}}^{2}$ = 0.142, while on the penultimate and last session blocks, Old animals had higher response suppression ratios than the Middle-Aged animals, *F*(1,44) = 12.50, *p* < 0.001, ${\text{η}}_{\text{p}}^{2}$ = 0.221, and *F*(1,44) = 10.60, *p* = 0.002, ${\text{η}}_{\text{p}}^{2}$ = 0.194, respectively. This pattern of results along with the data in **Figure 7** suggest that while response suppression ratios decreased over training for Middle-Aged animals, they remained unchanged for Old animals. With respect to differences between genotypes, the mixed ANOVA revealed that WT subjects had significantly lower response suppression ratios than BACHD subjects, *F*(1,42) = 6.86, *p* = 0.012, ${\text{η}}_{\text{p}}^{2}$ = 0.140, suggesting that BACHD subjects had greater difficulty inhibiting responding.

To assess the development of temporal control over training, we subjected the median start, stop, and spread times, as well as the interquartile range (IQR) of start and stop times to mixed age × genotype × block ANOVAs. As their first and second-order interactions between age and genotype did not reach significance for any of the measures, effects involving age and genotype will be addressed sequentially. Non-specific to age or genotype, a main effect of block on spread revealed that spread times increased with repeated training, *F*(4.43,186.12) = 25.16, *p* < 0.001, ${\text{η}}_{\text{p}}^{2}$ = 0.375. Furthermore, the variability of start times decreased with repeated training, *F*(5.29,222.28) = 14.22, *p* < 0.001, ${\text{η}}_{\text{p}}^{2}$ = 0.253.

**FIGURE 8:**
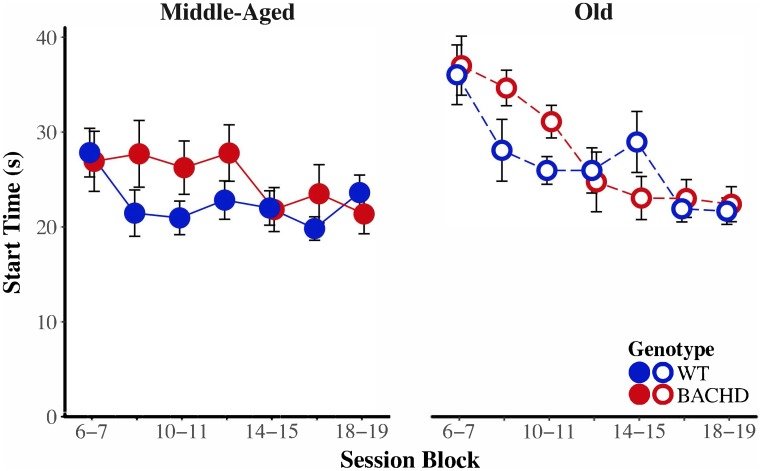
Group mean start times (s) for middle-aged **(left)** and old **(right)** rats across session blocks for each genotype. Error bars depict the SEM.

**FIGURE 9:**
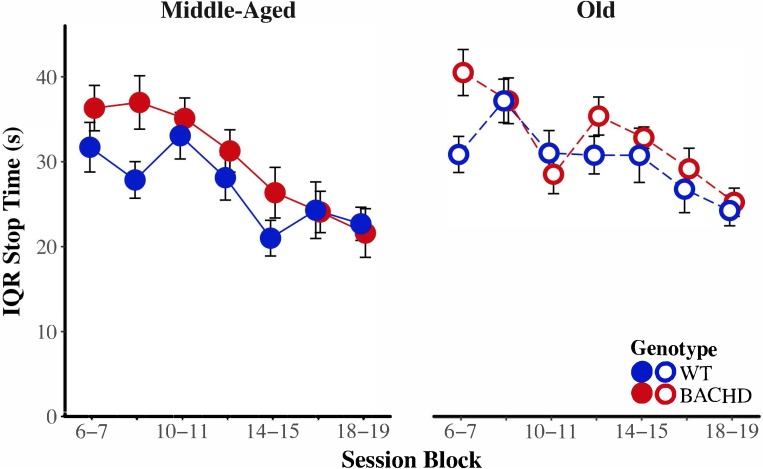
Group mean interquartile ranges of stop time for middle-aged **(left)** and old **(right)** rats across session blocks for each genotype. Error bars depict the SEM.

**Figure 8** depicts start time as a function of training blocks across both genotypes and ages. With respect to age-related differences in temporal control, there was a significant interaction between age and session block on start times, *F*(5.58,234.37) = 4.04, *p* < 0.001, ${\text{η}}_{\text{p}}^{2}$ = 0.088. Parsing the interaction between age and block on start times revealed that old animals had significantly later start times than Middle-Aged animals on the first three blocks of testing (all *p* < 0.037). These differences between ages diminished with training, such that Middle-Aged and Old animals did not differ on the last four blocks of testing (all *p*s > 0.05). In addition, there was greater variability in start times, as measured by the IQR, for Old rats than for Middle-Aged rats, *F*(1,42) = 6.15, *p* = 0.017, ${\text{η}}_{\text{p}}^{2}$ = 0.128. **Figure 9** depicts the variability in stop time as a function of training blocks across both genotypes and ages. There was a significant interaction between block and age on the variability of stop times, *F*(5.62,236.05) = 2.53 *p* = 0.025, ${\text{η}}_{\text{p}}^{2}$ = 0.057, such that on the fifth session block, Old rats showed greater variability in stop times than Middle-Aged rats, *F*(1,44) = 9.44, *p* = 0.004, ${\text{η}}_{\text{p}}^{2}$ = 0.177; the variability in stop times did not differ across ages for any other session block (all *p*s > 0.05). Spread time was significantly longer for Middle-aged rats than Old rats, *F*(1,42) = 5.91, *p* = 0.019, ${\text{η}}_{\text{p}}^{2}$ = 0.123.

BACHD animals showed several impairments in temporal control relative to wild-type counterparts. There was a significant interaction between block and genotype on start time (**Figure 8**), *F*(5.58,234.37) = 3.35, *p* = 0.004, ${\text{η}}_{\text{p}}^{2}$ = 0.074, such that on the third block of training, BACHD animals had significantly later start times than WT animals, *F*(1,44) = 5.52, *p* = 0.023, ${\text{η}}_{\text{p}}^{2}$ = 0.111. BACHD animals had significantly later and more variable stop times than WT animals (**Figure 9**), *F*(1,42) = 7.14, *p* = 0.011, ${\text{η}}_{\text{p}}^{2}$ = 0.145, and *F*(1,42) = 4.74, *p* = 0.035, ${\text{η}}_{\text{p}}^{2}$ = 0.101, respectively. In summary, the effects of HD were primarily on response termination—as reflected in between-group differences in the response suppression ratio, stop times, and the variability of stop times—and did not disappear with extended training.

We assessed whether the correlations between start time and stop time as well as between start time and spread time were in the same direction as reported in prior studies (Gibbon and Church, 1990; Church et al., 1994). Pearson product-moment correlations were calculated for each animal based on performance on PI trials from the last two-session block of training, using the same constraints as in Church et al., i.e., trials for which starts and stops bracketed the FI value. **Table 2** contains start-stop and start-spread correlations for all four combinations of age and genotype; for each group, there was a negative correlation between start time and spread time (significant one-sample *t*-tests against 0 for each of the four groups, all *p*s < 0.001), in line with previous reports. However, we failed to find a significant positive correlation between start and stop times for each of the four groups (all *p*s > 0.05). Failure to find significant start-stop correlations may reflect smaller variance in the remembered time of reinforcement relative to variance in the threshold of similarity.

**Table 2:** Group mean (±SEM) start-stop and start-spread pearson product-moment correlations.

Age	Genotype	Start-stop	Start-spread
Old	BACHD	-0.081 (0.121)	-0.456 (0.085)
Old	WT	-0.061 (0.081)	-0.442 (0.059)
Middle-Aged	BACHD	-0.132 (0.100)	-0.539 (0.070)
Middle-Aged	WT	0.030 (0.111)	-0.404 (0.080)

Analysis of the mean peak interval function on the last block of training (**Figure 5**, last upper and lower panels) revealed a significantly greater width for BACHD than WT groups [*F*(1,42) = 7.870, *p* = 0.008, ${\text{η}}_{\text{p}}^{2}$ = 0.158], and for Old than for Middle-Aged animals [*F*(1,42) = 4.746, *p* = 0.035, ${\text{η}}_{\text{p}}^{2}$ = 0.102], with a trend toward an interaction [*F*(1,42) = 3.430, *p* = 0.071]. Based on our *a priori* hypothesis, the analysis of the genotype effect at each age showed a significant effect only for the Old animals [*F*(1,20) = 6.985, *p* = 0.016, ${\text{η}}_{\text{p}}^{2}$ = 0.259 and *F*(1,22) < 1 for Old and Middle-Aged rats, respectively]. Width represents twice the standard deviation of the Gaussian fit, and larger values reflect less timing precision. Mean widths were 41.38 s and 54.72 s, for Middle-Aged and Old BACHD, respectively, and 38.23 s and 39.31 s for Middle-Aged and Old WT, respectively. For peak time, peak rate and coefficient of variation, there were no significant effects (see **Table 3**, all *p*s > 0.05).

**Table 3:** Group mean (±SEM) peak time, peak rate, width and coefficient of variation during the last block of training.

Age	Genotype	Peak time	Peak rate	Width	CV
Old	BACHD	24.476 (1.716)	0.816 (0.115)	54.719 (4.761)	2.572 (0.558)
Old	WT	24.581 (1.399)	0.637 (0.082)	39.311 (3.555)	1.764 (0.294)
Middle-aged	BACHD	26.849 (1.661)	1.007 (0.153)	41.385 (2.720)	1.635 (0.165)
Middle-aged	WT	27.122 (1.902)	0.787 (0.099)	38.231 (2.076)	1.556 (0.215)

#### Effect of Stress

##### Acute effect

**FIGURE 10:**
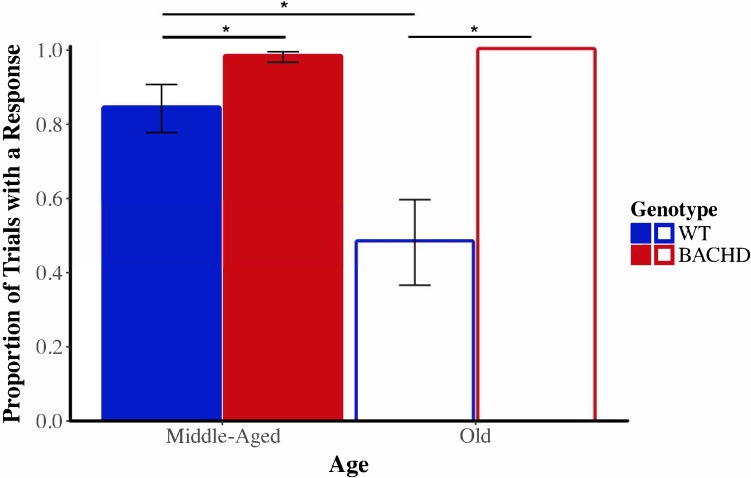
Group mean proportion of PI trials with at least one lever press response during the shock test (Session 20) across ages and genotypes. Error bars depict the SEM. Asterisks denote significant differences between groups (*p* < 0.05).

Before evaluating the effects of shock on timing in the peak interval procedure, we began by calculating the proportion of probe trials in which at least one lever-press response was emitted for each subject on the last session of training prior to the shock test, during the shock test, and on the session following the shock test. **Figure 10** depicts the mean proportion of probe trials with at least one operant response during the shock test across genotypes and ages. Visual inspection of these data suggested that responding of Old WT animals was especially disrupted by the shock. The proportion of trials with a response during the shock session ranged from 0.06 to 1.00 across subjects for Old WT animals and from 0.39 to 1.00 for Middle-Aged WT animals. In contrast, BACHD animals responded on the majority of trials, with the proportion of trials with a response being 1.00 for all Old BACHD rats and ranging from 0.83 to 1.00 for Middle-Aged BACHD ones. A mixed ANOVA conducted with between-subjects factors of genotype and age and a repeated-measures factor of session (24 h pre-shock, shock, 24 h post-shock) revealed a significant age × genotype × session interaction on the mean proportion of trials with a response, *F*(1.02,42.93) = 7.36, *p* = 0.009, ${\text{η}}_{\text{p}}^{2}$ = 0.149. Parsing the interaction by session revealed a significant age × genotype interaction on the shock test session, *F*(1,42) = 7.40, *p* = 0.009, ${\text{η}}_{\text{p}}^{2}$ = 0.150, but not on the pre-shock or post-shock sessions (all *p* > 0.05). On neither the pre- nor post-shock session were there any significant effects of genotype or age or interactions between the two factors (all *p*s > 0.05). On the shock session, Old WT rats responded on fewer probe trials than did Middle-Aged WT rats, *t*(22) = 2.73, *p* = 0.012; Old BACHD rats did not differ from Middle-Aged BACHD rats, *t*(20) = 1.18, *p* = 0.251. Furthermore, Old WT rats responded on fewer trials than Old BACHD rats, *t*(20) = 4.09, *p* = 0.001; likewise, Middle-Aged WT rats responded on fewer trials than Middle-Aged BACHD rats, *t*(22) = 2.09, *p* = 0.048. Given the differences between groups on the proportion of trials with a response during the shock test between genotypes and ages, we did not proceed with between-group comparisons of temporal control measures during the shock session.

##### Long-term effect

As there were no differences in the proportion of trials with a response between genotypes or ages on the last session of training or on the session following the shock test, we evaluated if the shocks induced enduring changes in temporal control. Start, stop, and spread times were analyzed with a mixed ANOVA with the between-subjects factors of genotype and age and the within-subjects factor of session (24 h pre-shock vs. 24 h post-shock). Start and stop times were significantly later during the post-shock session, *F*(1,42) = 4.58, *p* = 0.038, ${\text{η}}_{\text{p}}^{2}$ = 0.098, and *F*(1,42) = 7.83, *p* = 0.008, ${\text{η}}_{\text{p}}^{2}$ = 0.157, respectively. No other effects reached significance for start, stop, or spread times. While the acute effects of shock on general responding were differential across ages and genotype, there was no evidence that long-term effects on general responding or temporally controlled responding differed across groups.

### Sensitivity to Foot-Shock

**FIGURE 11:**
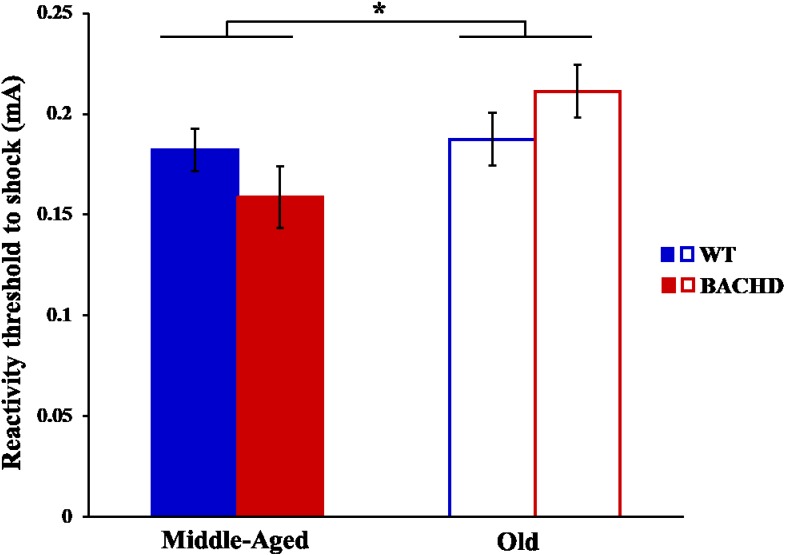
Reactivity threshold to electric foot-shocks in middle-aged and old rats. Error bars depict the SEM. Asterisks denote significant differences between groups (*p* < 0.05).

The reactivity thresholds to electric foot-shocks increased slightly with age [**Figure 11**, *F*(1,29) = 4.94, *p* = 0.034, ${\text{η}}_{\text{p}}^{2}$ = 0.146], with no difference between genotype and no age × genotype interaction (*p*s > 0.05).

## Discussion

In the present study, BACHD rats were assessed for their timing performances, both in normal condition and under a mild stress, and at two ages (4–6 months and 10–12 months), likely targeting different levels of neurodegeneration. Results from both temporal discrimination and peak interval timing tasks converge to the observation of some deficits in BACHD rats, mainly in older animals: (1) slower learning of the temporal discrimination task; and (2) retardation of acquisition of temporal control with a retardation in start times to reach asymptotic values, wider peak interval functions and later and more variable stop times, with a difficulty transitioning from high rate of responding to low rate of responding. Strikingly, performance of BACHD rats was undisrupted by mild stress, in strong contrast with the age-related profound disruption of both responding and timing (i.e., poorer precision) in wild-type animals.

Beside the effect of genotype, effects of age were also apparent across the two tasks. In the bisection procedure, gamma was higher for older rats than middle-aged rats, indicating age-related declines in temporal sensitivity. Lustig and Meck (2001) reported that older adults showed poorer sensitivity to time than younger adults, although it may depend on the training condition and the modality of the timing cue. Within the PI procedure of the present study, older animals showed lower operant response rates than middle-aged animals, replicating previous investigations of age-related changes in timing in the PI procedure in rats (Lejeune et al., 1998; Church et al., 2014). We failed to find differences in start or stop times between ages, in contrast to the typical findings of later start, stop, and peak times for older animals (Lejeune et al., 1998; Meck, 2006). Failure to replicate this age effect may be attributable to absolute or the relative age discrepancy between groups. Groups in previous studies by Lejeune et al. (1998) and Meck (2006) differed by 20 months, with a relative age discrepancy of 400–600% between groups; in the present study, groups differed by 5 months, with a relative age discrepancy of 220%. In a novel finding, we found that older rats had smaller spread times than young rats. Traditionally, a decrease in spread time has been interpreted as an increase in sensitivity to time—an interpretation that runs counter to age-related increases in gamma, which is reflective of a decrease in sensitivity to time. However, spread time may also be sensitive to changes in multiple processes including motivation (Galtress and Kirkpatrick, 2009), in addition to changes in the perception of time. As such, apparent discrepancies in age-related changes to gamma and spread time may reflect protocol-specific effects. Overall, our findings extend the existing body of literature on age-related changes in interval timing (Xu and Church, 2017).

The BACHD rat model for HD showed different types of disruption, some of which may be related to a modified temporal processing, while others may reflect other factors. The higher terminal level of responding, later stop times, increased variability in stop times, and greater width in the peak interval task are similar to the effects reported in HD mice models showing increased width (variability) in the peak interval function and later stop times than in wild-types (Balci et al., 2009). It is also consistent with a recent report in HD patients who exhibited decreased precision (increased width) in timing in an analogous type of task (Agostino et al., 2017). The observed difficulty in stopping responding in the rodent studies may be related to difficulty in inhibitory control, once the animal has started responding, rather than a reflection of a timing deficit *per se*. The fact that similar findings were found in both animals and humans, and that scalar property (precision inversely proportional to the duration timed; Gibbon, 1977) was found to be violated in the HD patients (Rao et al., 2014; Agostino et al., 2017), may suggest, on the other hand, a timing deficit.

A timing deficit should, however, be observed also in other temporal tasks. The results obtained in the bisection tests did not show any sign of a deficit, in any of the dependent measures. While previous data showed greater variability (gamma) in ∼6 months old tgHD rats than wild-types (Höhn et al., 2011), only a trend was observed in the present experiment. It may be due to different HD models (BACHD vs. tgHD) or difference in procedures, but it may reflect a different level of degeneration, although the increased response latencies during the 2 vs. 8 s discrimination training attest to some neurodegenerative status in the old BACHD animals in the present study. In fact, a study in HD patients has shown that deficits in temporal production were observed earlier than those in temporal estimation in the course of the disease (Beste et al., 2007). As the deficit in the peak interval task was observed to emerge with age, reaching significance in old BACHD animals only, it may suggest that the degeneration status was not advanced enough to be reflected in the bisection task, an estimation procedure.

Retardation in 2 vs. 8 s temporal discrimination learning in conjunction with a delay in reaching asymptotic start times levels in the peak interval task may indicate specific deficits related to time. These data join others in demonstrating poorer acquisition for HD subjects in time estimation (Righi et al., 2016) and production (Balci et al., 2009) tasks. The intact bisection performance, however, indicates no timing deficit *per se* at that age, thus suggesting that the deficits may reflect difficulties in learning that may not be specific to time, as learning deficits in HD animals have been shown in other tasks (Adjeroud et al., 2015; El Massioui et al., 2016; Manfré et al., 2016; Clemensson et al., 2017).

Timing behavior was also not affected by unpredictable mild foot-shocks in BACHD rats, whatever their age, in striking contrasts with the large emotion-induced deficit seen in old WT animals in both bisection and peak interval tasks. In both tasks, emotion induced by foot-shocks disrupted ongoing behavior and precision in temporal discrimination in WT animals, with a stronger impact in older animals. Moreover, while the acute effects of shock on general responding were differential across ages and genotype, there was no clear evidence of long-term effects (24 h later) attributable to differential effects of stress depending on age or genotype on general responding or temporally controlled responding. These results confirm and strengthen our previous study reporting a reduced impact of shock-induced emotion on decision making in BACHD rats (Adjeroud et al., 2015). In this study, the effects of mild foot-shocks on performance in a gambling task were quite weak in WT animals, possibly due to the fact that shocks were presented offline, before the gambling session. However, as in the present study, the impact of foot-shocks was non-existent in BACHD rats. It seems thus that behavioral performance in executive tasks, interval timing processing and decision-making, are not impaired by emotion induced by electric foot-shock in BACHD rats, even though sensitivity to shocks was not altered in these animals. These results suggest a dysregulation of emotion processing and an absence of interference between emotion and cognition in this HD model.

Studies on emotion in animal models of HD remain sparse and have contradictory results according to the model (number of CAG repetitions) as well as the age and the test used. Most of them suggest a phenotype of hypo-anxiety (File et al., 1998; Bolivar et al., 2003; Von Hörsten et al., 2003; Nguyen et al., 2006; Bode et al., 2008; Yu-Taeger et al., 2012; Zeef et al., 2012; Urbach et al., 2014) and emotional blunting (Faure et al., 2011, 2013), whereas some others show hyper-anxiety like behavior in some tasks (Carter et al., 1999; Hickey et al., 2005; Menalled et al., 2009; Southwell et al., 2009; Kordasiewicz et al., 2012; Abada et al., 2013). However, emotional dysregulation is poorly studied in HD even though these symptoms are often associated with the number of CAG repeats and the proximity of onset of motor symptoms (see Bora et al., 2016; Löffler et al., 2016; Kordsachia et al., 2017 for review). In HD patients, pathological emotional behaviors can include personality changes, with agitation and hyper-anxiety, but also irritability, possibly leading to aggressiveness (Litvan et al., 1998), often associated with depression and/or apathy (Paradiso et al., 2008). Altered recognition of emotional facial expressions is the most studied symptom evidenced by poor recognition of negative emotions as anger, disgust and fear (Sprengelmeyer et al., 1996; Paradiso et al., 2008; Snowden et al., 2008; Eddy et al., 2011; Novak et al., 2012; Rees et al., 2014) or of positive emotions (joy/happiness; Robotham et al., 2011; Henley et al., 2012) in premanifest as well as manifest HD patients.

Structural and functional changes in the brain, including frontal-subcortical emotion processing networks, have been associated with impaired subjective emotional experience in response to negative emotional pictures in HD (Paradiso et al., 2008; Ille et al., 2011; De Tommaso et al., 2013). Similarly, abnormalities in the neural networks underlying emotional processing and social cognition can be detected prior to clinical diagnosis in HD, including altered connectivity between the amygdala and other brain regions (Mason et al., 2015) and enhanced neural activation in limited regions, including the frontal lobes and amygdala in HD compared to controls (Novak et al., 2012; Dogan et al., 2014). In symptomatic tgHD rats, a reduced volume of the central nucleus of amygdala with an increased cellular activity has been reported (Faure et al., 2011; El Massioui et al., 2016). In BACHD rats, an increased neuronal reactivity (Arc labeling) to a threatening stimulus has been observed in the central amygdala of animals as young as 4.5 months old (Lamirault et al., 2017), suggesting an early impairment in neuronal networks involved in emotion processing.

In all, BACHD rats showed an age-dependent cognitive deficit with alterations of timing processing appearing mainly in 10–12 months old animals. In contrast, emotion dysregulation appeared from 4 to 6 months, i.e., before cognitive impairments. The results suggest a staggered onset in cognitive and emotional alterations, possibly related to different time courses of degeneration in cortico-striatal and amygdala circuits. This gives further arguments for emotional symptom as a potential powerful biomarker for disease onset in HD.

## Data Availability

The raw data supporting the conclusions of this manuscript will be made available by the authors, without undue reservation, to any qualified researcher.

## Author Contributions

VD, NM, BB, and DG elaborated the protocols, analyzed the results, and wrote the manuscript. CL participated in the data collection. HN and OR provided the transgenic animals and corrected the manuscript.

## Conflict of Interest Statement

The authors declare that the research was conducted in the absence of any commercial or financial relationships that could be construed as a potential conflict of interest.
