# Supplementary material for: The Alteration of Emotion Regulation Precedes the Deficits in Interval Timing in the BACHD Rat Model for Huntington Disease
Source: Front Integr Neurosci. 2018 May 9;12:14. doi: 10.3389/fnint.2018.00014 (PMC5954136; doi:10.3389/fnint.2018.00014)
Supplement: Supplementary file 1 [file Data_Sheet_1.PDF]

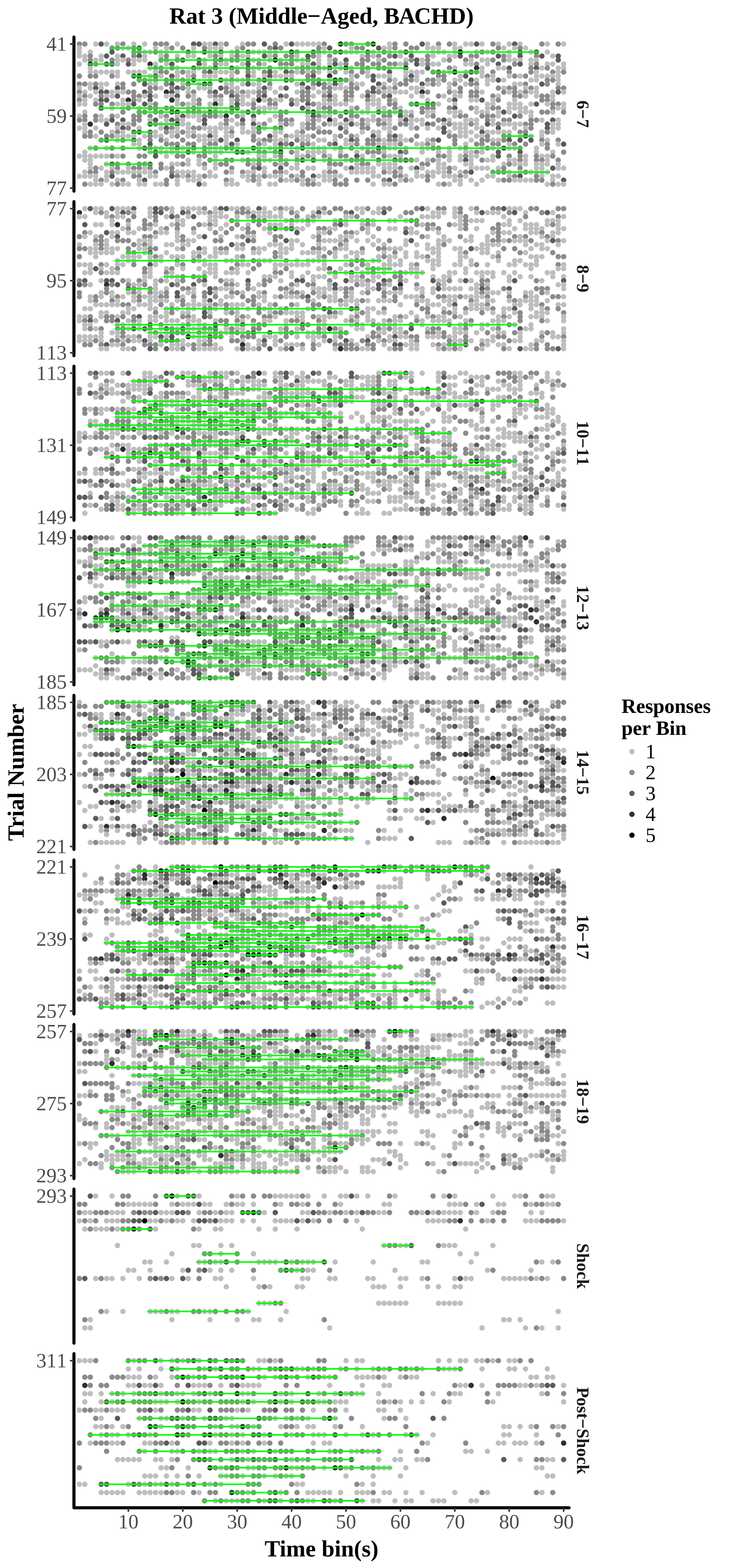

Rat 3 (Middle−Aged, BACHD)
Trial Number
Time bin(s)
Responses per Bin
1
2
3
4
5
6−7
8−9
10−11
12−13
14−15
16−17
18−19
Shock
Post−Shock
41
59
77
95
113
131
149
167
185
203
221
239
257
275
293
311
10
20
30
40
50
60
70
80
90

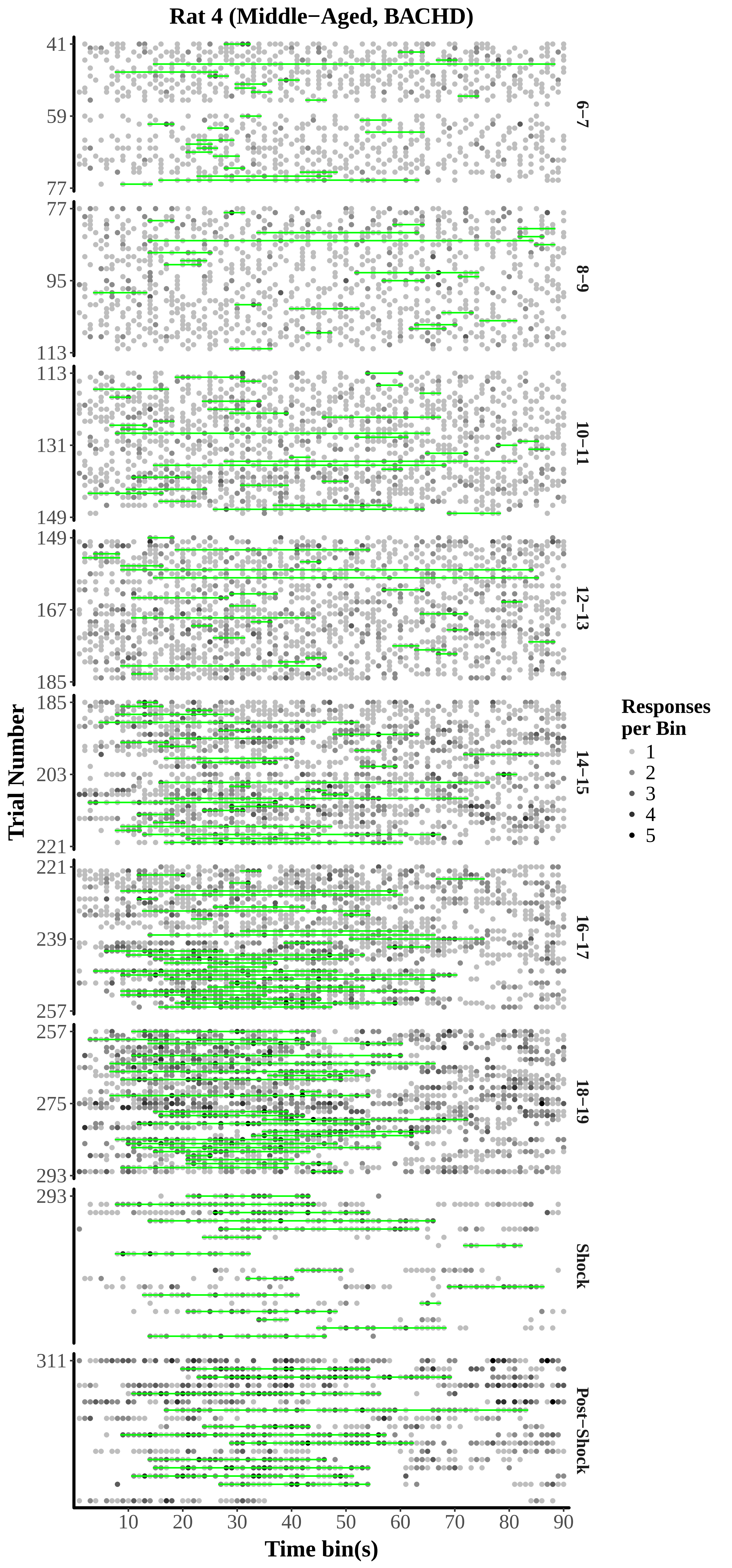

Rat 4 (Middle−Aged, BACHD)
Trial Number
Time bin(s)
Responses per Bin
1
2
3
4
5
6−7
8−9
10−11
12−13
14−15
16−17
18−19
Shock
Post−Shock
41
59
77
95
113
131
149
167
185
203
221
239
257
275
293
311
10
20
30
40
50
60
70
80
90

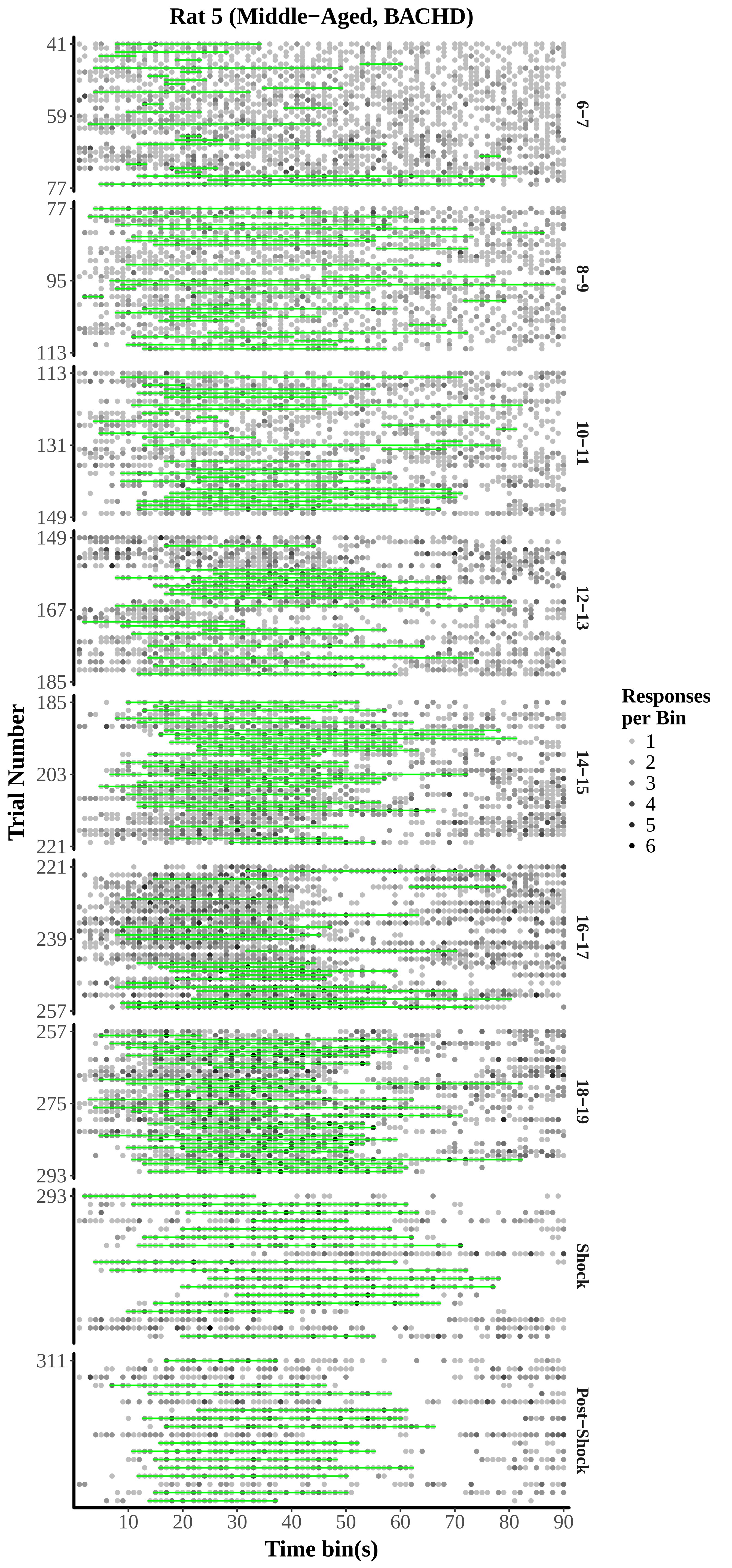
Rat 5 (Middle−Aged, BACHD)
Trial Number
Time bin(s)
Responses per Bin
1
2
3
4
5
6
6−7
8−9
10−11
12−13
14−15
16−17
18−19
Shock
Post−Shock
41
59
77
95
113
131
149
167
185
203
221
239
257
275
293
311
10
20
30
40
50
60
70
80
90

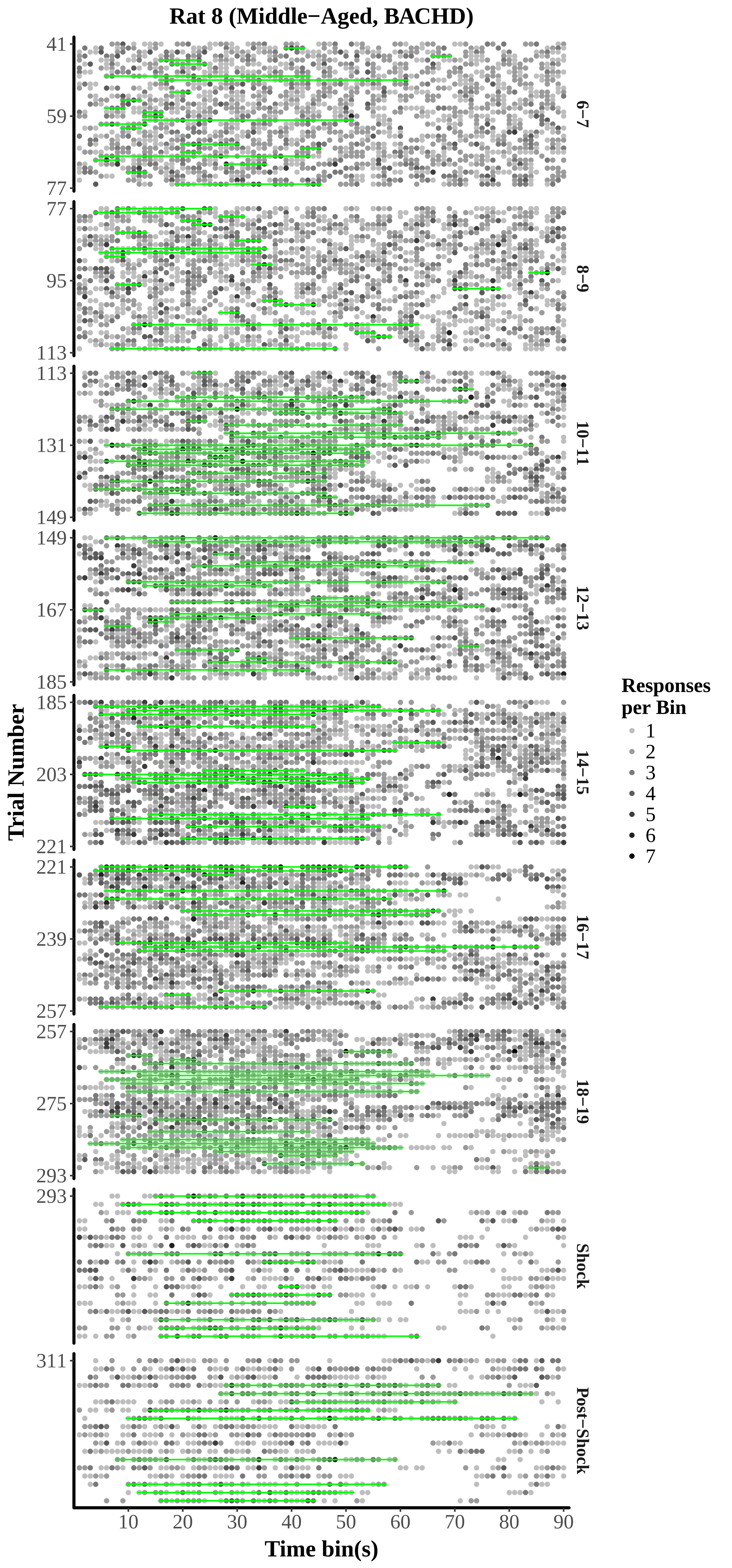

Rat 8 (Middle−Aged, BACHD)
Trial Number
Time bin(s)
6−7
8−9
10−11
12−13
14−15
16−17
18−19
Shock
Post−Shock
Responses per Bin
1
2
3
4
5
6
7

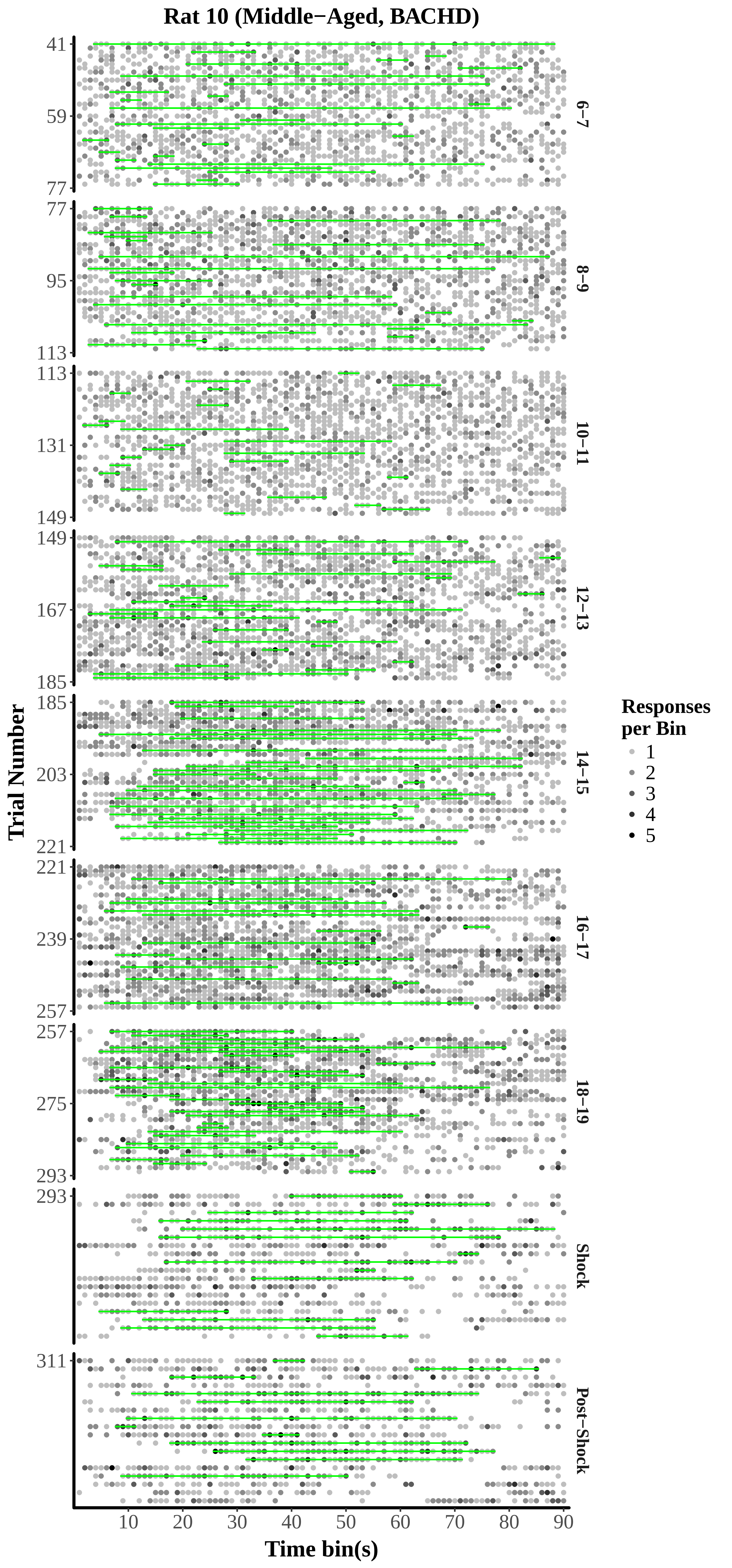

Rat 10 (Middle−Aged, BACHD)
Trial Number
Time bin(s)
Responses per Bin
1
2
3
4
5
6−7
8−9
10−11
12−13
14−15
16−17
18−19
Shock
Post−Shock
41
59
77
95
113
131
149
167
185
203
221
239
257
275
293
311
10
20
30
40
50
60
70
80
90

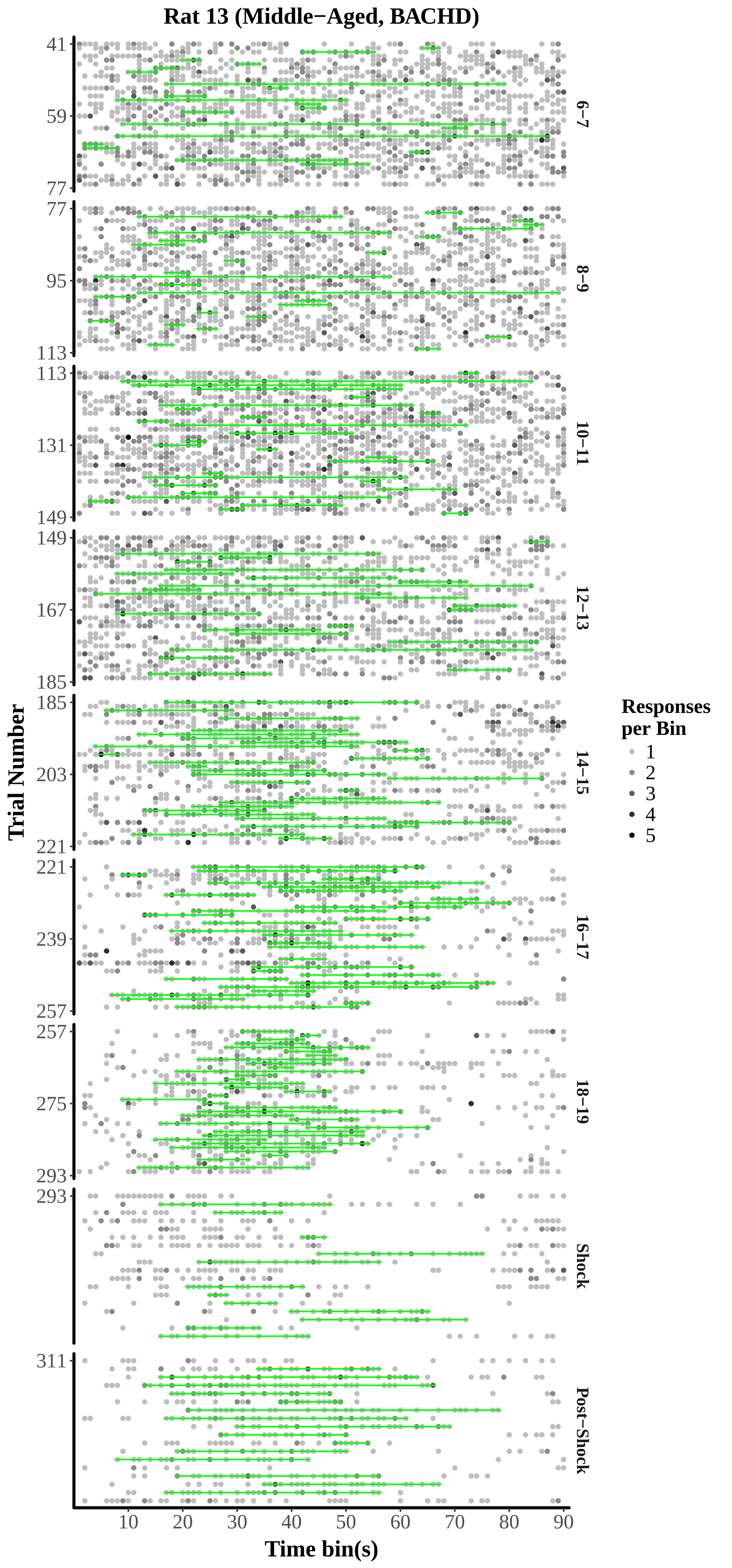

Rat 13 (Middle−Aged, BACHD)
Trial Number
Time bin(s)
41
59
77
95
113
131
149
167
185
203
221
239
257
275
293
311
10
20
30
40
50
60
70
80
90
6–7
8–9
10–11
12–13
14–15
16–17
18–19
Shock
Post−Shock
Responses per Bin
1
2
3
4
5

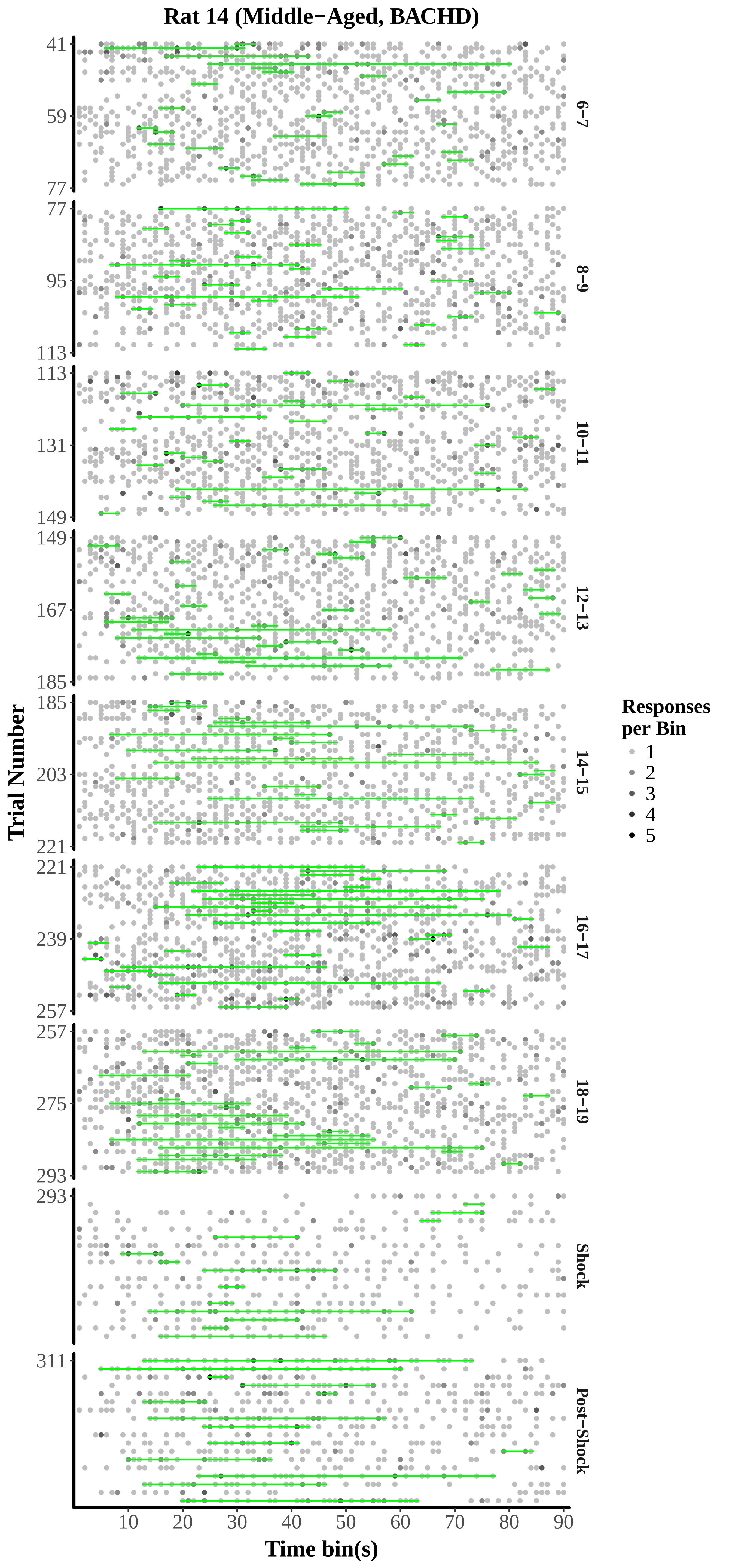
Rat 14 (Middle−Aged, BACHD)
Trial Number
Time bin(s)
41
59
77
95
113
131
149
167
185
203
221
239
257
275
293
311
10
20
30
40
50
60
70
80
90
6–7
8–9
10–11
12–13
14–15
16–17
18–19
Shock
Post−Shock
Responses per Bin
1
2
3
4
5

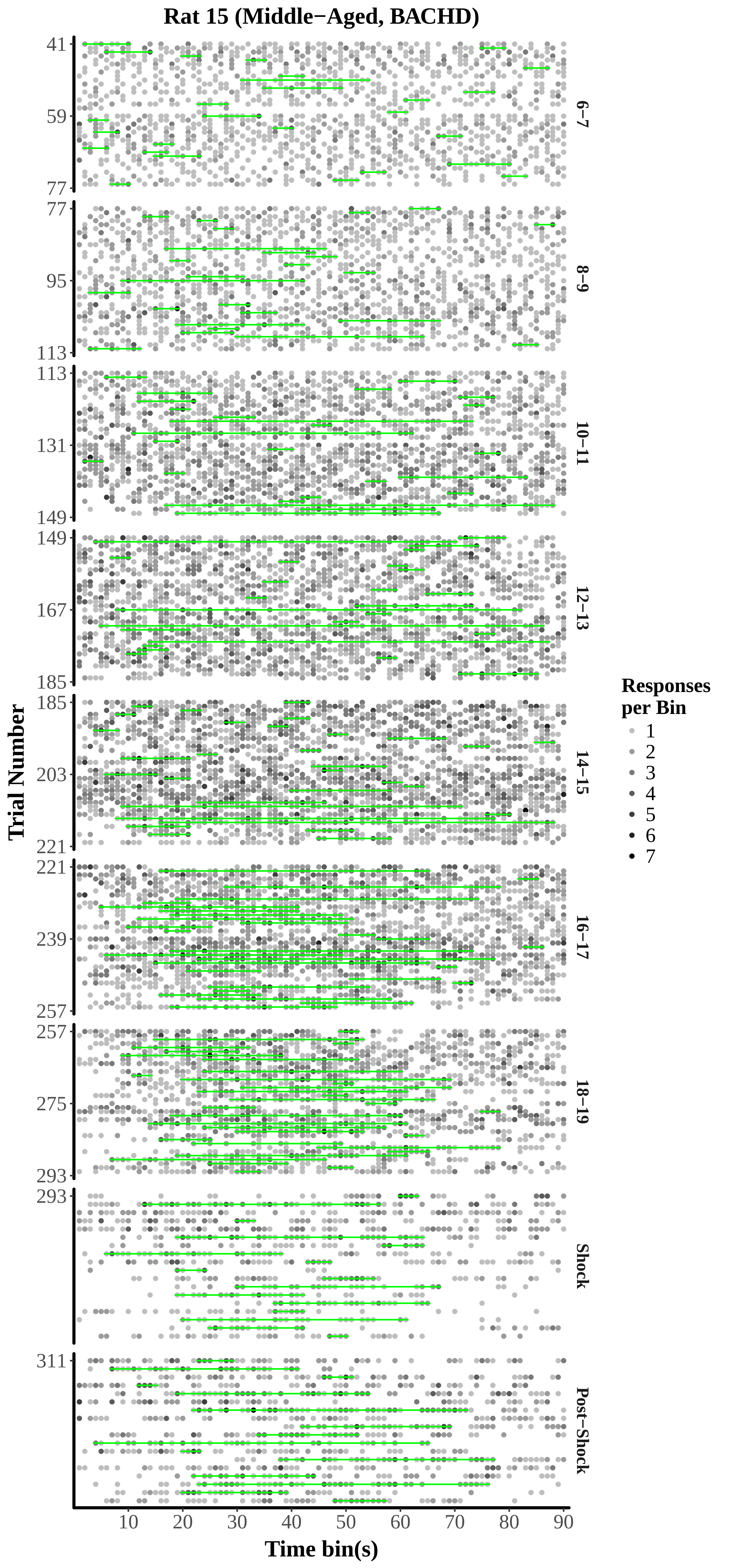

Rat 15 (Middle−Aged, BACHD)
Trial Number
Time bin(s)
6–7
8–9
10–11
12–13
14–15
16–17
18–19
Shock
Post−Shock
Responses per Bin
1
2
3
4
5
6
7
41
59
77
95
113
131
149
167
185
203
221
239
257
275
293
311
10
20
30
40
50
60
70
80
90

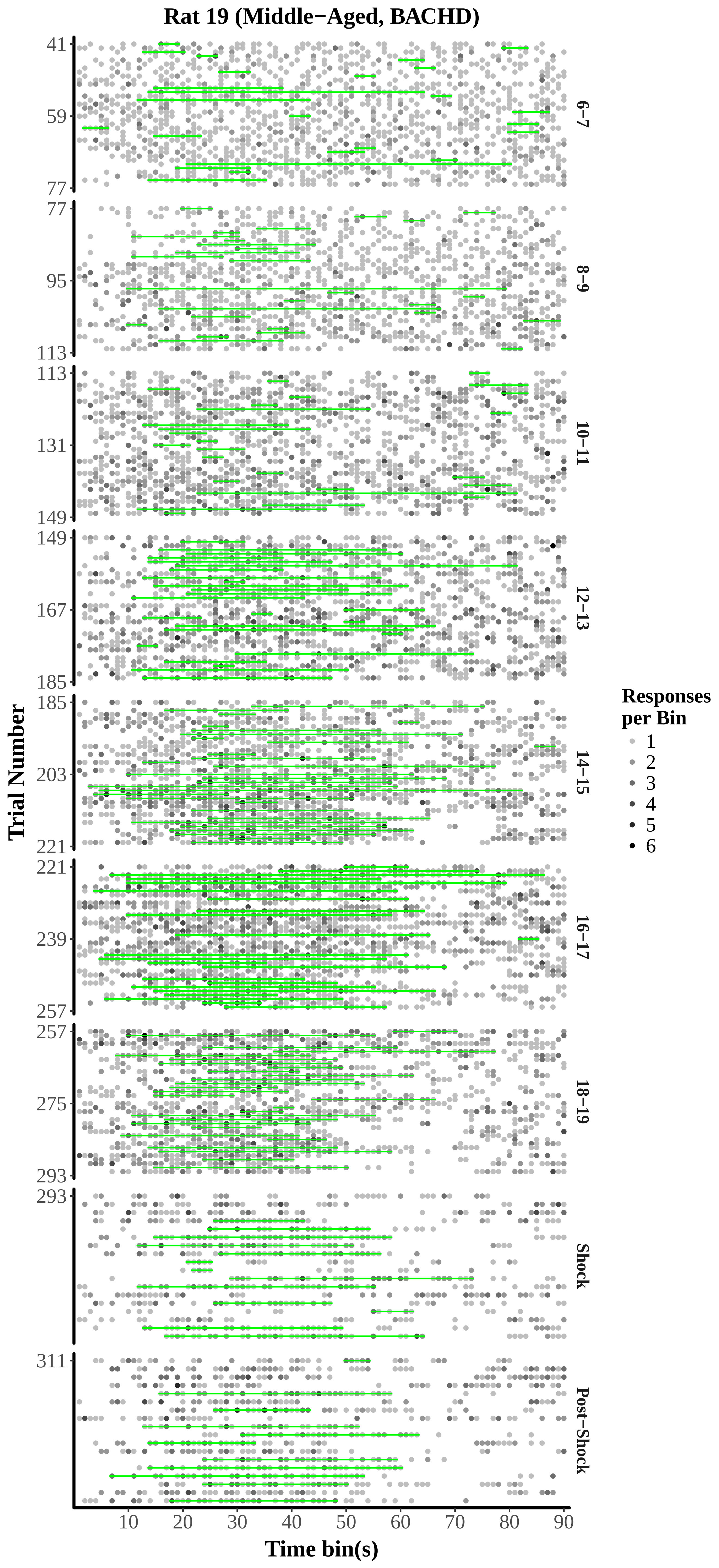

Rat 19 (Middle−Aged, BACHD)
Trial Number
Time bin(s)
Responses per Bin
1
2
3
4
5
6
6–7
8–9
10–11
12–13
14–15
16–17
18–19
Shock
Post−Shock
41
59
77
95
113
131
149
167
185
203
221
239
257
275
293
311
10
20
30
40
50
60
70
80
90

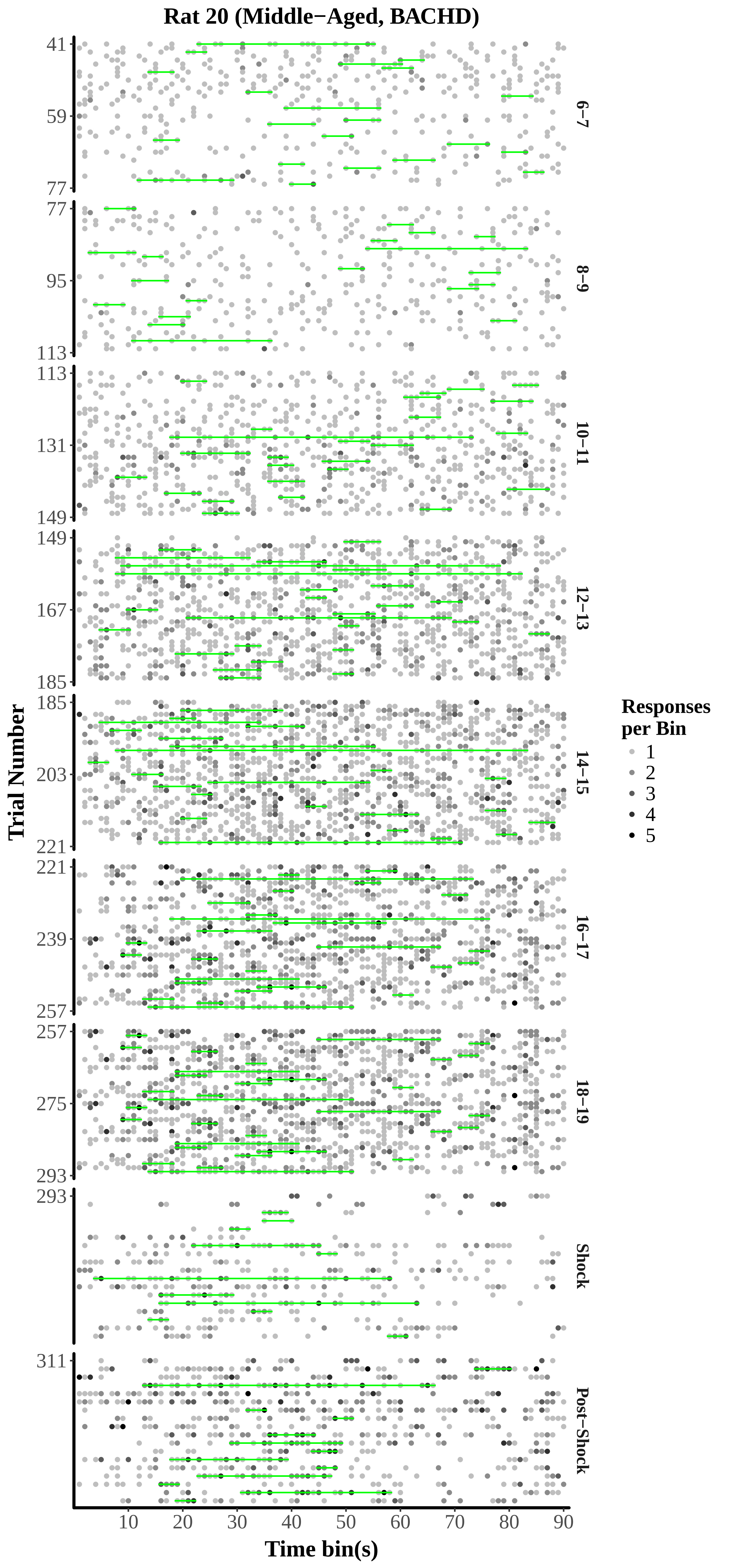
Rat 20 (Middle−Aged, BACHD)
Trial Number
Time bin(s)
6−7
8−9
10−11
12−13
14−15
16−17
18−19
Shock
Post−Shock
Responses per Bin
1
2
3
4
5

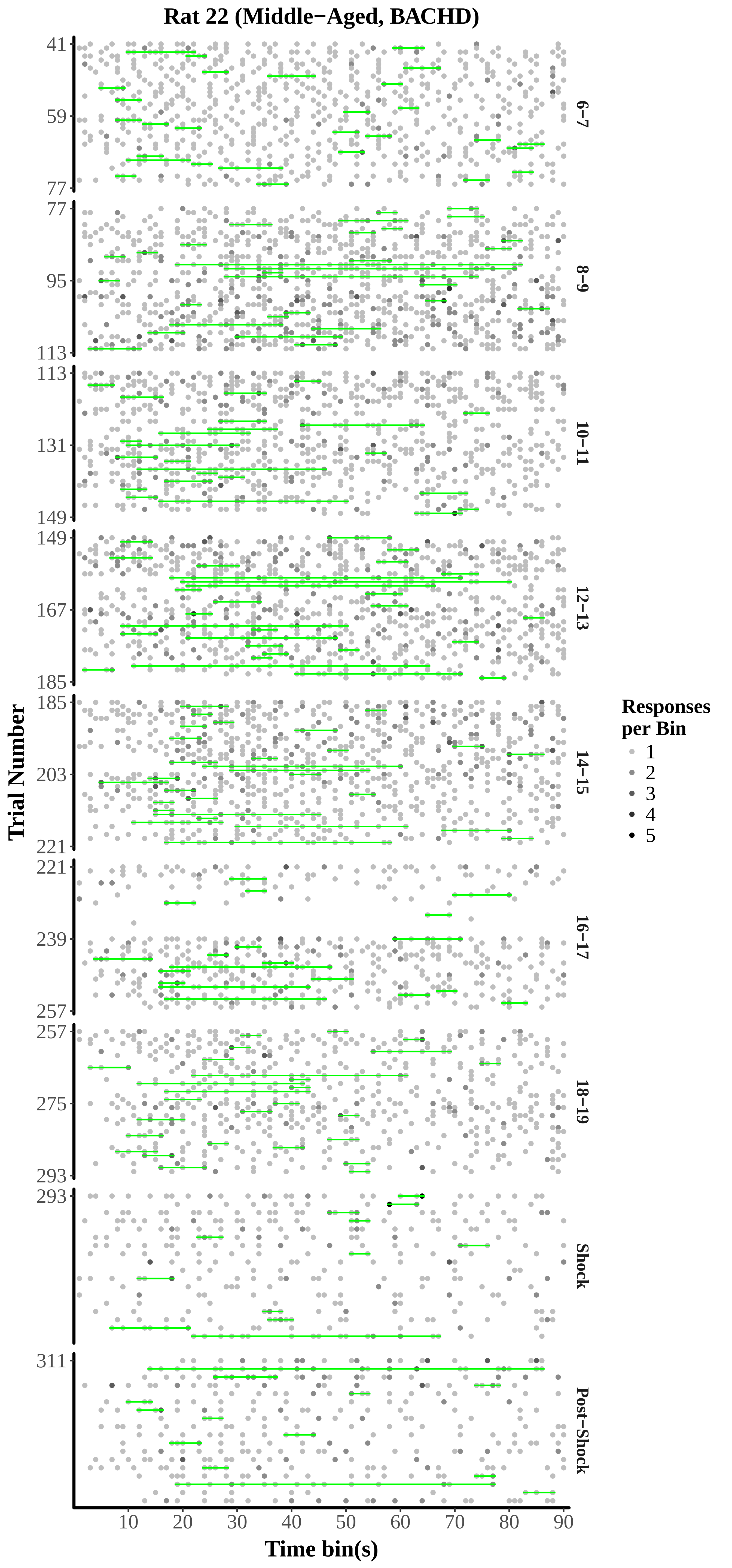
Rat 22 (Middle−Aged, BACHD)
Trial Number
Time bin(s)
10
20
30
40
50
60
70
80
90
41
59
77
95
113
131
149
167
185
203
221
239
257
275
293
311
6−7
8−9
10−11
12−13
14−15
16−17
18−19
Shock
Post−Shock
Responses per Bin
1
2
3
4
5

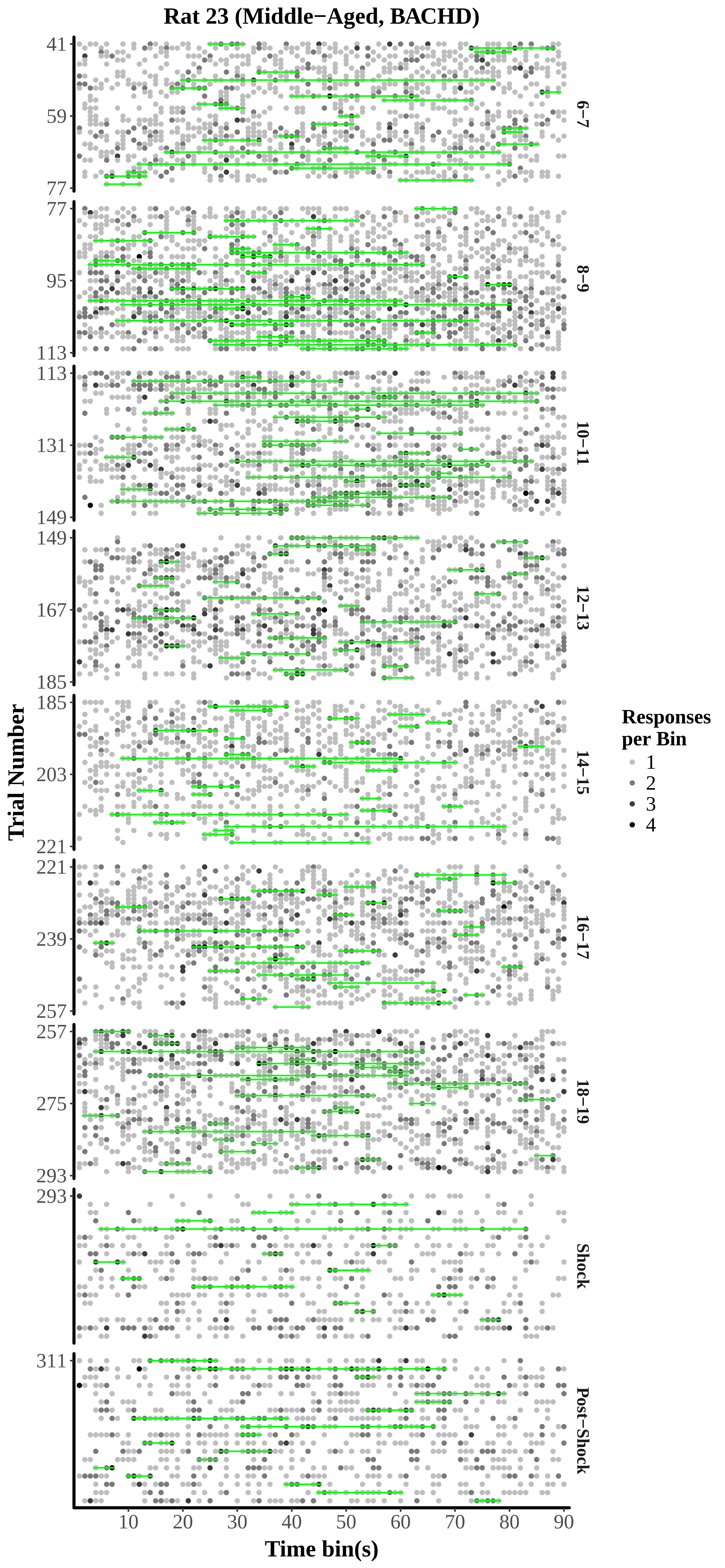

Rat 23 (Middle−Aged, BACHD)
Trial Number
Time bin(s)
10
20
30
40
50
60
70
80
90
41
59
77
95
113
131
149
167
185
203
221
239
257
275
293
311
6–7
8–9
10–11
12–13
14–15
16–17
18–19
Shock
Post−Shock
Responses per Bin
1
2
3
4
