# Supplementary material for: The Alteration of Emotion Regulation Precedes the Deficits in Interval Timing in the BACHD Rat Model for Huntington Disease
Source: Front Integr Neurosci. 2018 May 9;12:14. doi: 10.3389/fnint.2018.00014 (PMC5954136; doi:10.3389/fnint.2018.00014)
Supplement: Supplementary file 2 [file Data_Sheet_2.PDF]

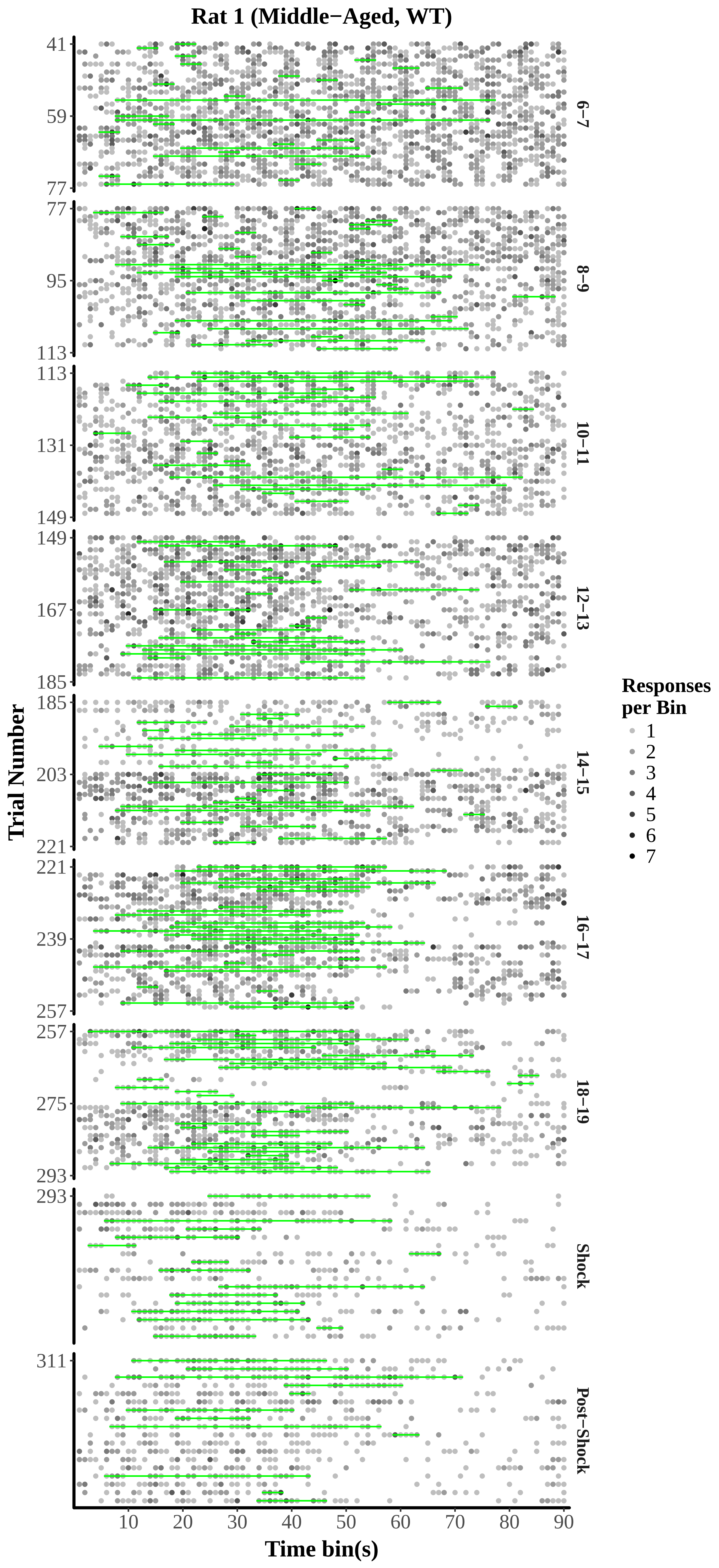

Rat 1 (Middle−Aged, WT)
Trial Number
Time bin(s)
Responses per Bin
1
2
3
4
5
6
7
6−7
8−9
10−11
12−13
14−15
16−17
18−19
Shock
Post−Shock
41
59
77
95
113
131
149
167
185
203
221
239
257
275
293
311
10
20
30
40
50
60
70
80
90

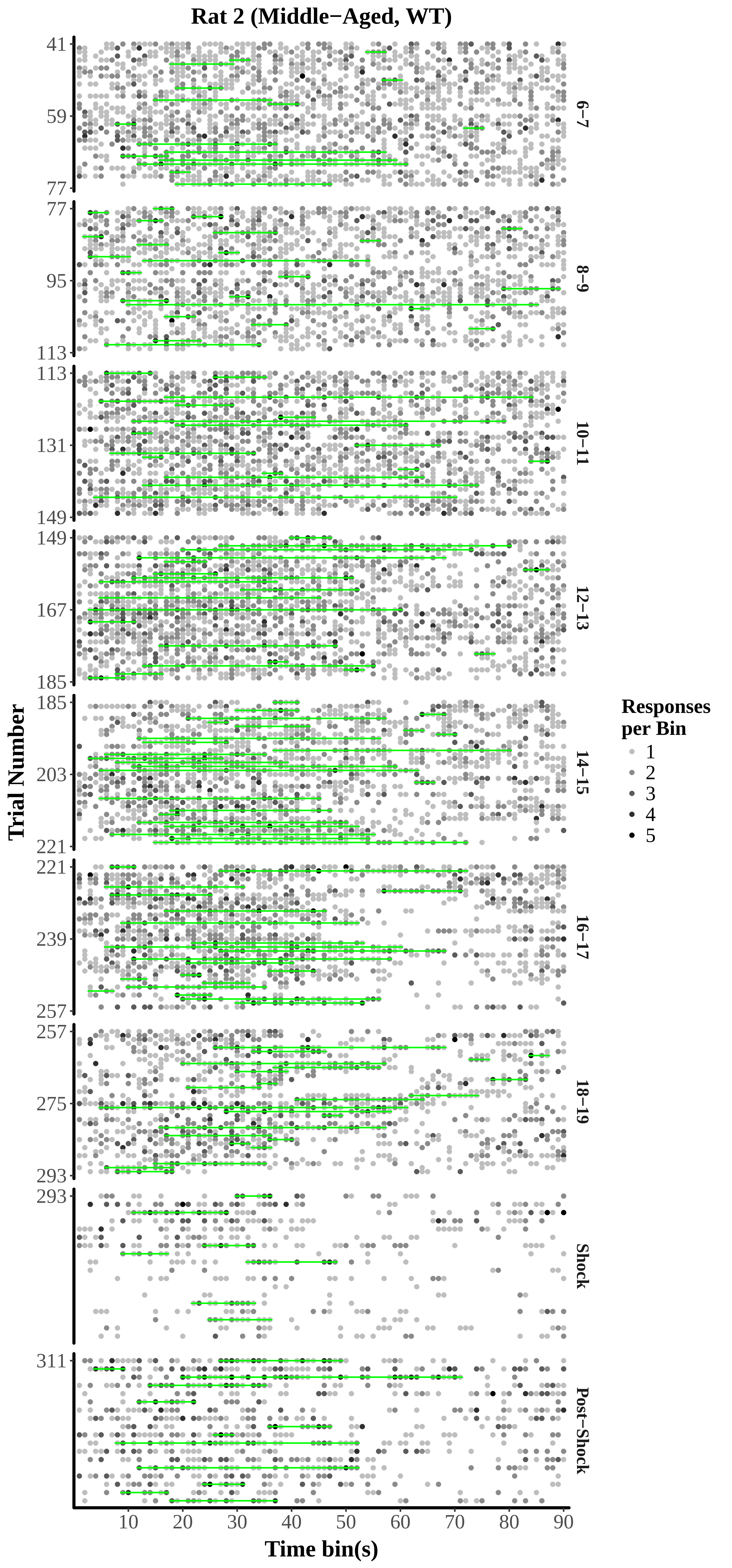

Rat 2 (Middle−Aged, WT)
Trial Number
Time bin(s)
10
20
30
40
50
60
70
80
90
41
59
77
95
113
131
149
167
185
203
221
239
257
275
293
311
6−7
8−9
10−11
12−13
14−15
16−17
18−19
Shock
Post−Shock
Responses per Bin
1
2
3
4
5

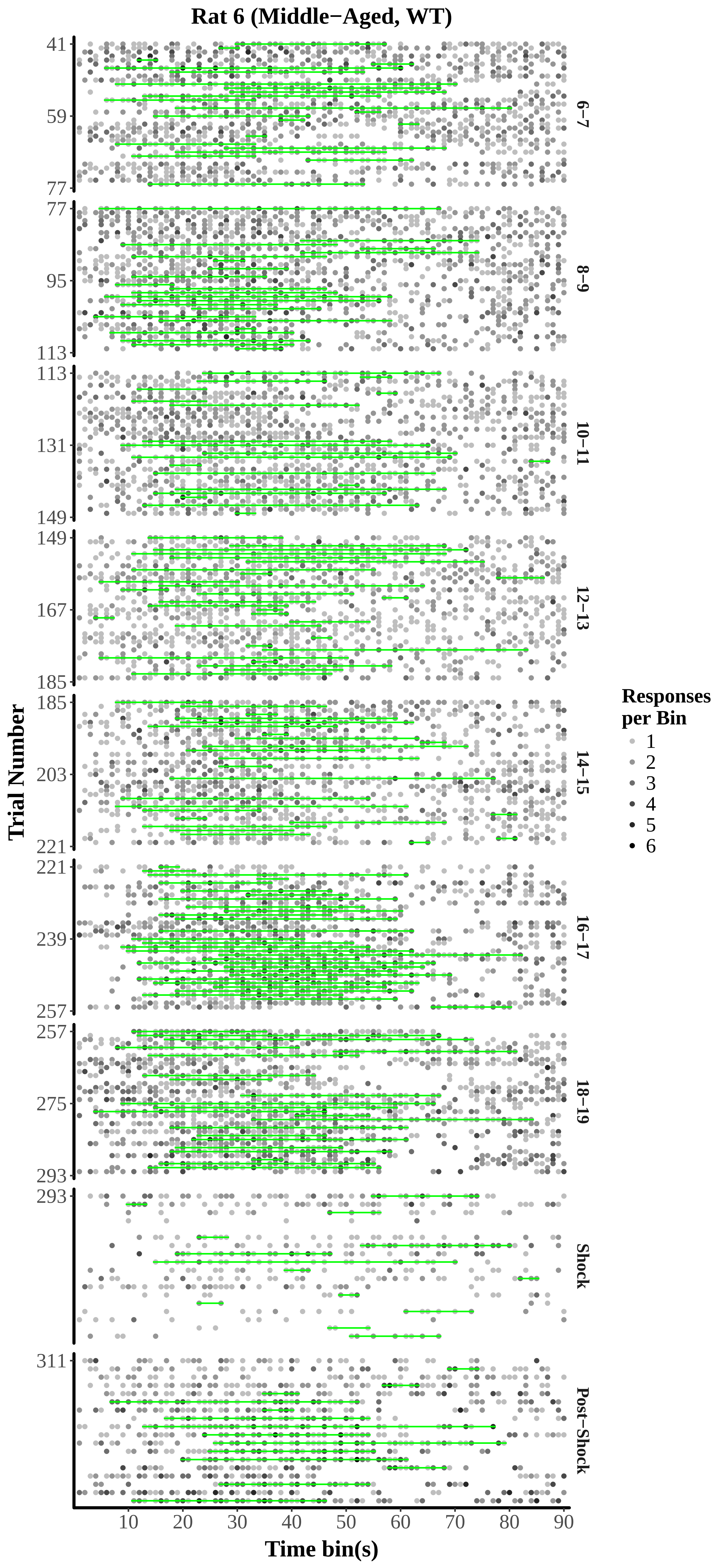
Rat 6 (Middle−Aged, WT)
Trial Number
41
59
77
95
113
131
149
167
185
203
221
239
257
275
293
311
6–7
8–9
10–11
12–13
14–15
16–17
18–19
Shock
Post−Shock
10
20
30
40
50
60
70
80
90
Time bin(s)
Responses per Bin
1
2
3
4
5
6

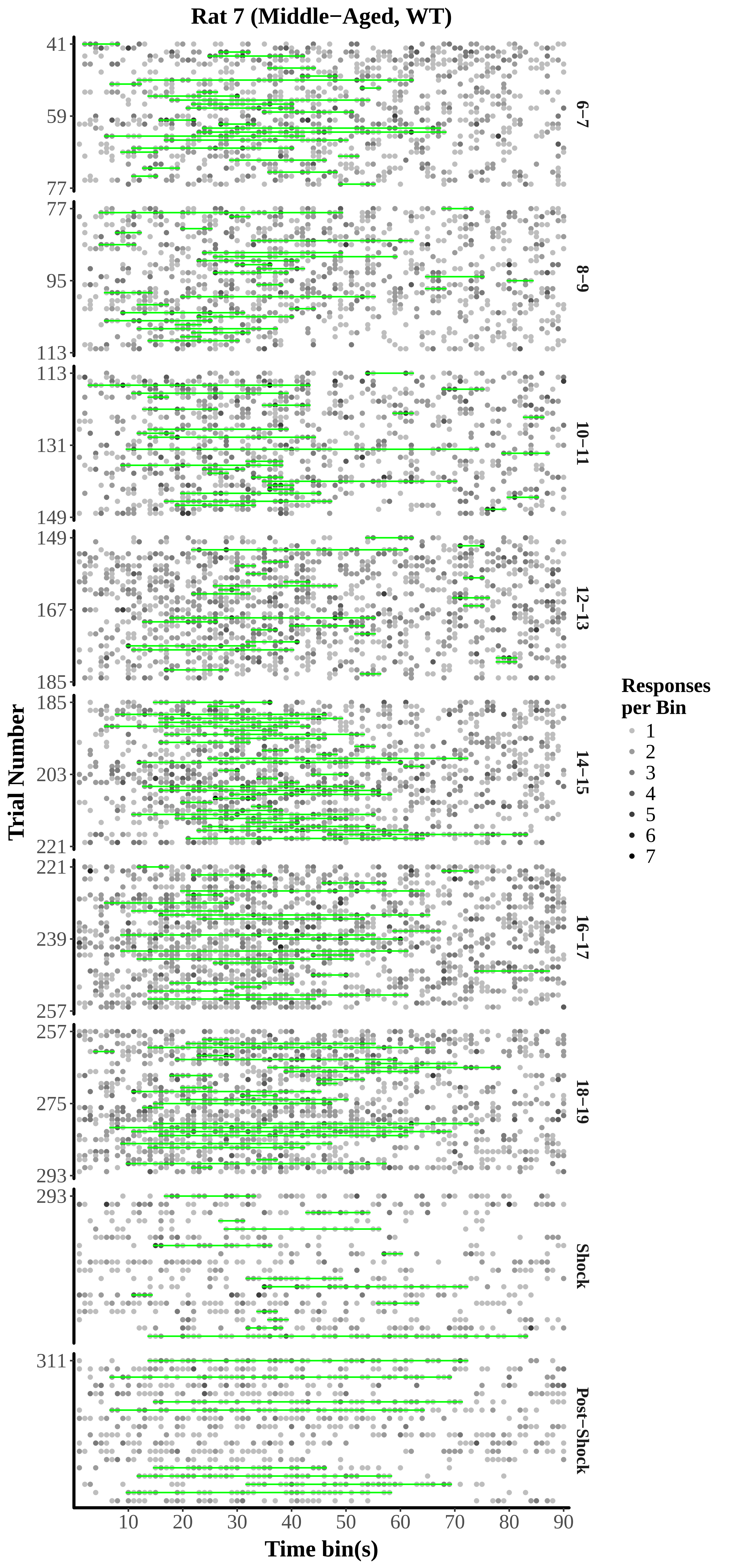
Rat 7 (Middle−Aged, WT)
Trial Number
41
59
77
95
113
131
149
167
185
203
221
239
257
275
293
311
6−7
8−9
10−11
12−13
14−15
16−17
18−19
Shock
Post−Shock
Time bin(s)
10
20
30
40
50
60
70
80
90
Responses per Bin
1
2
3
4
5
6
7

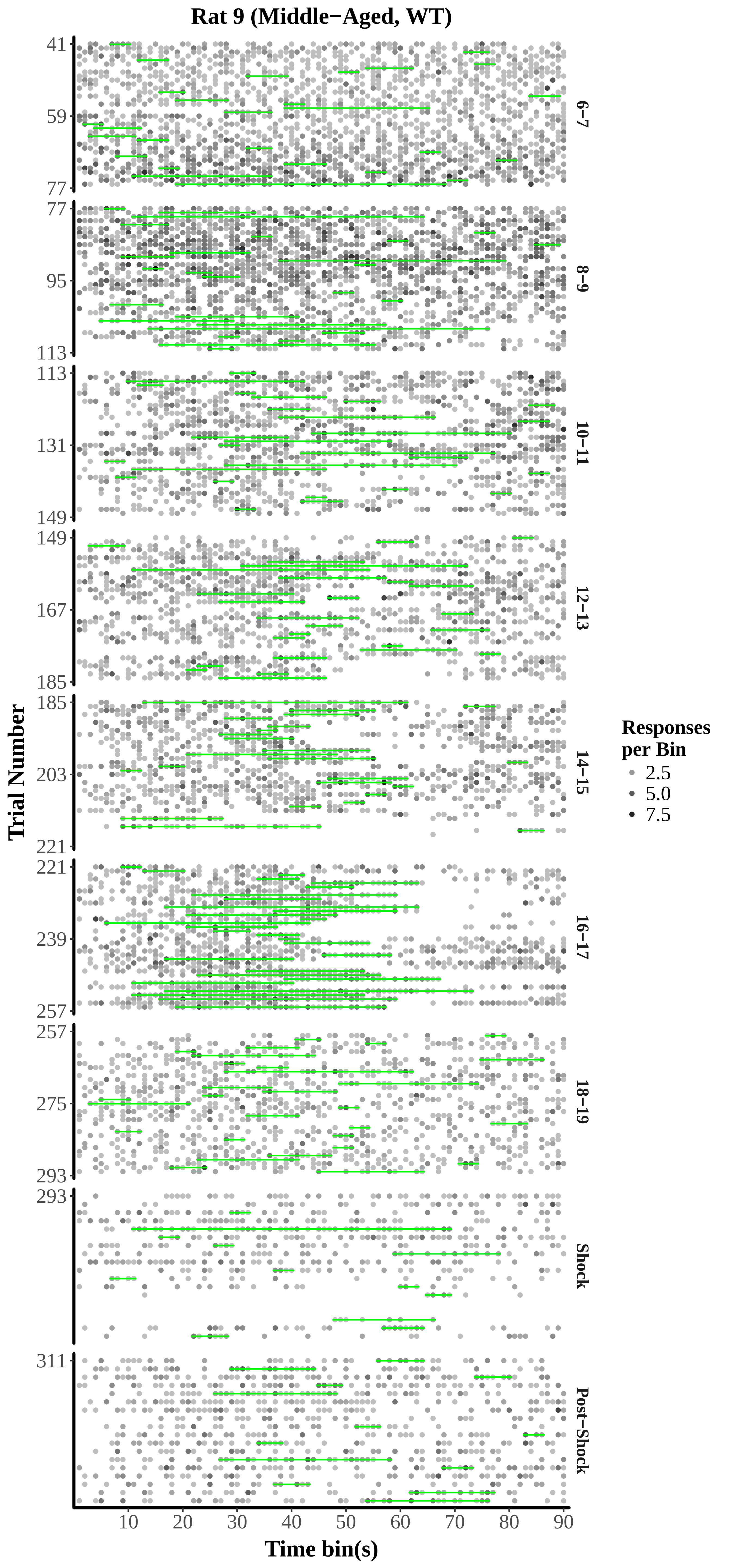

Rat 9 (Middle−Aged, WT)
Trial Number
Time bin(s)
6–7
8–9
10–11
12–13
14–15
16–17
18–19
Shock
Post−Shock
Responses per Bin
2.5
5.0
7.5
41
59
77
95
113
131
149
167
185
203
221
239
257
275
293
311
10
20
30
40
50
60
70
80
90

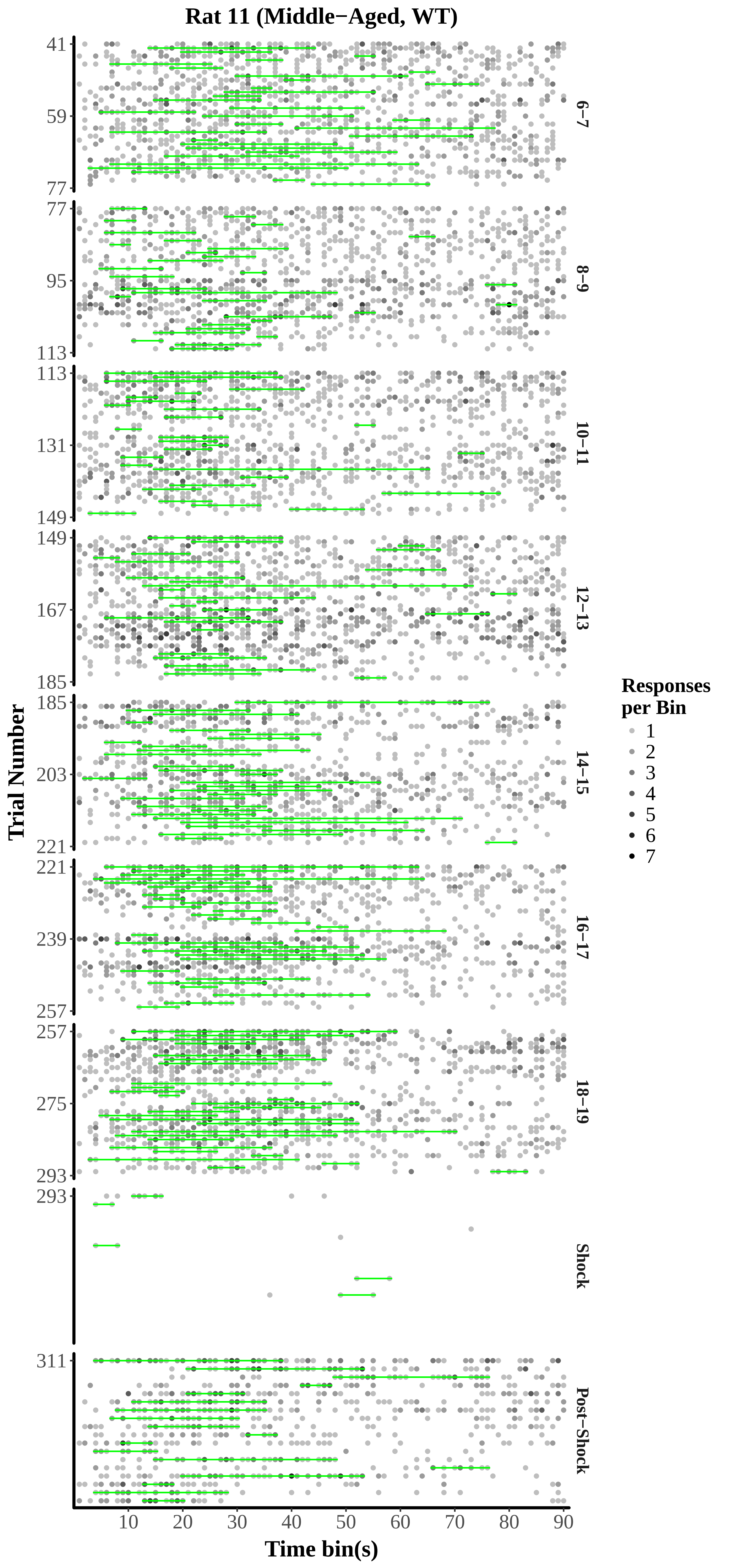
Rat 11 (Middle−Aged, WT)
Trial Number
Time bin(s)
10
20
30
40
50
60
70
80
90
41
59
77
95
113
131
149
167
185
203
221
239
257
275
293
311
6−7
8−9
10−11
12−13
14−15
16−17
18−19
Shock
Post−Shock
Responses per Bin
1
2
3
4
5
6
7

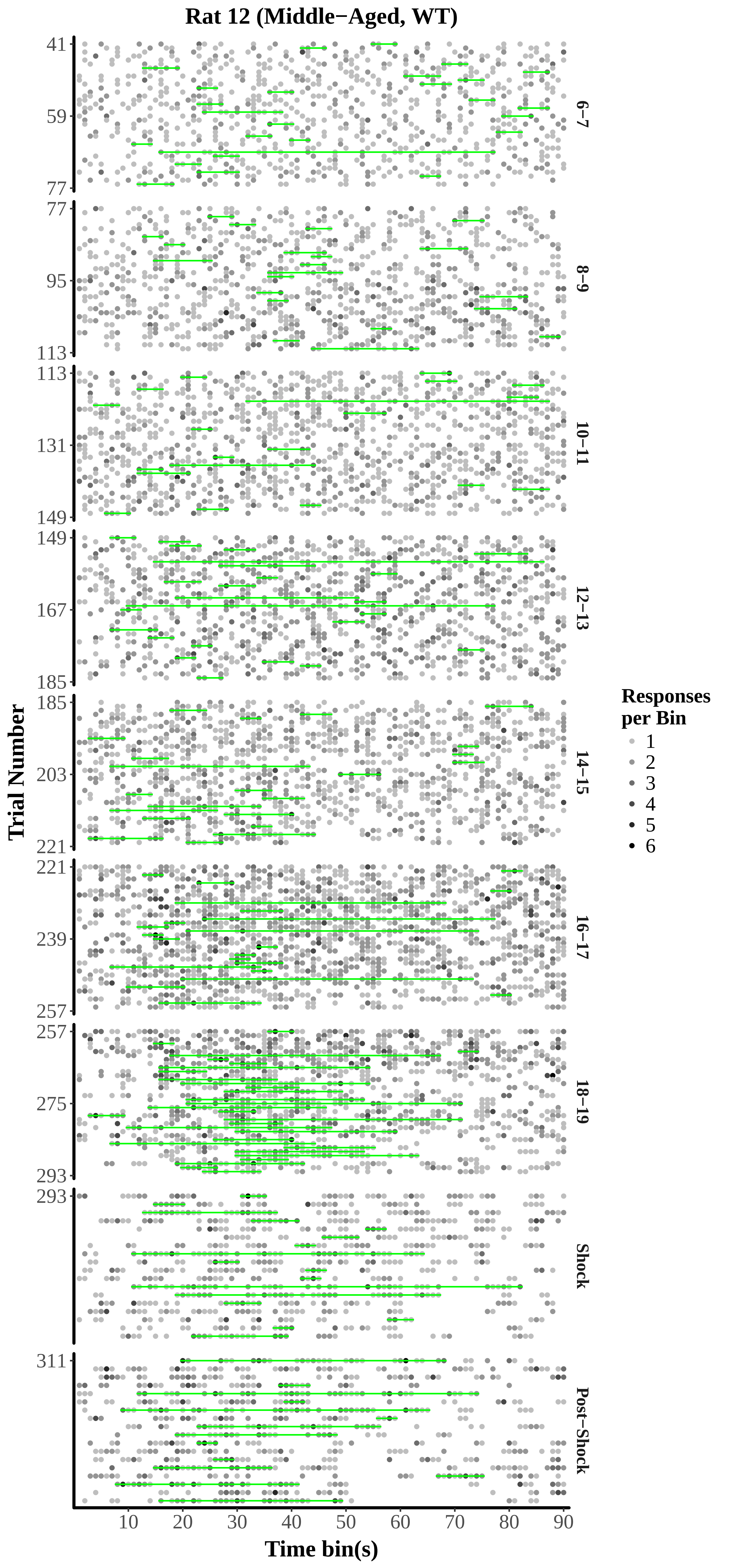

Rat 12 (Middle−Aged, WT)
Trial Number
Time bin(s)
41
59
77
95
113
131
149
167
185
203
221
239
257
275
293
311
10
20
30
40
50
60
70
80
90
6−7
8−9
10−11
12−13
14−15
16−17
18−19
Shock
Post−Shock
Responses per Bin
1
2
3
4
5
6

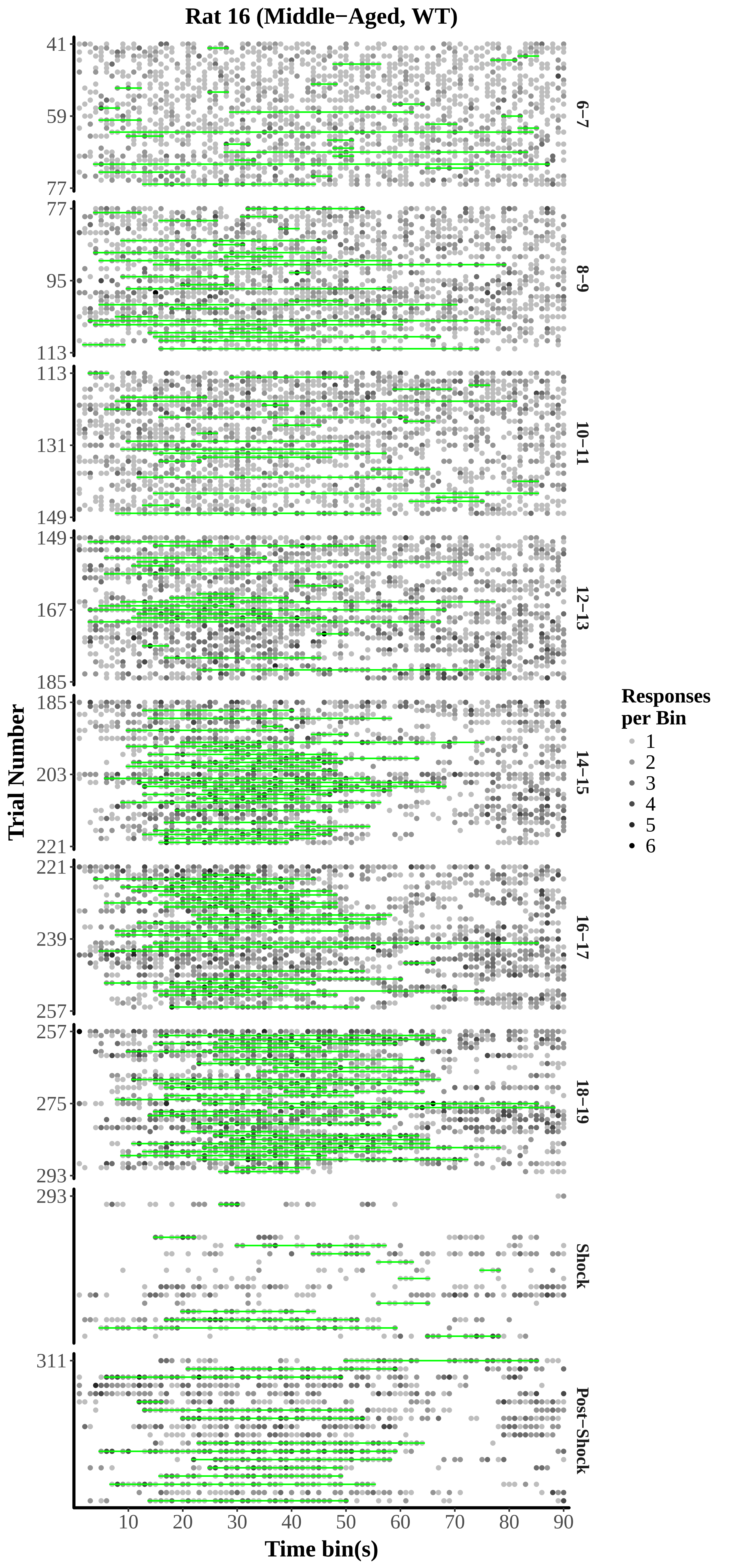

Rat 16 (Middle−Aged, WT)
Trial Number
Time bin(s)
10 20 30 40 50 60 70 80 90
41 59 77
77 95 113
113 131 149
149 167 185
185 203 221
221 239 257
257 275 293
293
311
6−7
8−9
10−11
12−13
14−15
16−17
18−19
Shock
Post−Shock
Responses per Bin
1
2
3
4
5
6

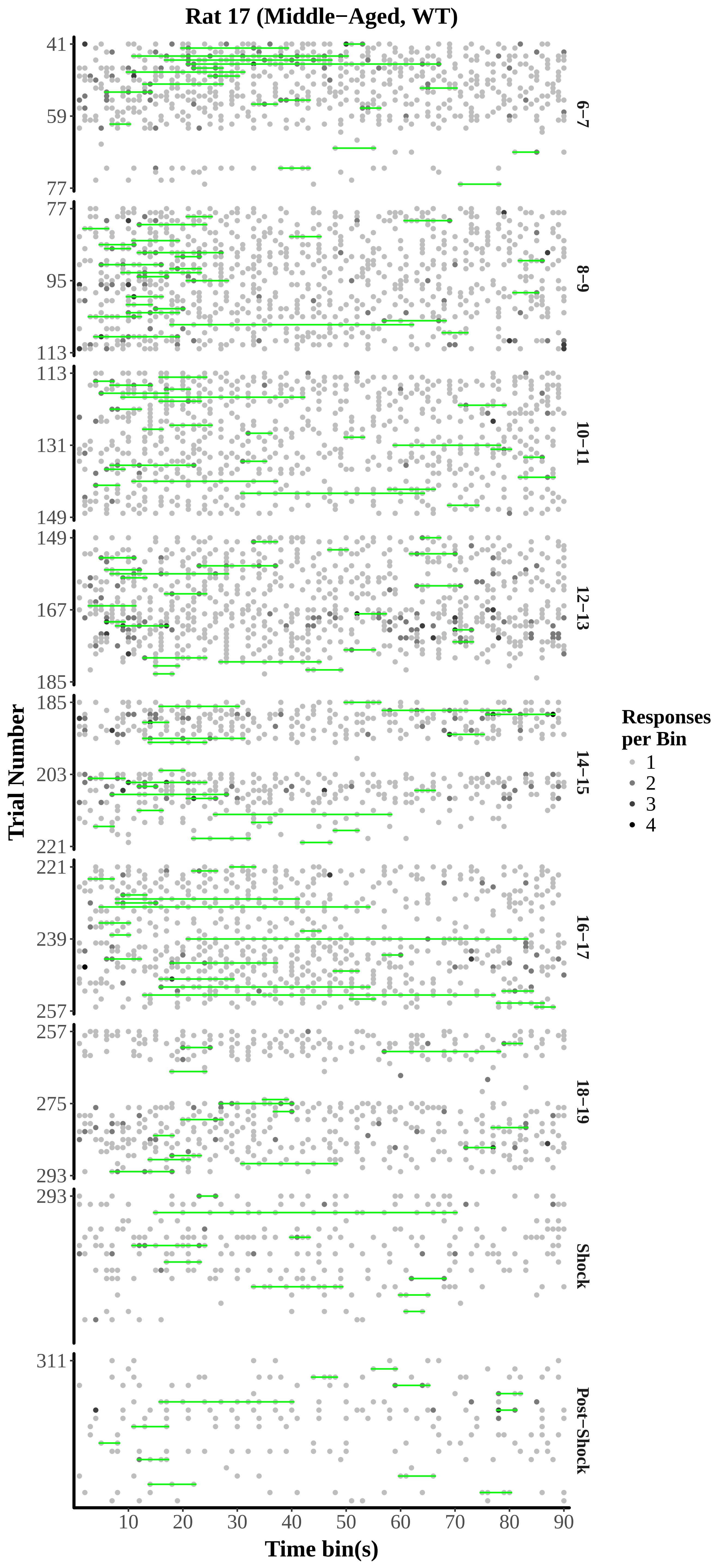

Rat 17 (Middle−Aged, WT)
Trial Number
41
59
77
95
113
131
149
167
185
203
221
239
257
275
293
311
6−7
8−9
10−11
12−13
14−15
16−17
18−19
Shock
Post−Shock
10
20
30
40
50
60
70
80
90
Time bin(s)
Responses per Bin
1
2
3
4

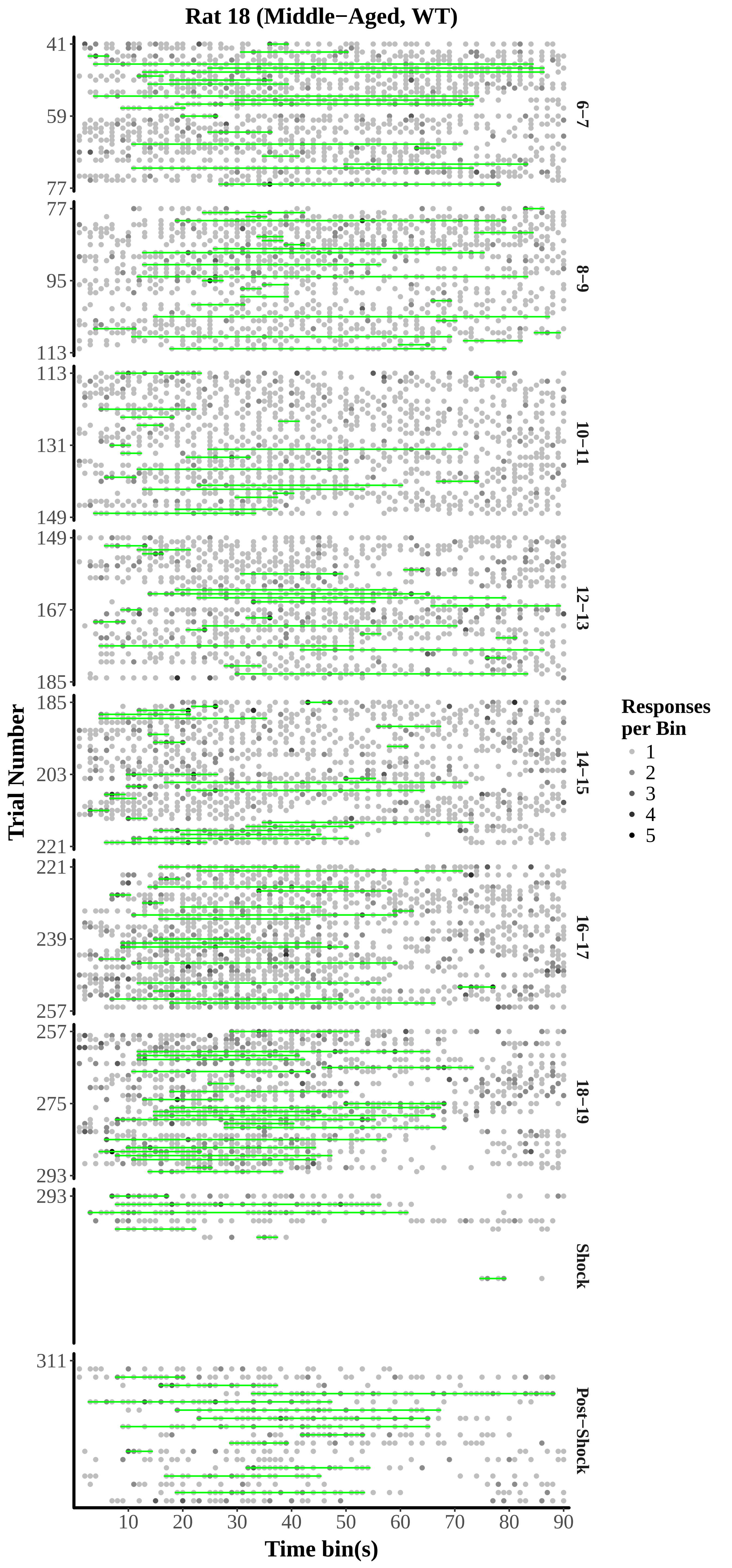

Rat 18 (Middle−Aged, WT)
Trial Number
Time bin(s)
10
20
30
40
50
60
70
80
90
41
59
77
95
113
131
149
167
185
203
221
239
257
275
293
311
6–7
8–9
10–11
12–13
14–15
16–17
18–19
Shock
Post−Shock
Responses per Bin
1
2
3
4
5

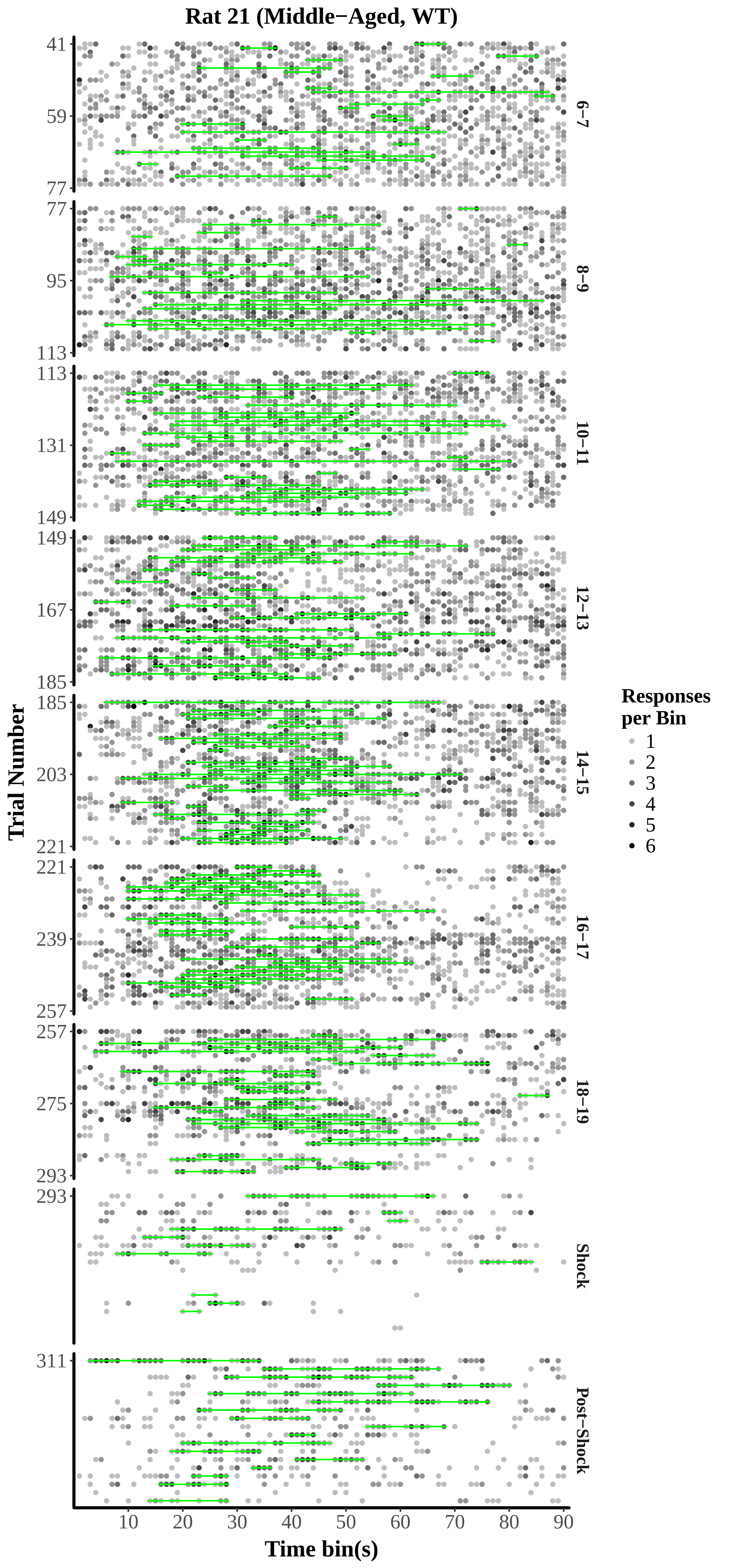

Rat 21 (Middle−Aged, WT)
Trial Number
Time bin(s)
10
20
30
40
50
60
70
80
90
41
59
77
95
113
131
149
167
185
203
221
239
257
275
293
311
6−7
8−9
10−11
12−13
14−15
16−17
18−19
Shock
Post−Shock
Responses per Bin
1
2
3
4
5
6

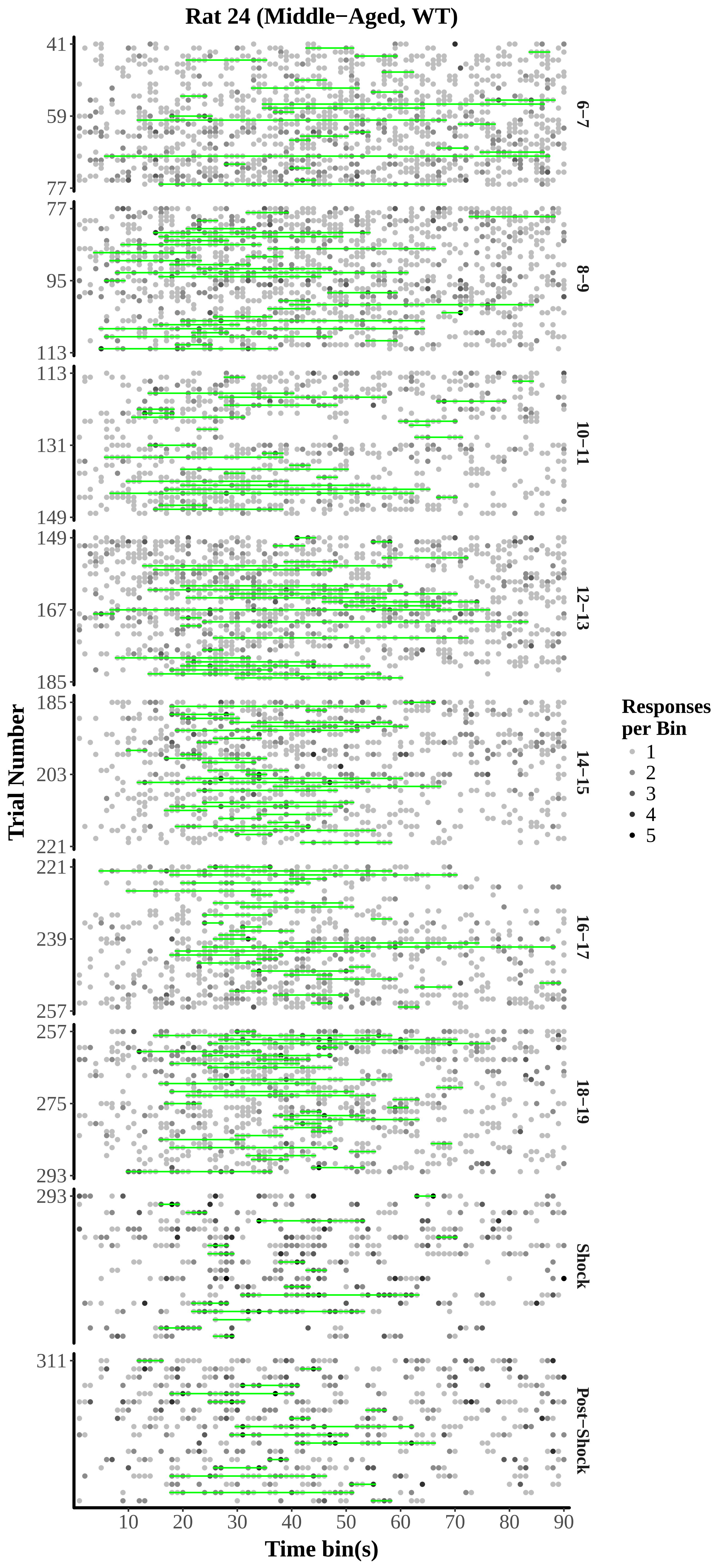
Rat 24 (Middle−Aged, WT)
Trial Number
Time bin(s)
6−7
8−9
10−11
12−13
14−15
16−17
18−19
Shock
Post−Shock
41
59
77
95
113
131
149
167
185
203
221
239
257
275
293
311
10
20
30
40
50
60
70
80
90
Responses per Bin
1
2
3
4
5
