# Supplementary material for: The Alteration of Emotion Regulation Precedes the Deficits in Interval Timing in the BACHD Rat Model for Huntington Disease
Source: Front Integr Neurosci. 2018 May 9;12:14. doi: 10.3389/fnint.2018.00014 (PMC5954136; doi:10.3389/fnint.2018.00014)
Supplement: Supplementary file 3 [file Data_Sheet_3.PDF]

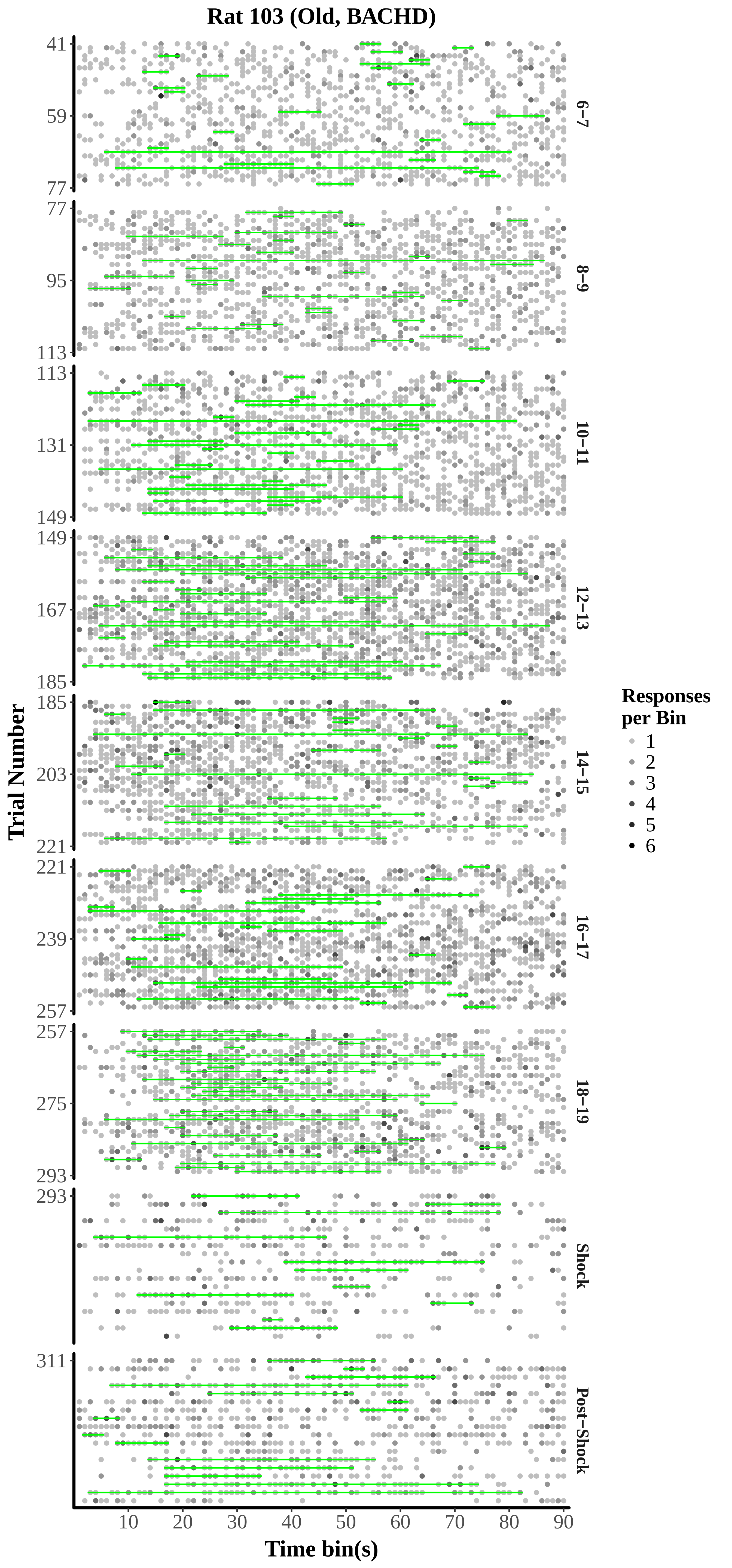

Rat 103 (Old, BACHD)
Trial Number
Time bin(s)
10
20
30
40
50
60
70
80
90
41
59
77
95
113
131
149
167
185
203
221
239
257
275
293
311
6–7
8–9
10–11
12–13
14–15
16–17
18–19
Shock
Post–Shock
Responses per Bin
1
2
3
4
5
6

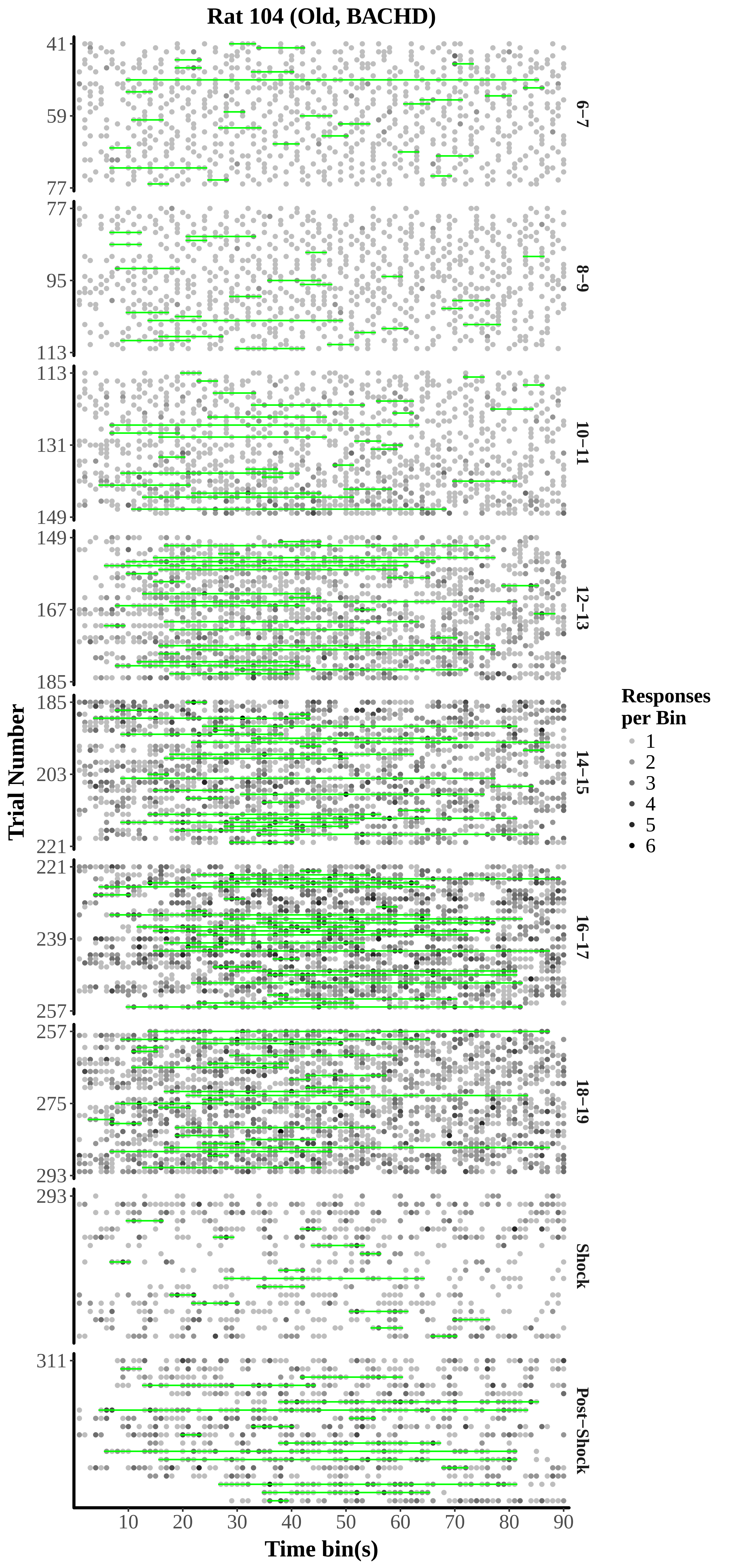

Rat 104 (Old, BACHD)
Trial Number
41
59
77
95
113
131
149
167
185
203
221
239
257
275
293
311
6–7
8–9
10–11
12–13
14–15
16–17
18–19
Shock
Post–Shock
10
20
30
40
50
60
70
80
90
Time bin(s)
Responses per Bin
1
2
3
4
5
6

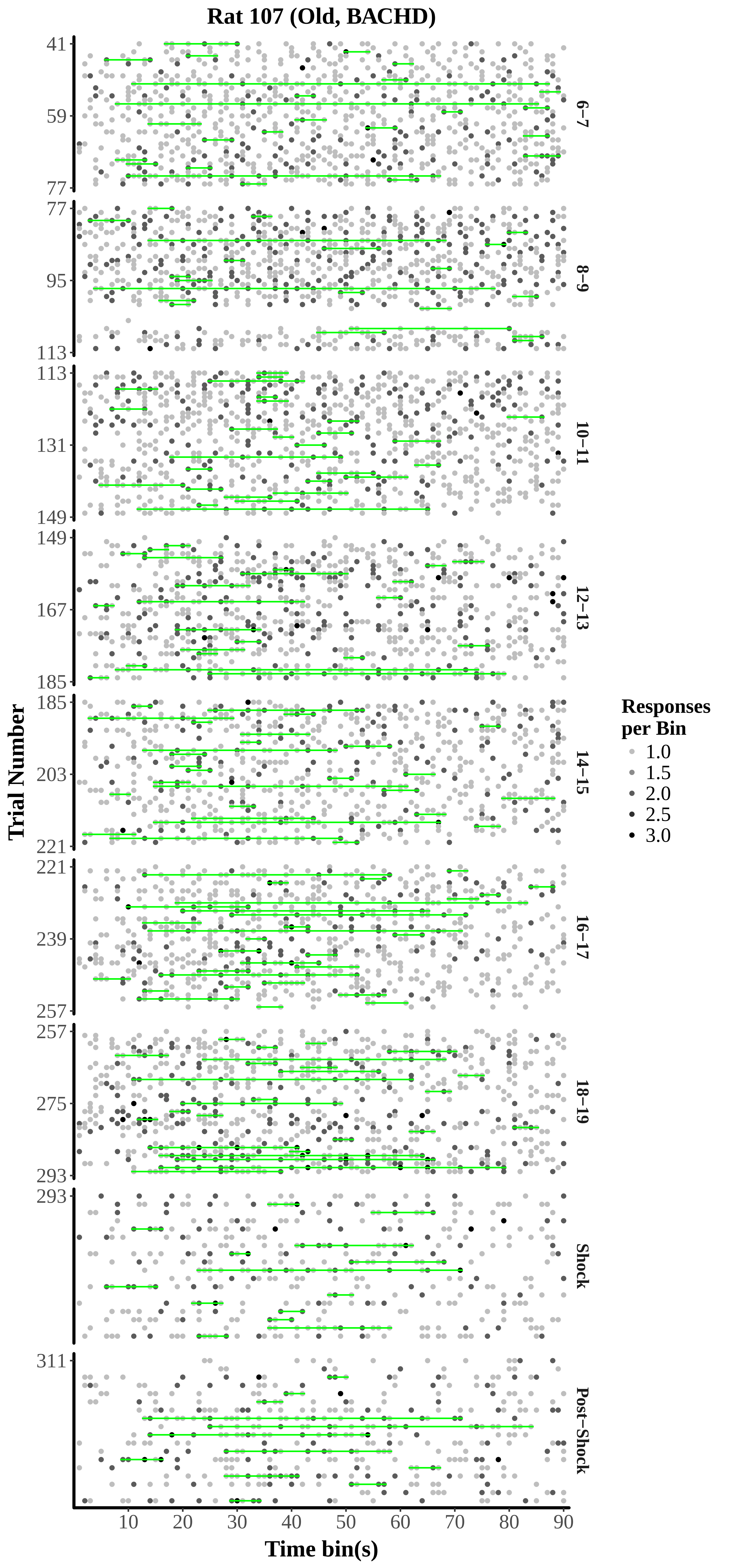

Rat 107 (Old, BACHD)
Trial Number
Time bin(s)
6–7
8–9
10–11
12–13
14–15
16–17
18–19
Shock
Post–Shock
Responses per Bin
1.0
1.5
2.0
2.5
3.0
41
59
77
95
113
131
149
167
185
203
221
239
257
275
293
311
10
20
30
40
50
60
70
80
90

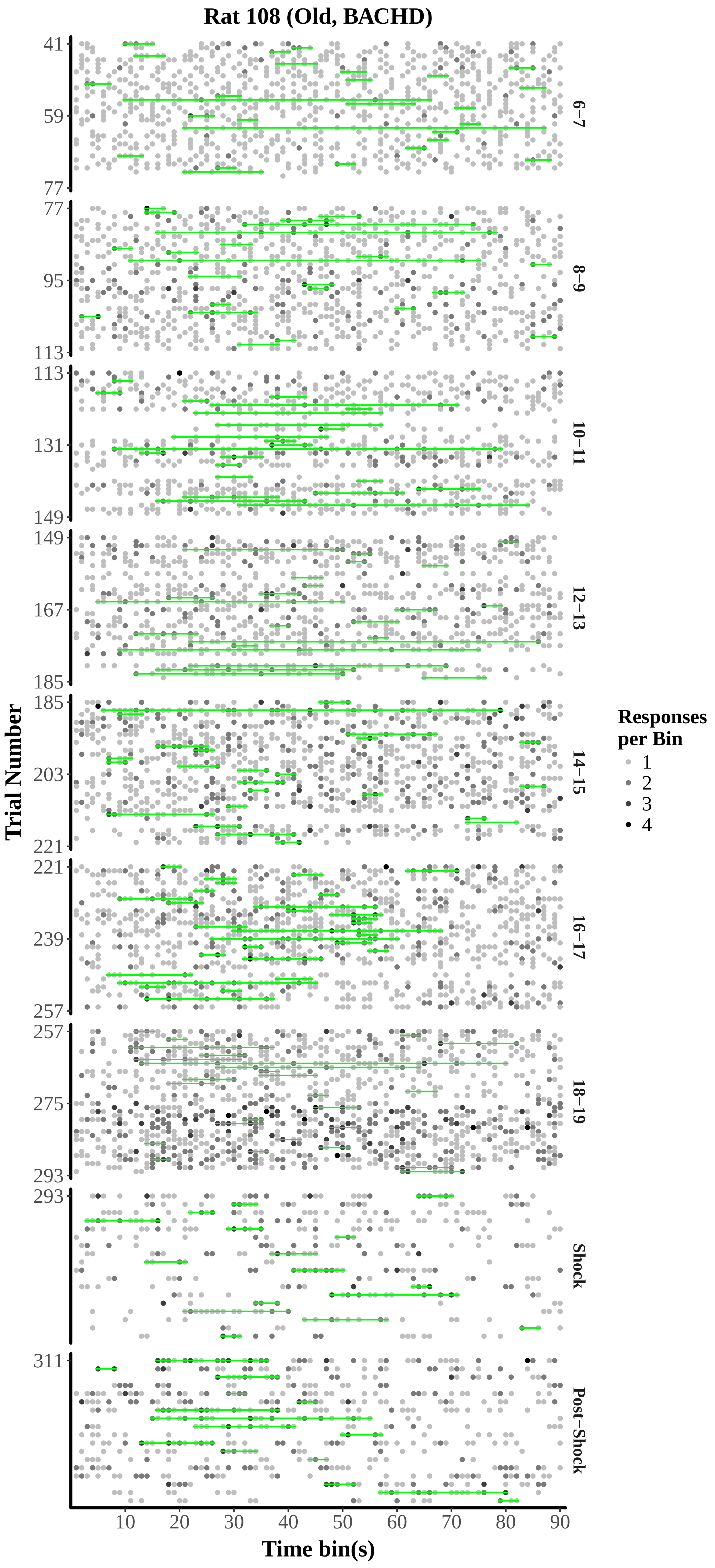
Rat 108 (Old, BACHD)
Trial Number
Time bin(s)
10
20
30
40
50
60
70
80
90
41
59
77
95
113
131
149
167
185
203
221
239
257
275
293
311
6–7
8–9
10–11
12–13
14–15
16–17
18–19
Shock
Post–Shock
Responses per Bin
1
2
3
4

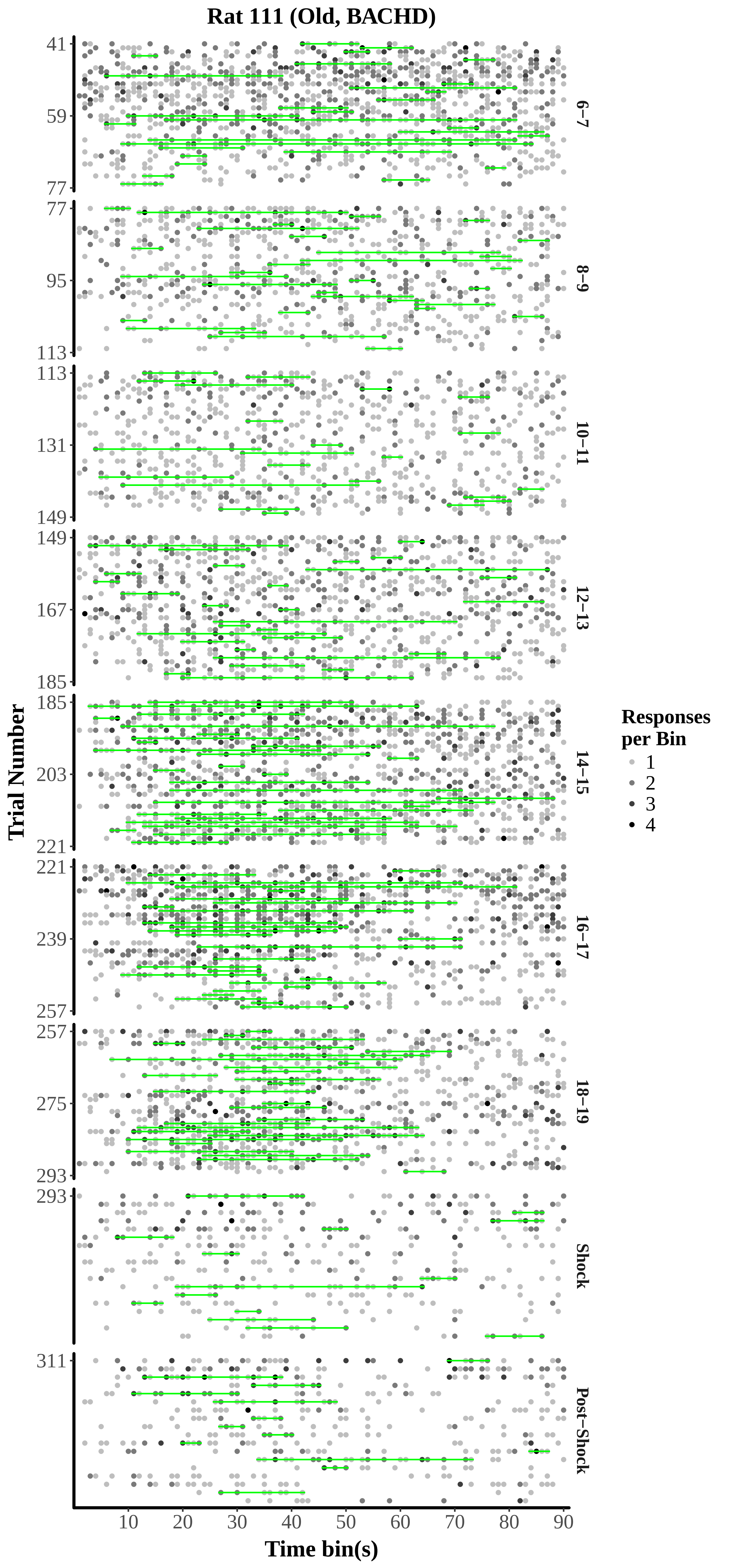

Rat 111 (Old, BACHD)
Trial Number
Time bin(s)
10
20
30
40
50
60
70
80
90
41
59
77
95
113
131
149
167
185
203
221
239
257
275
293
311
6–7
8–9
10–11
12–13
14–15
16–17
18–19
Shock
Post–Shock
Responses per Bin
1
2
3
4

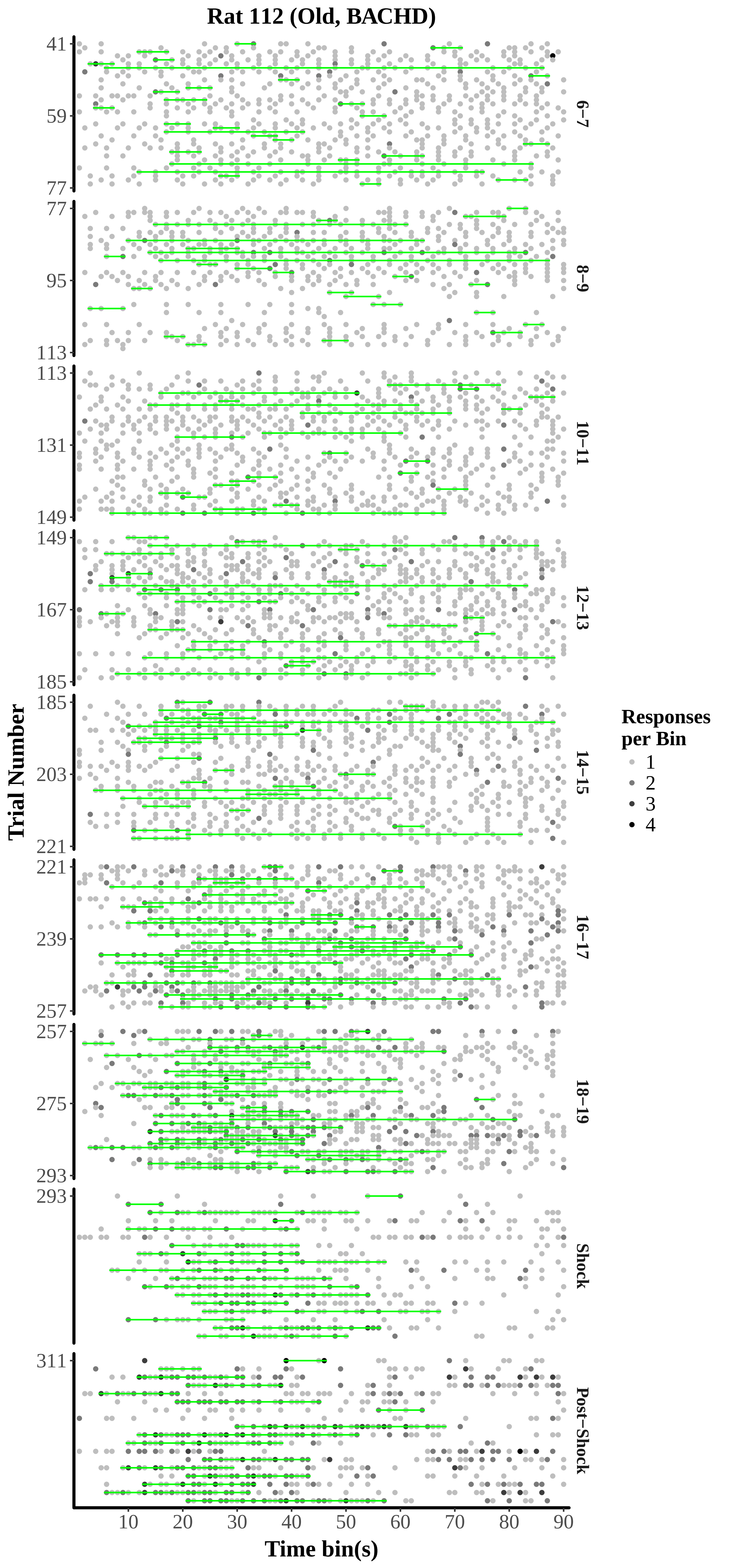

Rat 112 (Old, BACHD)
Trial Number
Time bin(s)
6–7
8–9
10–11
12–13
14–15
16–17
18–19
Shock
Post–Shock
Responses per Bin
1
2
3
4
41
59
77
95
113
131
149
167
185
203
221
239
257
275
293
311
10
20
30
40
50
60
70
80
90

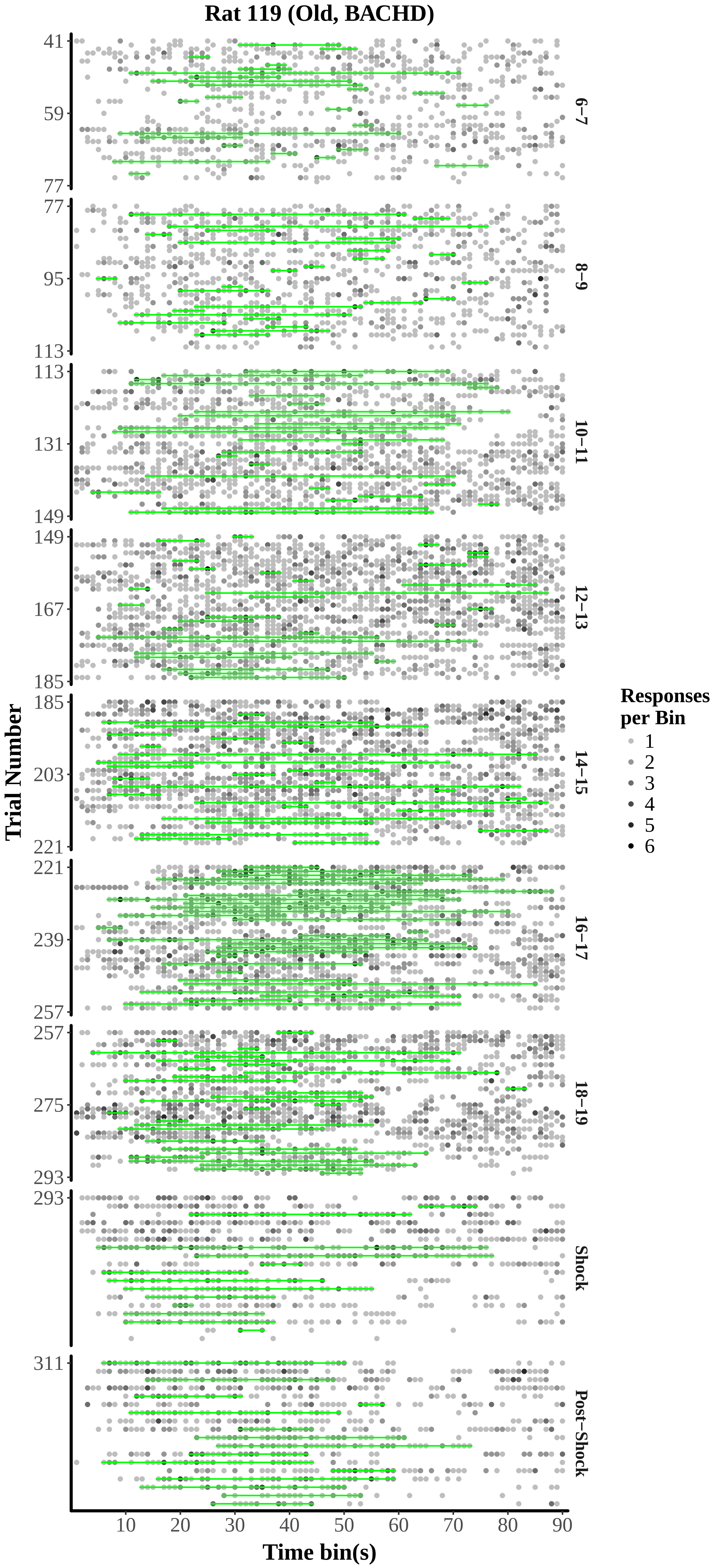
Rat 119 (Old, BACHD)
Trial Number
Time bin(s)
41
59
77
95
113
131
149
167
185
203
221
239
257
275
293
311
10
20
30
40
50
60
70
80
90
6–7
8–9
10–11
12–13
14–15
16–17
18–19
Shock
Post–Shock
Responses per Bin
1
2
3
4
5
6

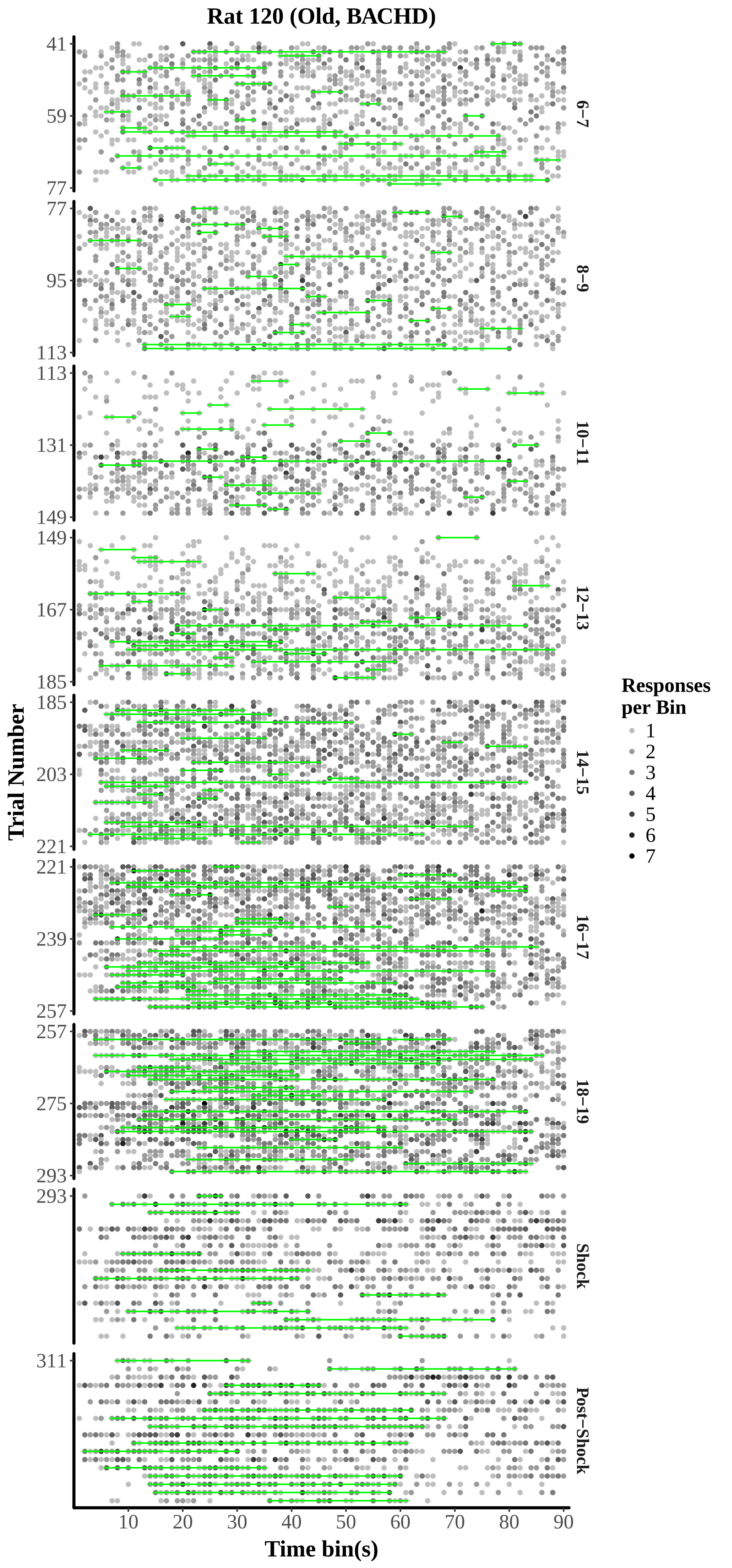
Rat 120 (Old, BACHD)
Trial Number
41
59
77
77
95
113
113
131
149
149
167
185
185
203
221
221
239
257
257
275
293
293
311
6–7
8–9
10–11
12–13
14–15
16–17
18–19
Shock
Post–Shock
10
20
30
40
50
60
70
80
90
Time bin(s)
Responses per Bin
1
2
3
4
5
6
7

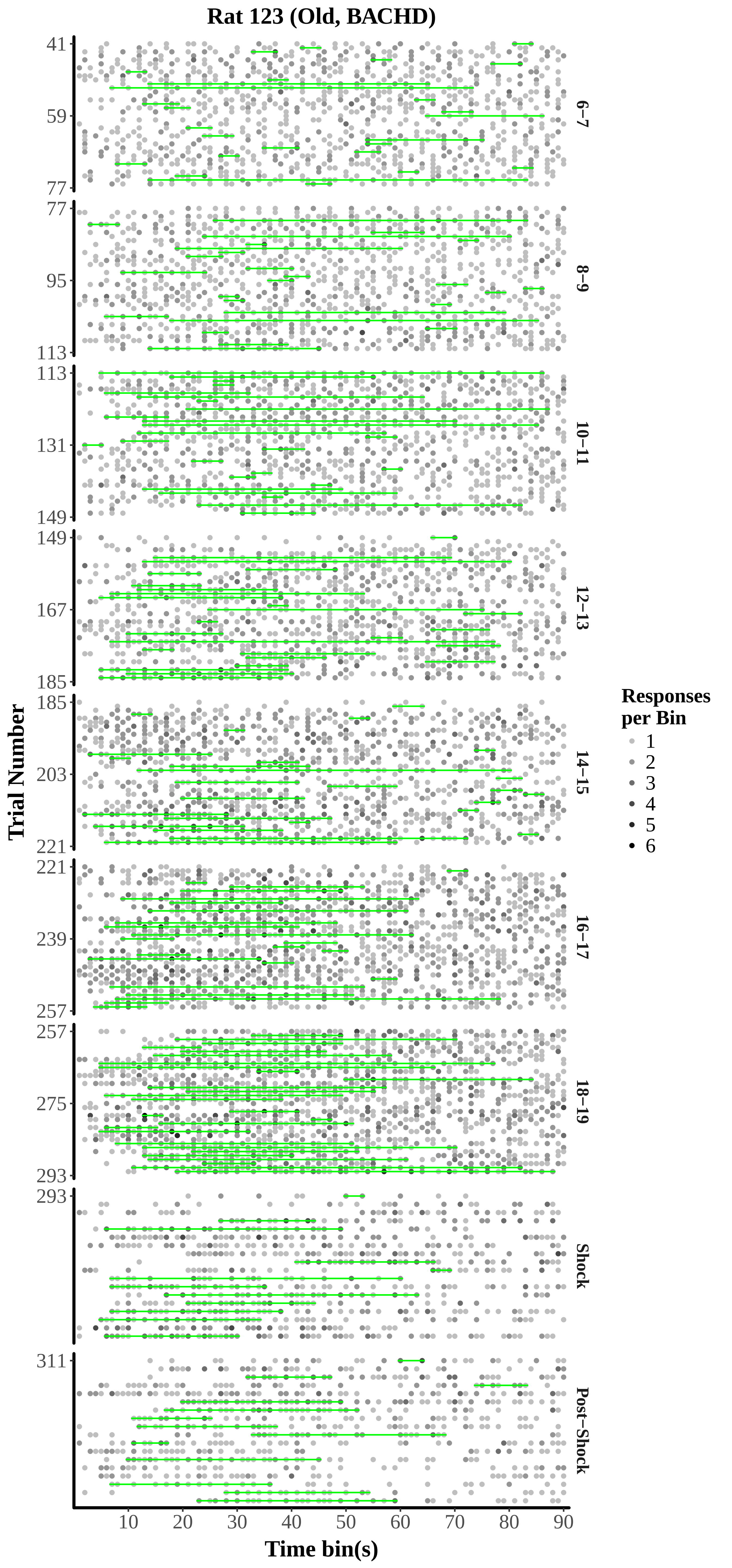

Rat 123 (Old, BACHD)
Trial Number
41
59
77
77
95
113
113
131
149
149
167
185
185
203
221
221
239
257
257
275
293
293
311
6–7
8–9
10–11
12–13
14–15
16–17
18–19
Shock
Post–Shock
Time bin(s)
10
20
30
40
50
60
70
80
90
Responses per Bin
1
2
3
4
5
6

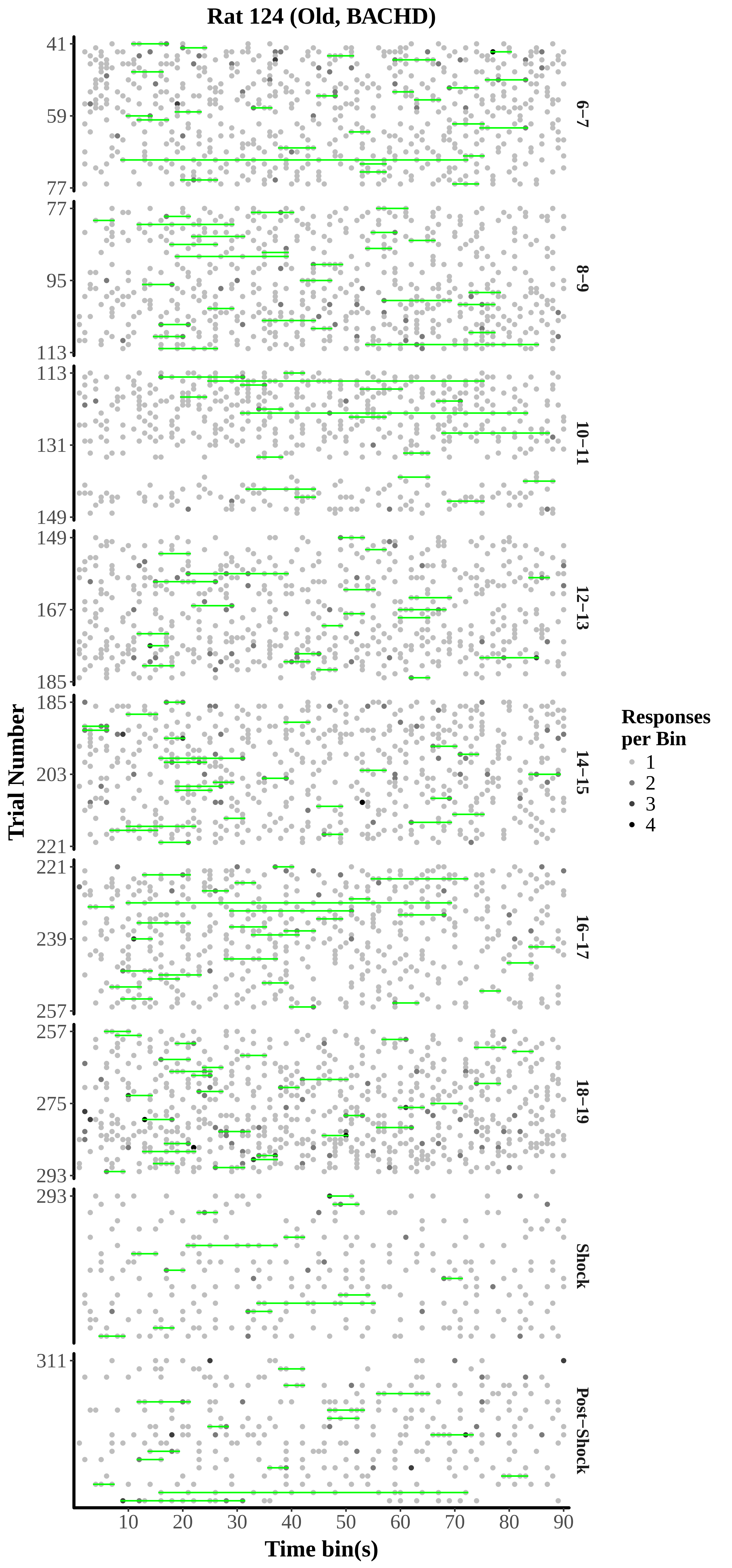

Rat 124 (Old, BACHD)
Trial Number
Time bin(s)
10 20 30 40 50 60 70 80 90
41 59 77
77 95 113
113 131 149
149 167 185
185 203 221
221 239 257
257 275 293
293
311
6–7
8–9
10–11
12–13
14–15
16–17
18–19
Shock
Post–Shock
Responses per Bin
1
2
3
4
