# Supplementary material for: The Alteration of Emotion Regulation Precedes the Deficits in Interval Timing in the BACHD Rat Model for Huntington Disease
Source: Front Integr Neurosci. 2018 May 9;12:14. doi: 10.3389/fnint.2018.00014 (PMC5954136; doi:10.3389/fnint.2018.00014)
Supplement: Supplementary file 4 [file Data_Sheet_4.PDF]

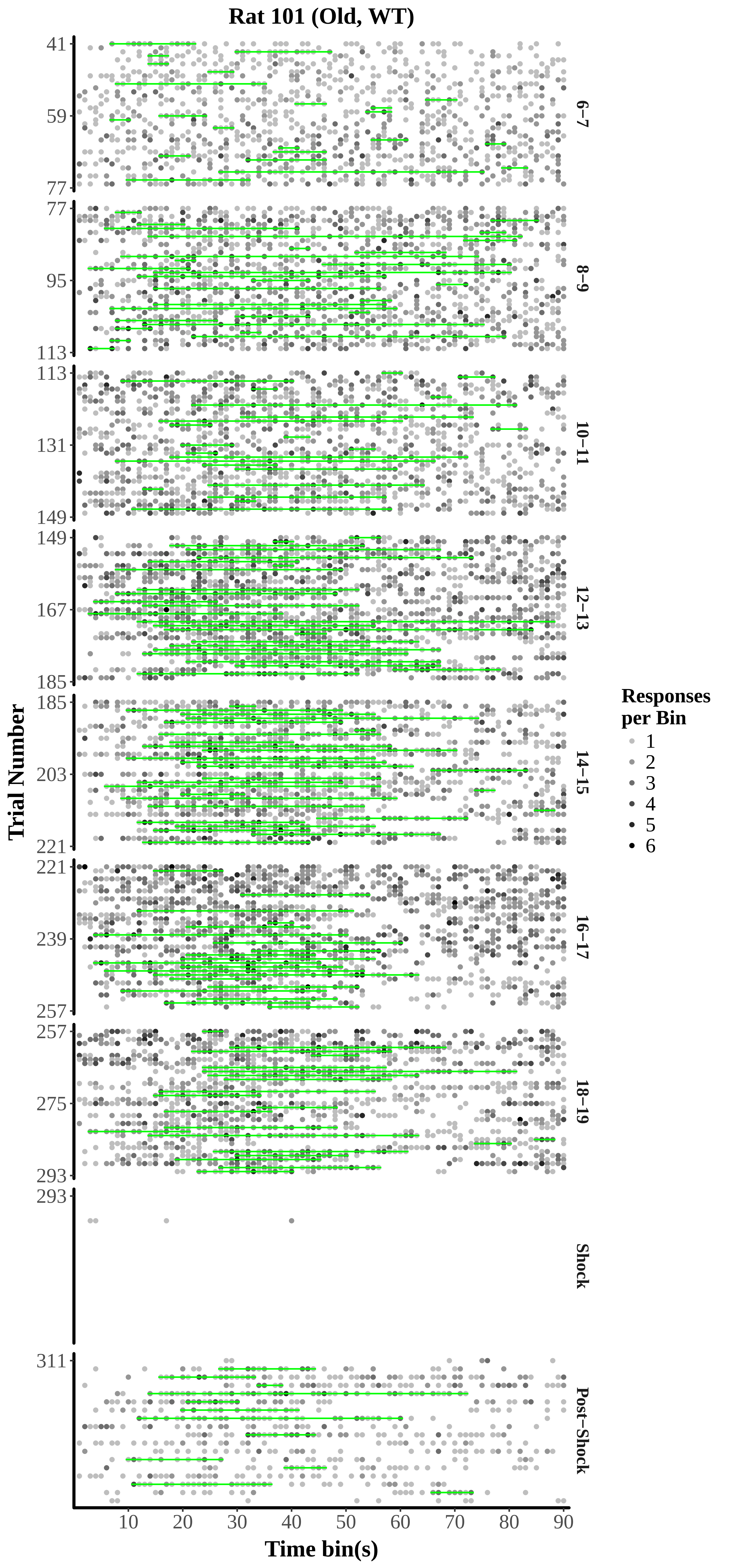

Rat 101 (Old, WT)
Trial Number
Time bin(s)
10 20 30 40 50 60 70 80 90
41 59 77
77 95 113
113 131 149
149 167 185
185 203 221
221 239 257
257 275 293
293
311
6–7
8–9
10–11
12–13
14–15
16–17
18–19
Shock
Post–Shock
Responses per Bin
1
2
3
4
5
6

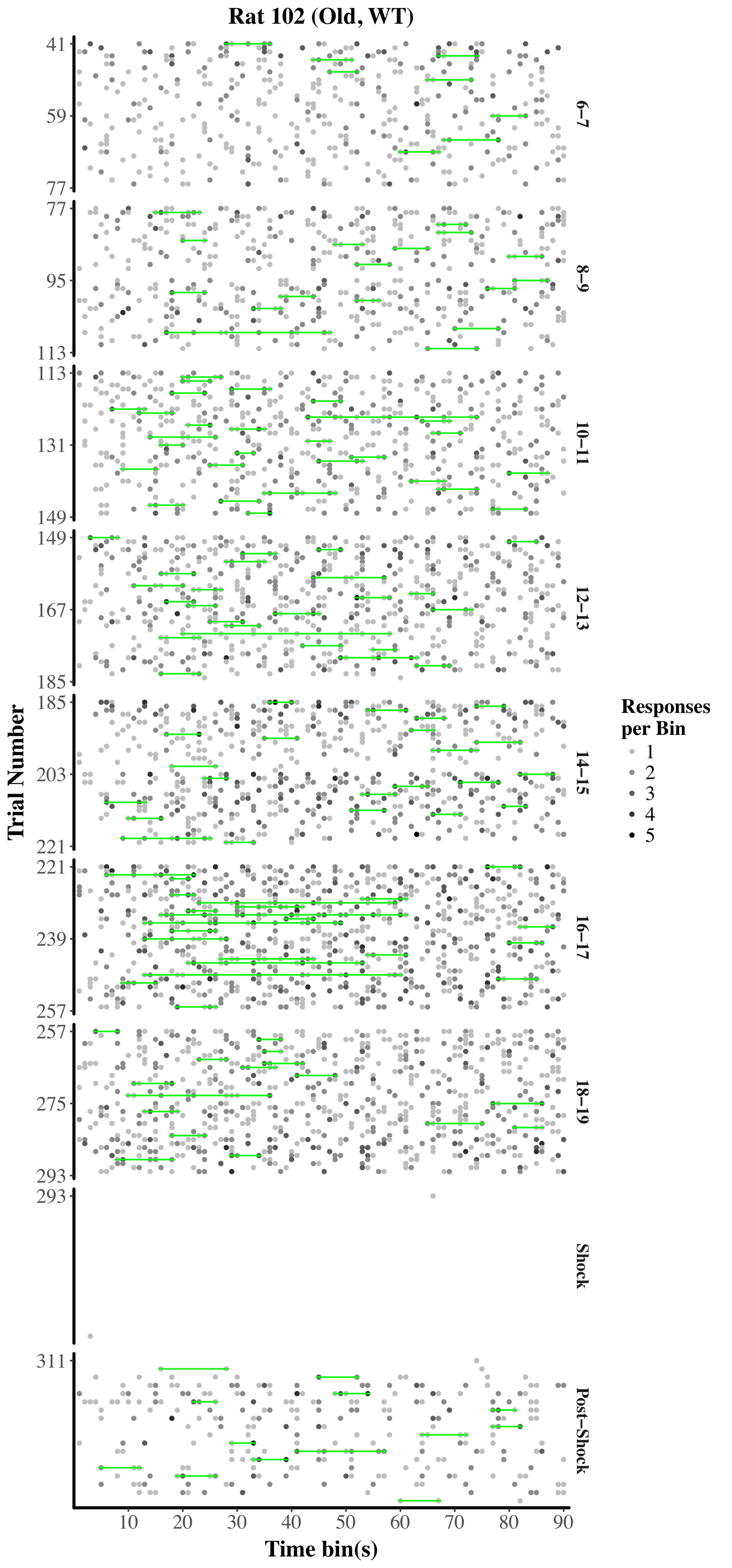
Rat 102 (Old, WT)
Trial Number
Time bin(s)
6–7
8–9
10–11
12–13
14–15
16–17
18–19
Shock
Post–Shock
Responses per Bin
1
2
3
4
5
41
59
77
95
113
131
149
167
185
203
221
239
257
275
293
311
10
20
30
40
50
60
70
80
90

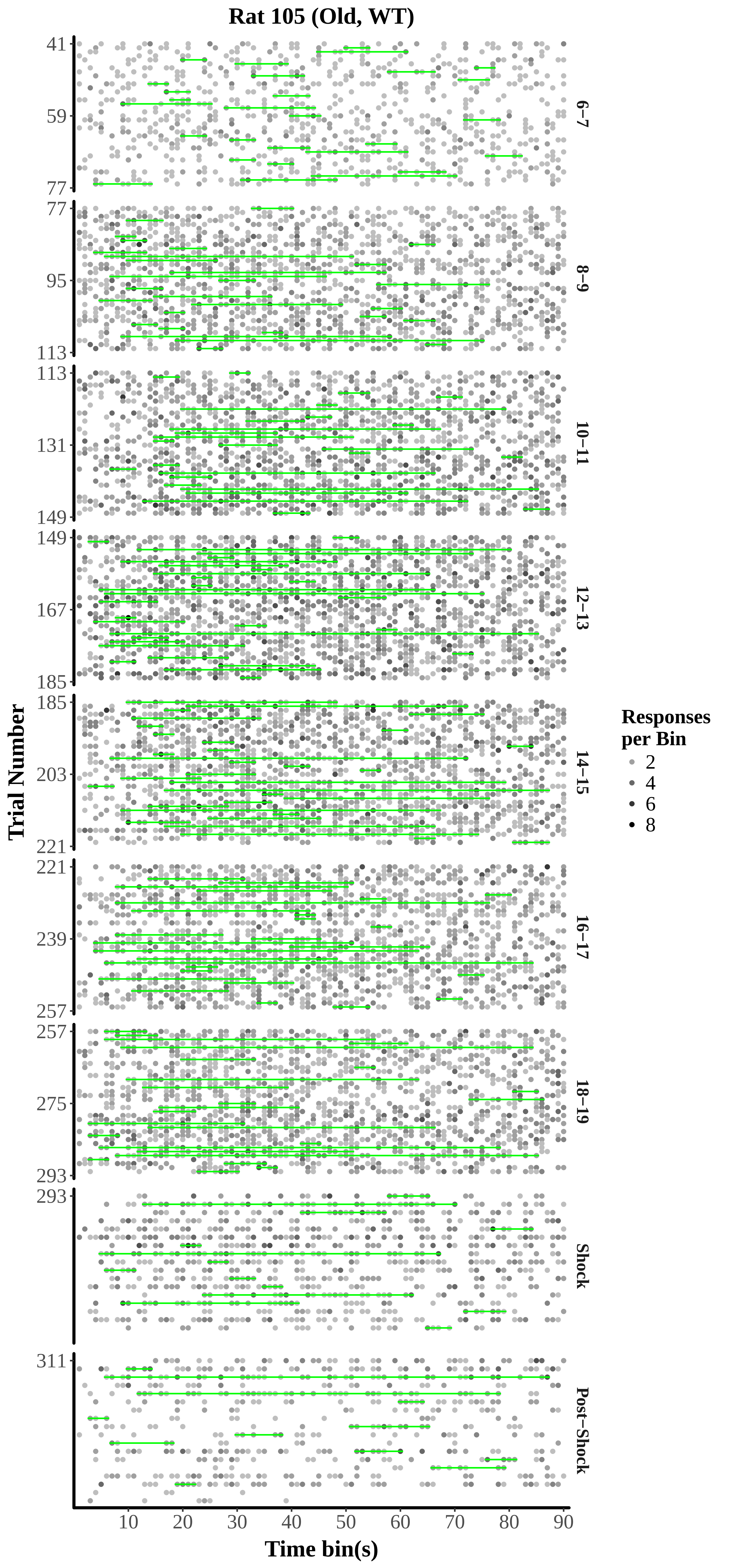

Rat 105 (Old, WT)
Trial Number
Time bin(s)
10 20 30 40 50 60 70 80 90
41 59 77
77 95 113
113 131 149
149 167 185
185 203 221
221 239 257
257 275 293
293
311
6–7
8–9
10–11
12–13
14–15
16–17
18–19
Shock
Post–Shock
Responses per Bin
2
4
6
8

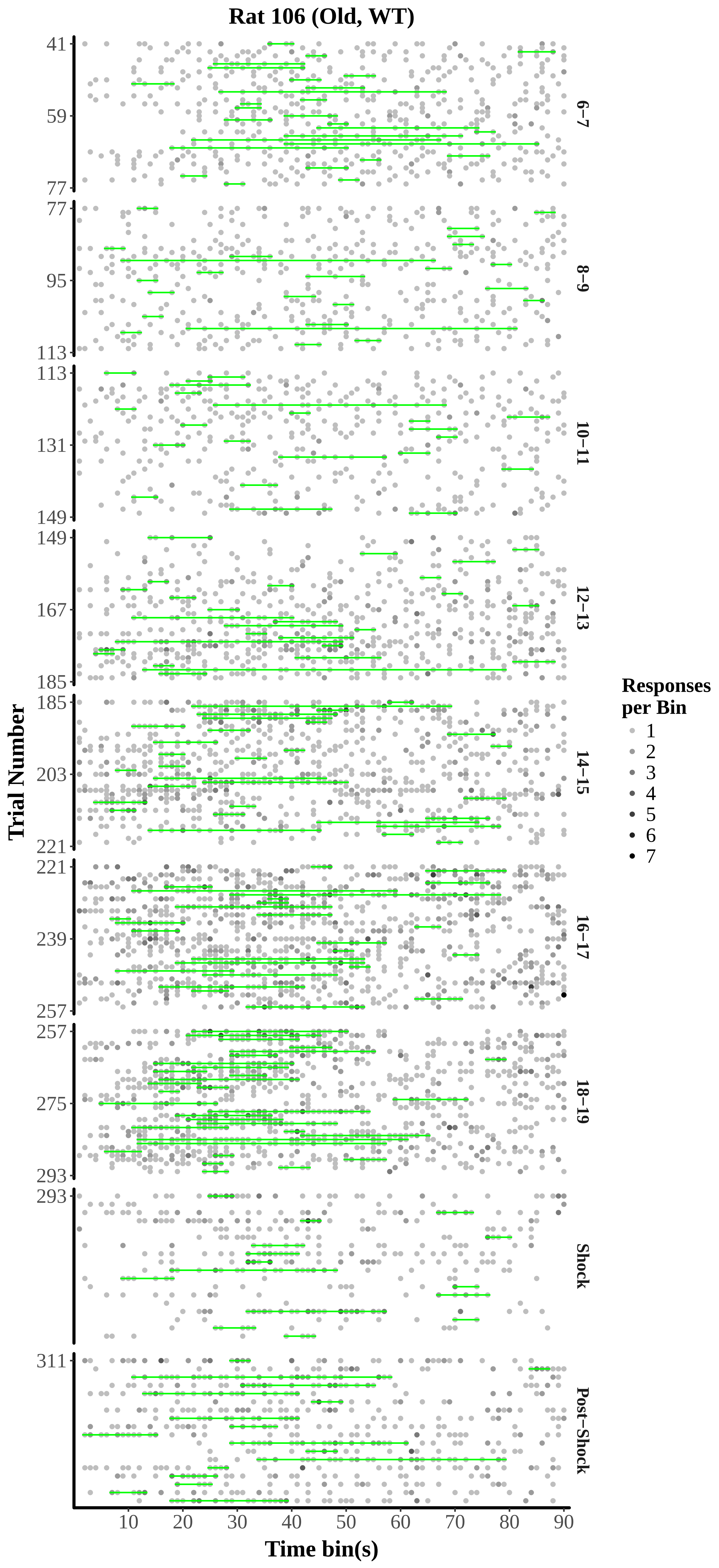

Rat 106 (Old, WT)
Trial Number
Time bin(s)
6–7
8–9
10–11
12–13
14–15
16–17
18–19
Shock
Post–Shock
Responses per Bin
1
2
3
4
5
6
7

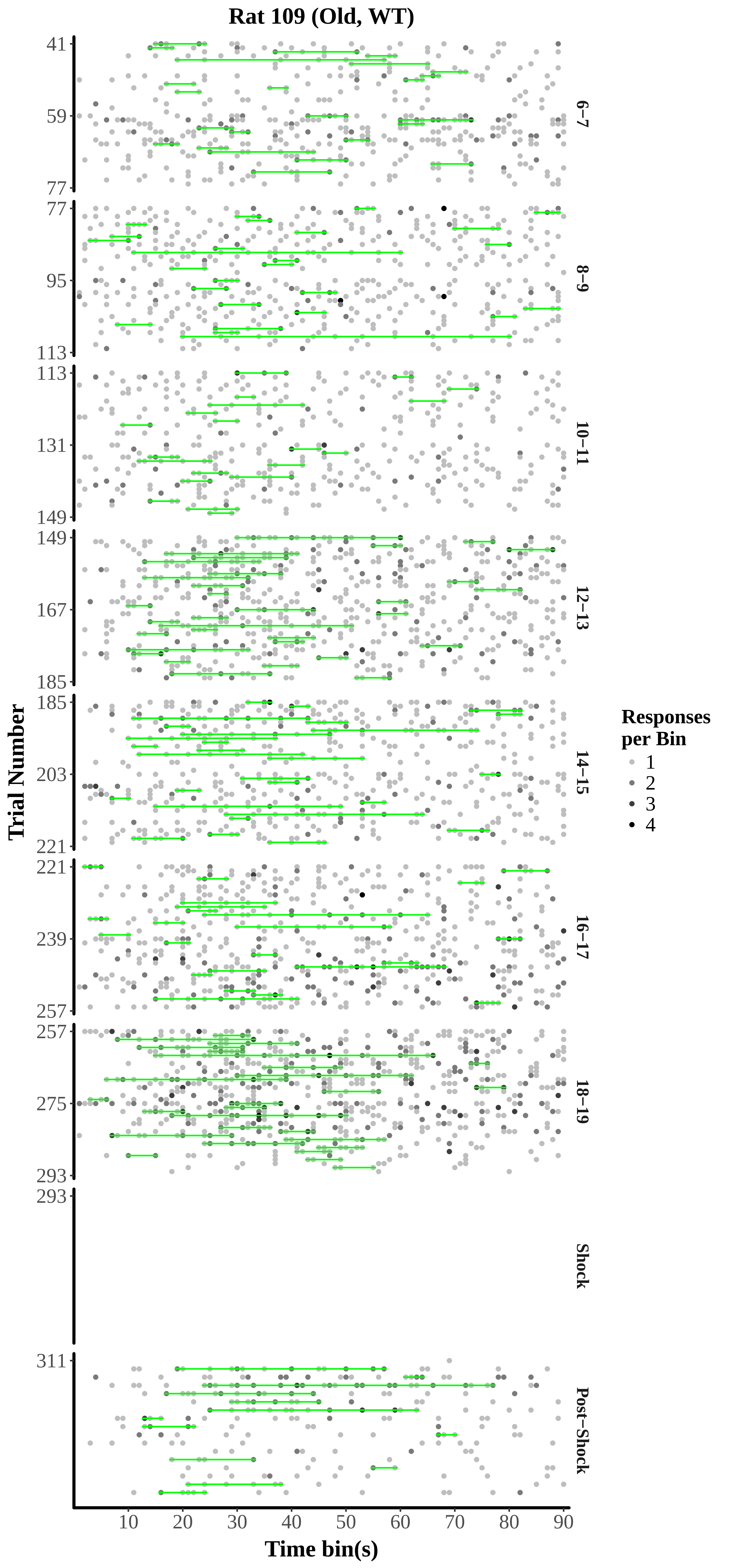

Rat 109 (Old, WT)
Trial Number
Time bin(s)
Responses per Bin
1
2
3
4
6–7
8–9
10–11
12–13
14–15
16–17
18–19
Shock
Post–Shock
41
59
77
95
113
131
149
167
185
203
221
239
257
275
293
311
10
20
30
40
50
60
70
80
90

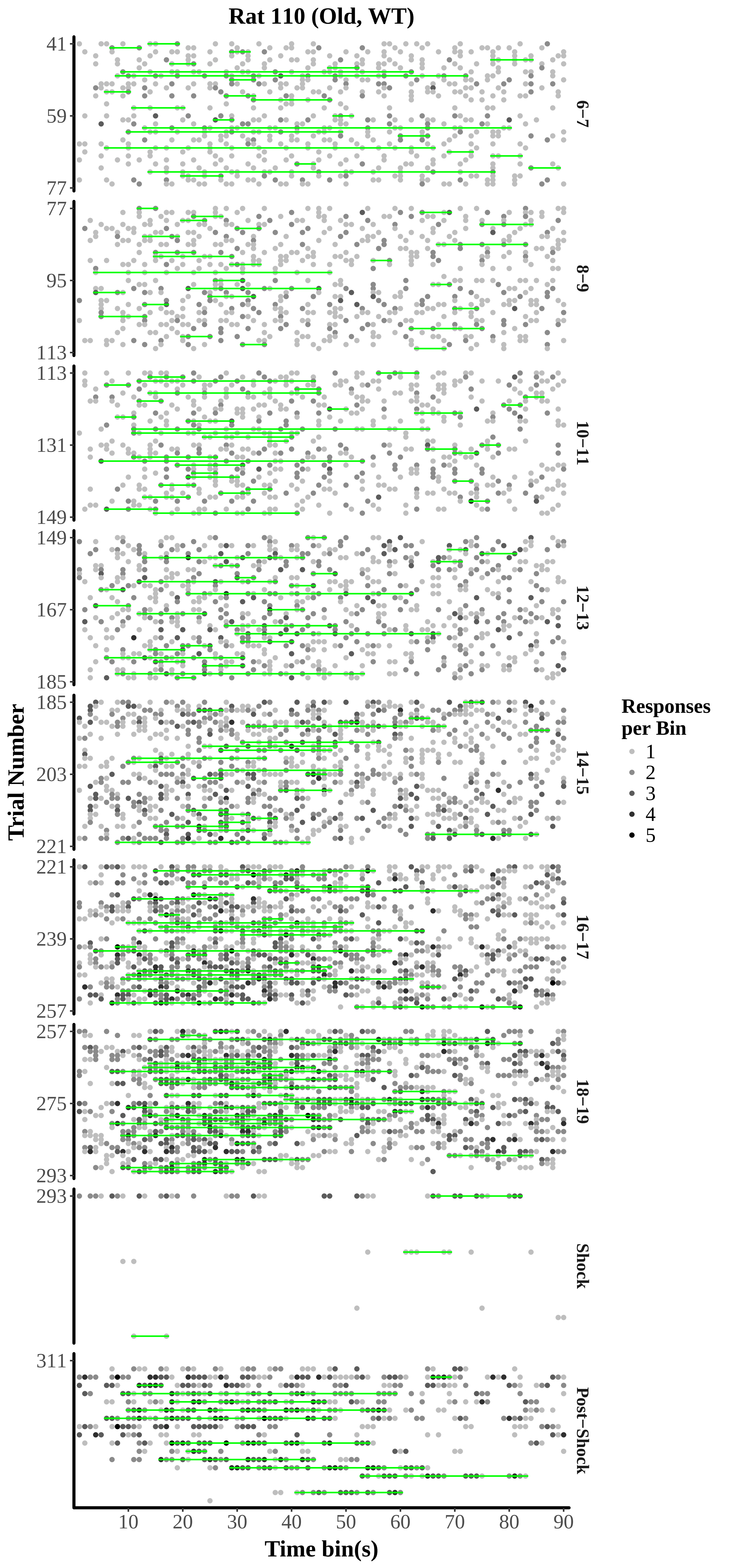
Rat 110 (Old, WT)
Trial Number
Time bin(s)
10
20
30
40
50
60
70
80
90
41
59
77
95
113
131
149
167
185
203
221
239
257
275
293
311
6–7
8–9
10–11
12–13
14–15
16–17
18–19
Shock
Post–Shock
Responses per Bin
1
2
3
4
5

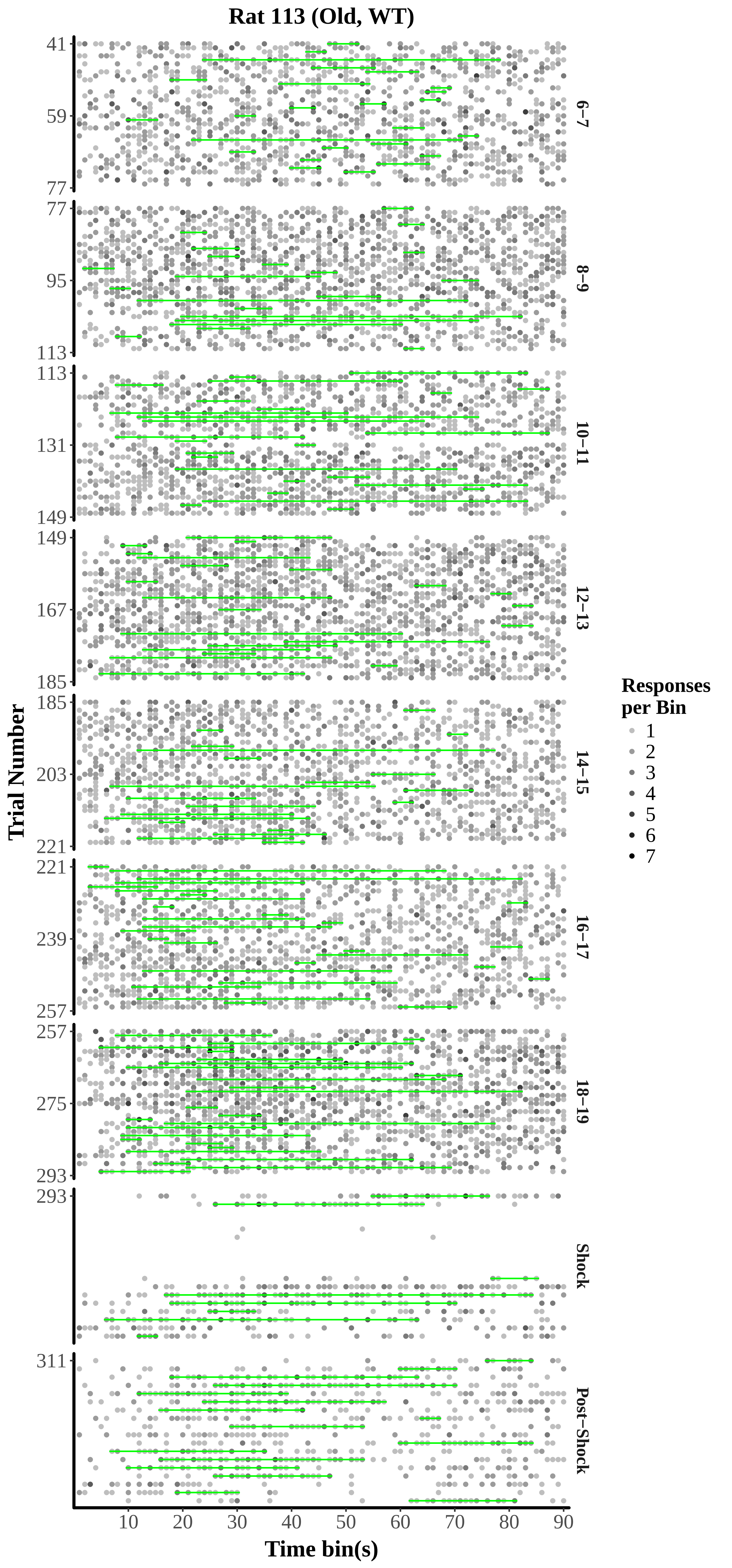

Rat 113 (Old, WT)
Trial Number
Time bin(s)
10 20 30 40 50 60 70 80 90
41 59 77
77 95 113
113 131 149
149 167 185
185 203 221
221 239 257
257 275 293
293
311
6–7
8–9
10–11
12–13
14–15
16–17
18–19
Shock
Post–Shock
Responses per Bin
1
2
3
4
5
6
7

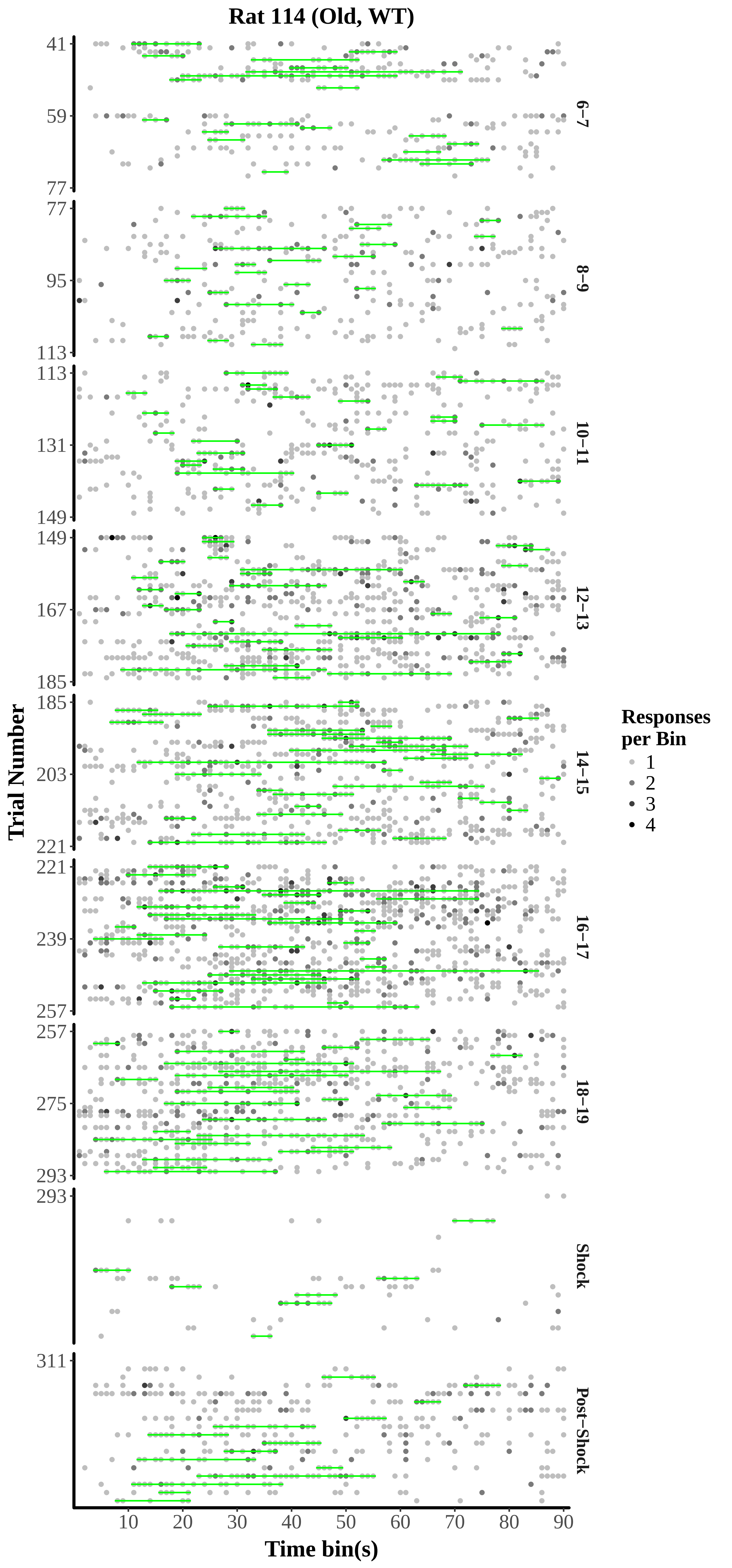
Rat 114 (Old, WT)
Trial Number
41
59
77
95
113
131
149
167
185
203
221
239
257
275
293
311
6–7
8–9
10–11
12–13
14–15
16–17
18–19
Shock
Post–Shock
Time bin(s)
10
20
30
40
50
60
70
80
90
Responses per Bin
1
2
3
4

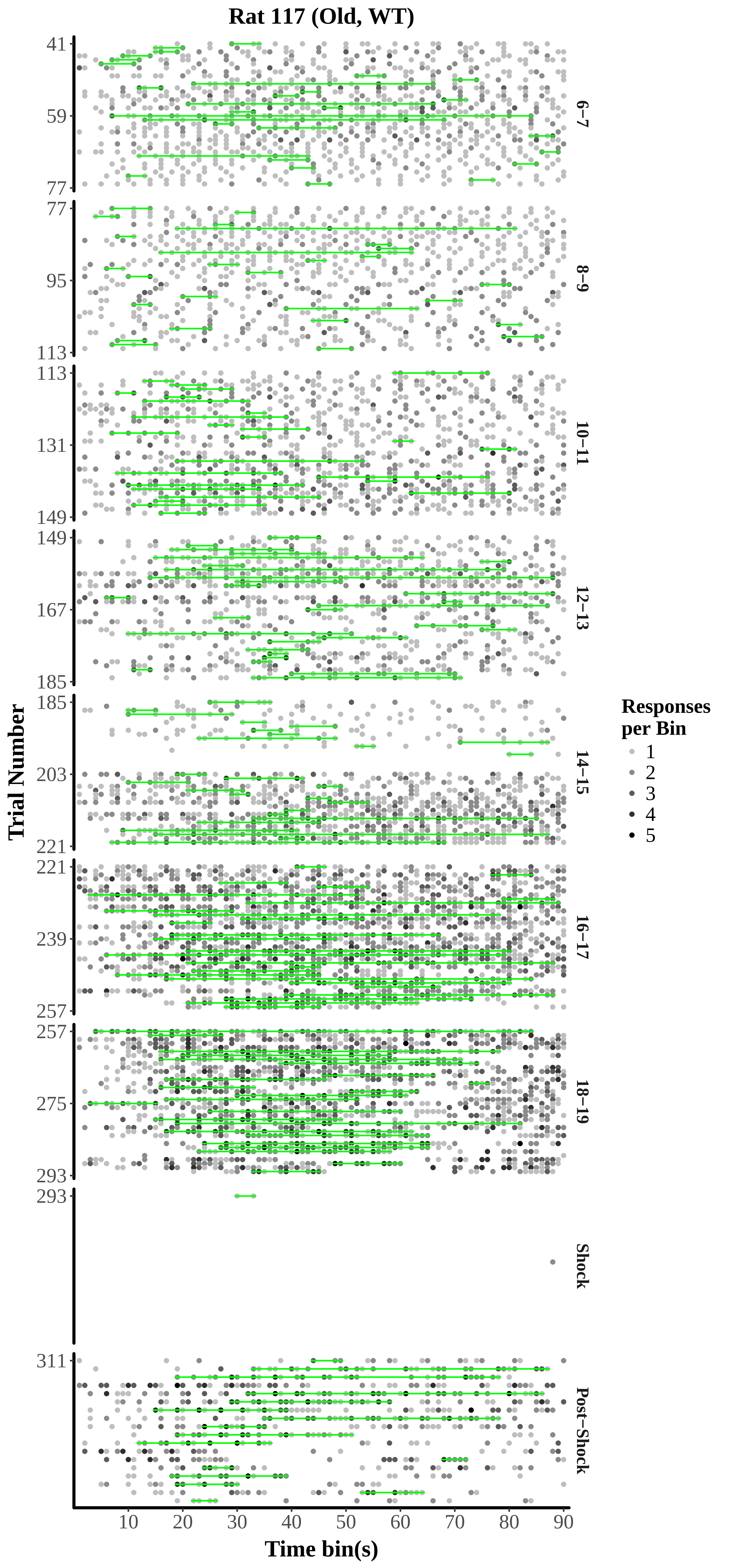
Rat 117 (Old, WT)
Trial Number
Time bin(s)
6–7
8–9
10–11
12–13
14–15
16–17
18–19
Shock
Post–Shock
41
59
77
95
113
131
149
167
185
203
221
239
257
275
293
311
10
20
30
40
50
60
70
80
90
Responses per Bin
1
2
3
4
5

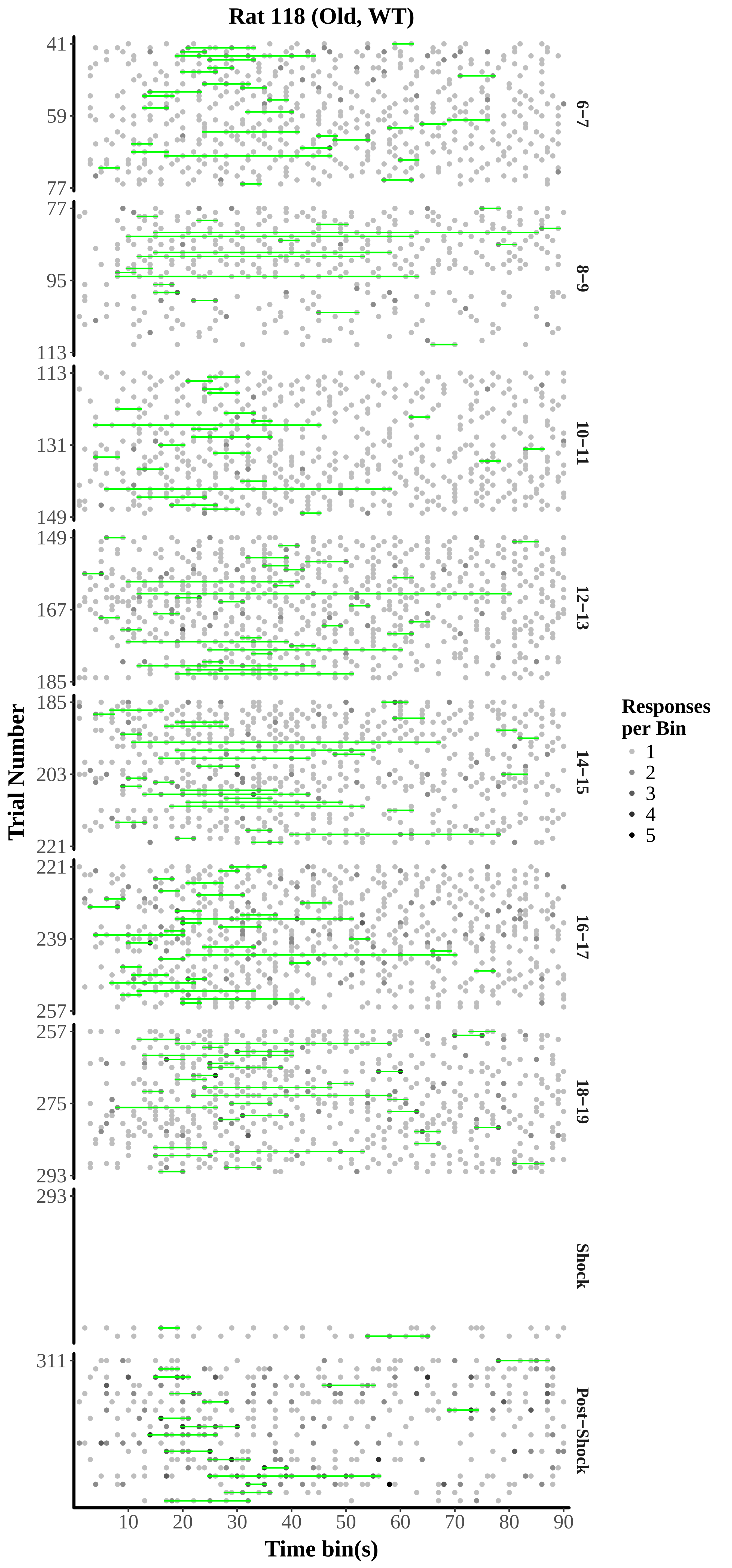

Rat 118 (Old, WT)
Trial Number
Time bin(s)
6–7
8–9
10–11
12–13
14–15
16–17
18–19
Shock
Post–Shock
Responses per Bin
1
2
3
4
5

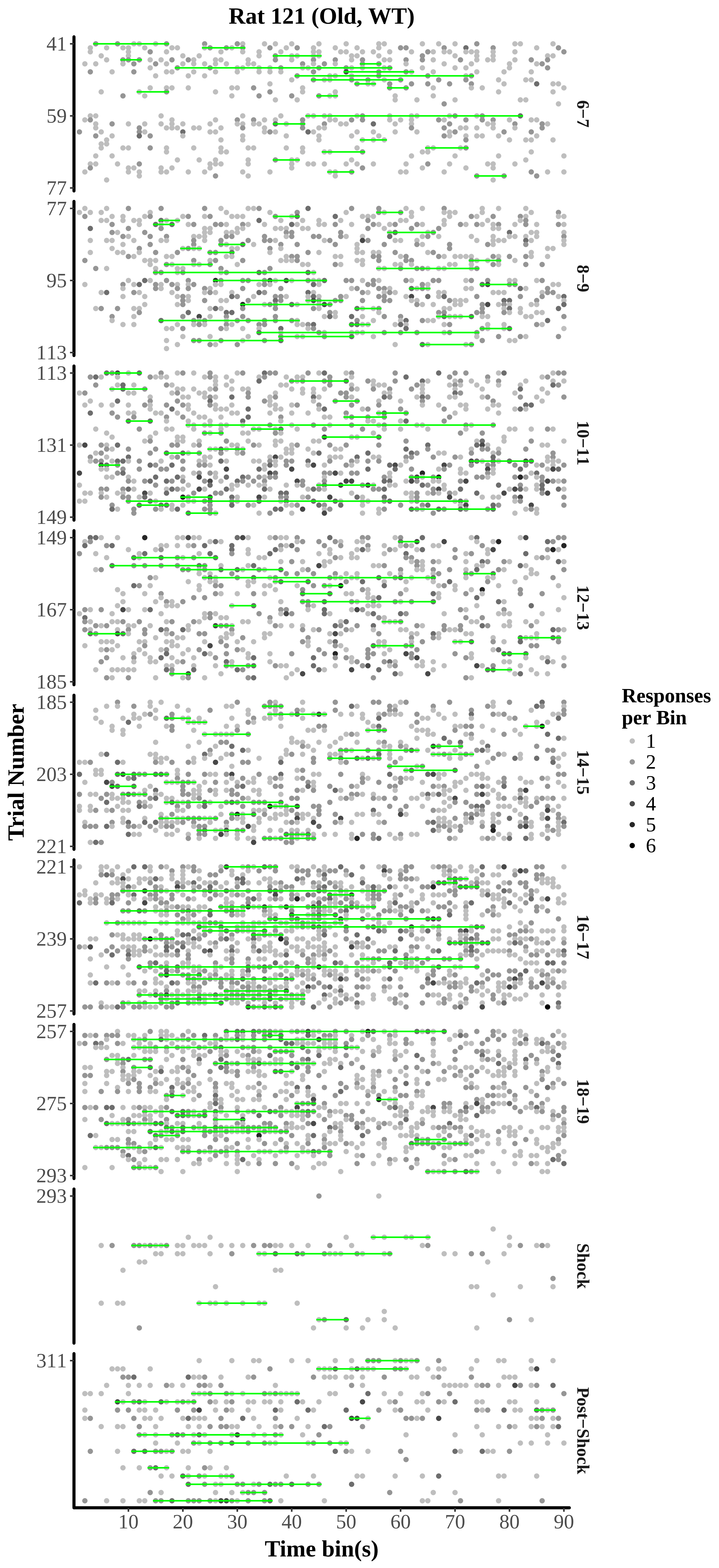

Rat 121 (Old, WT)
Trial Number
Time bin(s)
10 20 30 40 50 60 70 80 90
41 59 77
77 95 113
113 131 149
149 167 185
185 203 221
221 239 257
257 275 293
293
311
6–7
8–9
10–11
12–13
14–15
16–17
18–19
Shock
Post–Shock
Responses per Bin
1
2
3
4
5
6

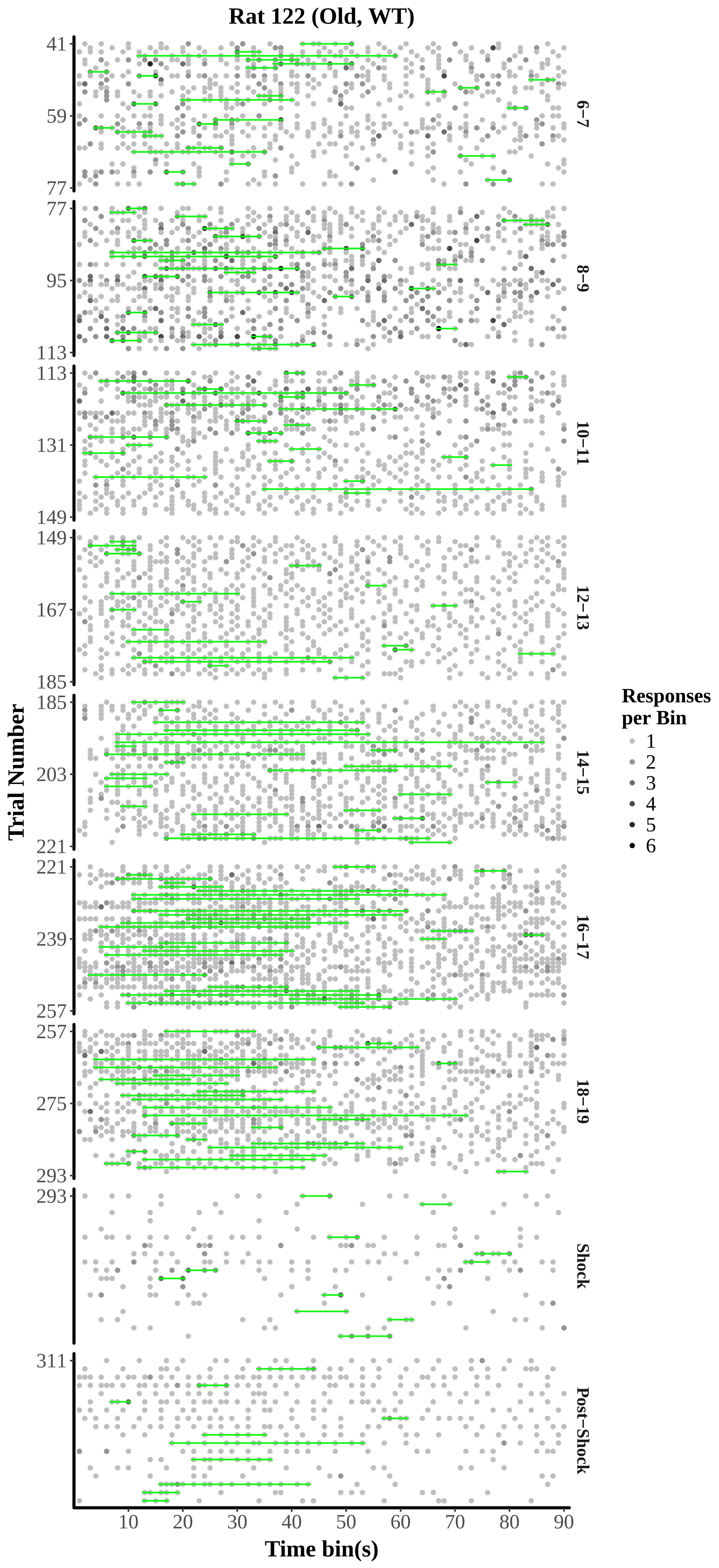

Rat 122 (Old, WT)
Trial Number
Time bin(s)
10
20
30
40
50
60
70
80
90
41
59
77
95
113
131
149
167
185
203
221
239
257
275
293
311
6–7
8–9
10–11
12–13
14–15
16–17
18–19
Shock
Post–Shock
Responses per Bin
1
2
3
4
5
6
